# A novel two-step administration of XPO-1 inhibitor may enhance the effect of anti-BCMA CAR-T in relapsed/refractory extramedullary multiple myeloma

**DOI:** 10.1186/s12967-023-04655-w

**Published:** 2023-11-15

**Authors:** Di Wang, Haiying Fu, Yimei Que, Haitao Ruan, Menglei Xu, Xiaolu Long, Qiuxia Yu, Chunhui Li, Zhe Li, Songbai Cai, Wei Chen, Cong Sun, Guang Hu, Shuai Wang, Donggou He, Jianming Mei, Wen Wang, Chunrui Li

**Affiliations:** 1 Department of Hematology, Tongji Hospital, Tongji Medical College, Huazhong University of Science and Technology, 1095 Jie-Fang Avenue, Wuhan, 430030 Hubei China; 2 Department of Hematology, The Third Affiliated People’s Hospital of Fujian University of Traditional Chinese Medicine, The Third People’s Hospital of Fujian Province, Fuzhou, China; 3 Immunotherapy Research Center for Hematologic Diseases of Hubei Province, Wuhan, 430030 Hubei China; 4 Nanjing IASO Biotherapeutics Ltd, Nanjing, 210032 Jiangsu China; 5 Antengene Corporation Ltd, Shanghai, 200051 China

**Keywords:** CAR-T, Selinexor, B cell maturation antigen, Extramedullary Multiple Myeloma

## Abstract

**Background:**

Extramedullary disease usually implies a dismal outcome in relapsed/refractory multiple myeloma patients, and requires novel treatment approaches. We designed a trial using Selinexor, a nuclear export protein 1 inhibitor, together with anti-B cell maturation antigen (BCMA) chimeric antigen receptor (CAR)-T cell product CT103A to treat these patients, and describe the first two cases in this report.

**Methods:**

Selinexor was administered with a novel two-step schedule in bridging therapy and in maintenance. The clinical responses and adverse events were recorded after CAR-T infusion and Selinexor administration. In vitro analysis of the influence of Selinexor on CAR-T cell function was performed using myeloma cell lines.

**Results:**

After infusion, both patients achieved stringent complete remission (sCR), and were maintained in sCR at data-cutoff, with survival over 13 and 10 months, respectively. Neither immune effector cell-associated neurotoxicity syndrome nor over grade 2 cytokine release syndrome was observed. Meanwhile, the patients showed good tolerance to the combination. In addition, we demonstrated that low dose of Selinexor could upregulate the expression of BCMA on plasma cell lines and subsequently enhance the function of CAR-T cell in vitro.

**Conclusions:**

The combination of Selinexor and CT103A exerts preliminary synergistic effect, and can be developed as a promising strategy for relapsed/refractory extramedullary myeloma.

**Supplementary Information:**

The online version contains supplementary material available at 10.1186/s12967-023-04655-w.

## Introduction

Extramedullary multiple myeloma (EMM) is an aggressive subtype of multiple myeloma (MM) with poor outcome. Generally, EMM can be roughly divided into extra-osseous and para-skeletal type. About 1.7% to 4.5% of newly diagnosed MM patients have extra-osseous lesions, and 7% to 34.4% have para-skeletal involvement; while in relapsed/refractory (R/R) patients, the incidence is 3.4% to 10% and 6% to 34.2%, respectively [1]. Plasma cell leukemia (PCL), recognized as a variant of EMM, has even more unfavorable prognosis [2]. The first-line treatment options for myeloma, including immunomodulatory drugs, proteasome inhibitors, and monoclonal antibodies, have shown limited efficacy in EMM patients [1, 3]. The newly developed chimeric antigen receptor (CAR)-T cell therapy successfully raises the response rate of EMM patients in recent reports. However, achieving a long-term survival still appears to be a critical challenge for these patients [4, 5]. Selinexor is a nuclear export protein 1 (XPO-1) inhibitor which has demonstrated gratifying results in heavily treated myeloma, including EMM patients [6]. Since Selinexor has good tissue penetration and acts through multiple anti-tumor mechanisms [7], the combination of Selinexor and CAR-T has the potential to act synergistically against the solid mass of EMM, and may further enhance the infiltration and persistence of CAR-T cells. Therefore, we initiated a clinical trial for R/R EMM patients to test the safety and efficacy of this combination. Here we report the result of the first two cases, with preliminary exploration of the possible mechanisms of action.

## Materials and methods

### Patient information

**Fig. 1:**
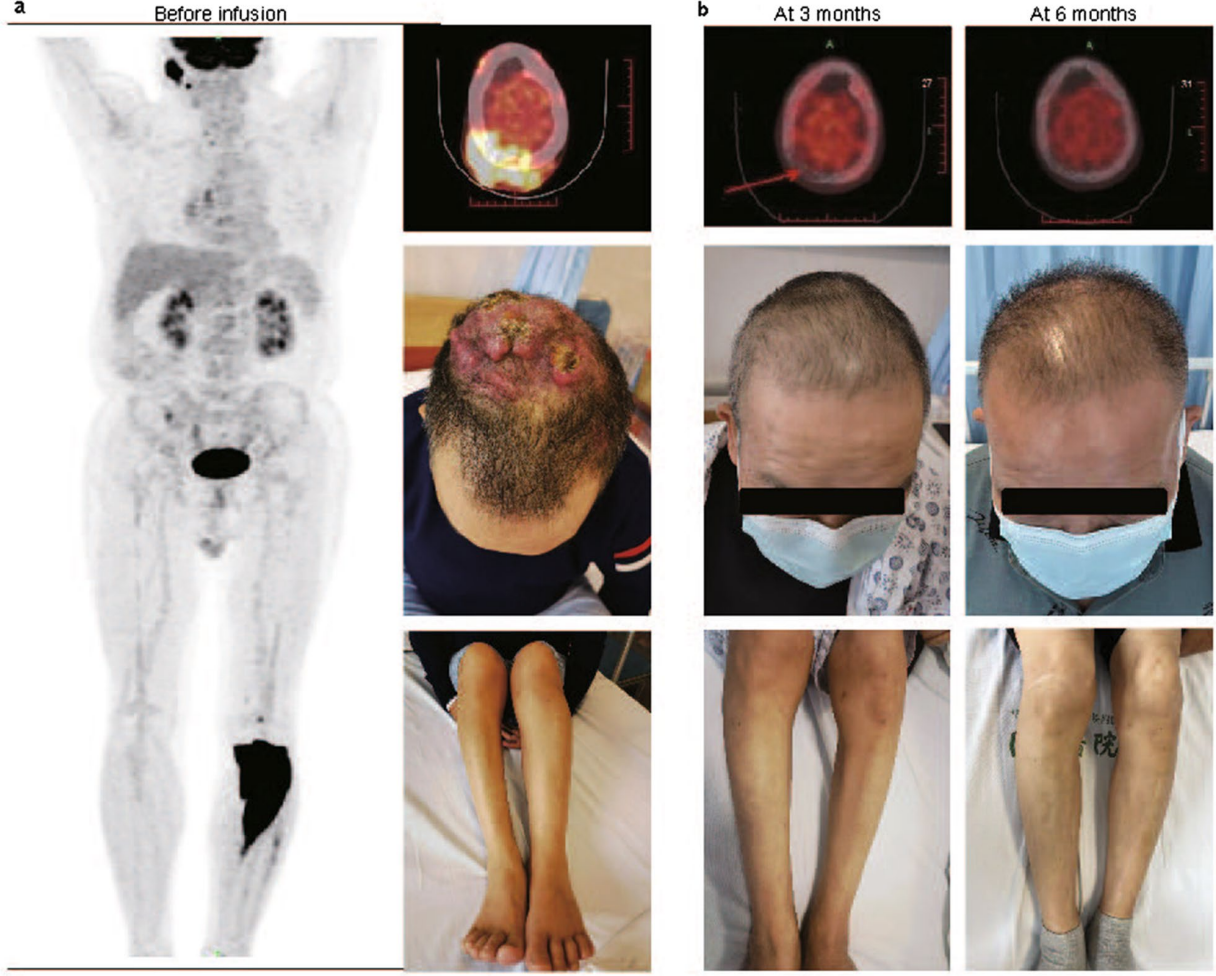
Extramedullary lesions in patient 1 before and after chimeric antigen receptor (CAR) -T cells infusion. **a** The results of combined positron emission tomography and computed tomography (PET-CT) with 18F-fluorodeoxyglucose demonstrated increased glucose metabolic activity in the calvaria and tibia of patient 1 before CAR-T infusion (left and up right). The gross appearance of soft tissue masses in the calvaria (right middle) and tibia (bottom right) were shown. **b** No lesion was observed for patient 1 by physical examination and PET-CT at 3 months and 6 months after infusion

Patient 1 was a 55-year-old male was referred to our center in September 2020. He was diagnosed with EMM one-year ago, and had received three lines of prior therapy with best response of complete remission (CR). He experienced extramedullary relapse and met the inclusion criteria. Positron emission tomography (PET) scan at baseline showed multiple lesions (Fig. 1a), which incapacitated him due to severe pain. Serum M protein (IgG-κ type) level was 2.1 g/L, but no plasma cells were detected in bone marrow or peripheral blood. The patient had decreased hemoglobin of 82 g/L, normal renal and cardiac functions, and had no high-risk cytogenetic features. Patient 2 was a 55-year-old female who came to the local hospital in July 2021 with lower back pain. An intraspinal mass spanning T12 to L2 was surgically resected, which was confirmed as a plasmacytoma. Over 60% plasma cells were discovered in her bone marrow and peripheral blood, secreting the IgD-λ type M protein. This patient received three lines of therapy with best response of minimal remission, and met the inclusion criteria. The baseline assessment showed over 80% of plasma blasts in the bone marrow, but no plasma cells in peripheral blood. No extramedullary mass was detected by PET scan. She had a large amount of bilateral pleural effusion (maximum depth: 9.9 cm and 13.4 cm on the left and right by ultrasonography, respectively), and 73% of nucleated cells in the effusion were plasma blasts. The M protein level was 38.8 g/L. The patient had decreased hemoglobin of 84 g/L, increased serum creatine of 144umol/L and pro-BNP of 370 pg/mL, and had no high-risk cytogenetic features.

### Study design

This registered trial (ClinicalTrials.gov Identifier: NCT05201118) was conducted at the Department of Hematology of Tongji Hospital in China. The trial protocol was reviewed and approved by the Institutional Review Board of Tongji Medical College, Huazhong University of Science and Technology. Written informed consent was obtained from each participant, in compliance with the Declaration of Helsinki. According to trial protocol, eligibility criteria included: age 18—70 years, an Eastern Cooperative Oncology Group performance-status score of 0 or 1, adequate major organ function, a life expectancy of 12 weeks or more, and at least three lines of prior therapies which must include a proteasome inhibitor and an immunomodulatory agent. After leukapheresis, the patients should receive a bridging therapy regimen that contain at least 40 mg of Selinexor per week. The bridging therapy must be stopped at least one week before lymphodepletion chemotherapy. The detailed description of the two cases at enrollment and their bridging therapy regimens were outlined in the supplementary material (Additional file 4). Patients received an FC lymphodepletion regimen (fludarabine at 30 mg/m^2^ and cyclophosphamide at 500 mg/m^2^ daily) for three consecutive days with one-day rest prior to CAR-T cell infusion. The CAR-T product used in this trial was CT103A, a fully human anti-B cell maturation antigen (BCMA) CAR that had demonstrated positive outcomes in our previous trial [8]. The dose of CT103A was 1 × 10^6^ /kg CAR^+^ T cells. After infusion, the first dose of Selinexor was scheduled to be given after platelets count had returned to 50 × 10^9^ /L. The dose was 40 mg or 60 mg per week, depending on the tolerance of each patient.

### Study evaluations

Serum and urine protein electrophoresis, quantitative immunoglobulin and serum free light-chain measurements, bone marrow/peripheral blood plasma cell count, and minimal residual disease assessment were performed at baseline, and serial points after infusion as indicated in the trial protocol. The extramedullary lesions were evaluated by PET scan at baseline, at day 90 and day 180. Clinical response was evaluated according to the 2016 International Myeloma Working Group consensus for MM [9] and 2013 consensus for PCL criteria [2]. CAR transgene copies in the patient PBMCs were monitored by digital droplet polymerase chain reaction as previously described [8]. The data-cutoff date was Dec 31, 2022. The grading of cytokine release syndrome (CRS) and immune effector cell-associated neurotoxicity syndrome (ICANS) was according to the criteria of Lee et al. [10]. All adverse events (AE) were recorded throughout the follow-up, and were graded by the National Cancer Institute Common Terminology Criteria for Adverse Events (CTCAE) Version 5.0 (https://academy.myeloma.org.uk/wp-content/uploads/sites/2/2015/04/CTCAE_v5.pdf).

### Exploratory study

Multiple myeloma cell lines MM1S and U266 were pre-treated with Selinexor at 0—100 nM for 24 h and washed by additional culturing medium before CAR-T cells were added. The expression of BCMA on both cells was evaluated by flow cytometry after Selinexor pre-treatment. These pre-treated cell lines were then cultivated with CAR-T cells for an additional 24 h. Assessment of the cytotoxicity and viability of CAR-T cells was performed via flow cytometry.

## Results

### Clinical response

The mass on the head of patient 1 shrunk markedly during the first two weeks post infusion. M protein was not detectable at day 30 and he continued to remain free of detectable plasma cells. At the third month, the mass was no longer visible and PET scan showed a complete metabolic inactivation in the lesions of his head and lower limb (Fig. 1b), indicating that he achieved stringent complete remission (sCR).

For patient 2, about 1000-1500 ml pleural effusion was drained per day by a pleural catheter during the first week post infusion. The amount of drainage gradually decreased from day 8, and was minimal at day 21. Plasma cells were not detected by flow cytometry in bone marrow or peripheral blood from day 30. Computerized tomography (CT) scan showed no more pleural effusions at day 60 (Additional file 1: Fig. S1). The M-protein was undetectable by day 90 (Additional file 2: Fig. S2), indicating that patient 2 achieved sCR.

Both patients remained in sCR state at the time of data cut-off, with survival over 13 and 10 months, respectively.

### Adverse events

Adverse events occurred post-infusion and after Selinexor maintenance were separately recorded (Table 1). Patient 1 experienced fever and transient hypoxemia, reflecting a grade 2 CRS. These symptoms were controlled by oral loxoprofen and oxygen inhalation of 3L/min without glucocorticoid or tocilizumab. No ICANS was observed. The patient experienced grade 4 thrombocytopenia and leukopenia. Both AEs recovered in 14 days. The patient had cytomegalovirus reactivation (9.65 × 10^2^ copies/mL) at day 22, which was found negative at day 45 after receiving ganciclovir intravenously. He also had pulmonary infection from day 28 to day 45, confirmed by CT. Therefore, Selinexor maintenance was not started until day 49 with dose of 40 mg per week. After Selinexor administration, the patient complained of grade 2 loss of appetite and fatigue, grade 3 neutropenia, and grade 2 anemia.Patient 2 experienced continuous high fever for three days, which cannot be controlled by antipyretics such as loxoprofen. So 40mg methylprednisolone was given for four consecutive days to treat CRS, but the CRS grade remained in level 1 as there was no hypoxemia or hypotension recorded. No ICANS was observed. The patient experienced Grade 4 thrombocytopenia and leukopenia after infusion and the thrombocytopenia lasted till day 78. She also experienced herpes zoster reactivation and urinary tract infection during the first three months post-infusion. Selinexor was finally given at 20mg per week from day 90. After Selinexor administration, the patient complained of grade 1 weight loss, fatigue, and other gastrointestinal AEs.

**Table 1:** Adverse events occurred post-infusion and after Selinexor maintenance

Patient	Post CT103A infusion	Grade	After Selinexor use	Grade
Patient 1	CRS	2	Loss of appetite	2
	Fever	1	Fatigue	2
	Hypoxemia	1	Neutropenia	3
	Neutropenia	4	Anemia	2
	Lymphopenia	4		
	Anemia	2		
	Thrombocytopenia	4		
	Hypoglobulinemia	1		
	Pulmonary infection	3		
	CMV reactivation	3		
Patient 2	CRS	1	Loss of appetite	1
	Fever	1	Fatigue	1
	Neutropenia	4	Diarrhea	1
	Lymphopenia	4	Weight loss	1
	Anemia	3	Nausea	1
	Thrombocytopenia	4	Neutropenia	3
	Elevated LDH	1	Anemia	2
	Hypoalbuminemia	2	Thrombocytopenia	1
	Hypoglobulinemia	1		
	Hypokalemia	2		
	Herpes zoster reactivation	3		
	Urinary tract infection	3		

*CRS* cytokine release syndrome, *CMV* cytomegalovirus, *LDH* lactate dehydrogenase

Up to the data-cutoff date, no other hematological toxicity except for B lymphocytopenia was observed in the two patients. Intermittent gamma globulin infusions were given to both patients.

### Monitoring of CT103A and inflammatory cytokines

**Fig. 2:**
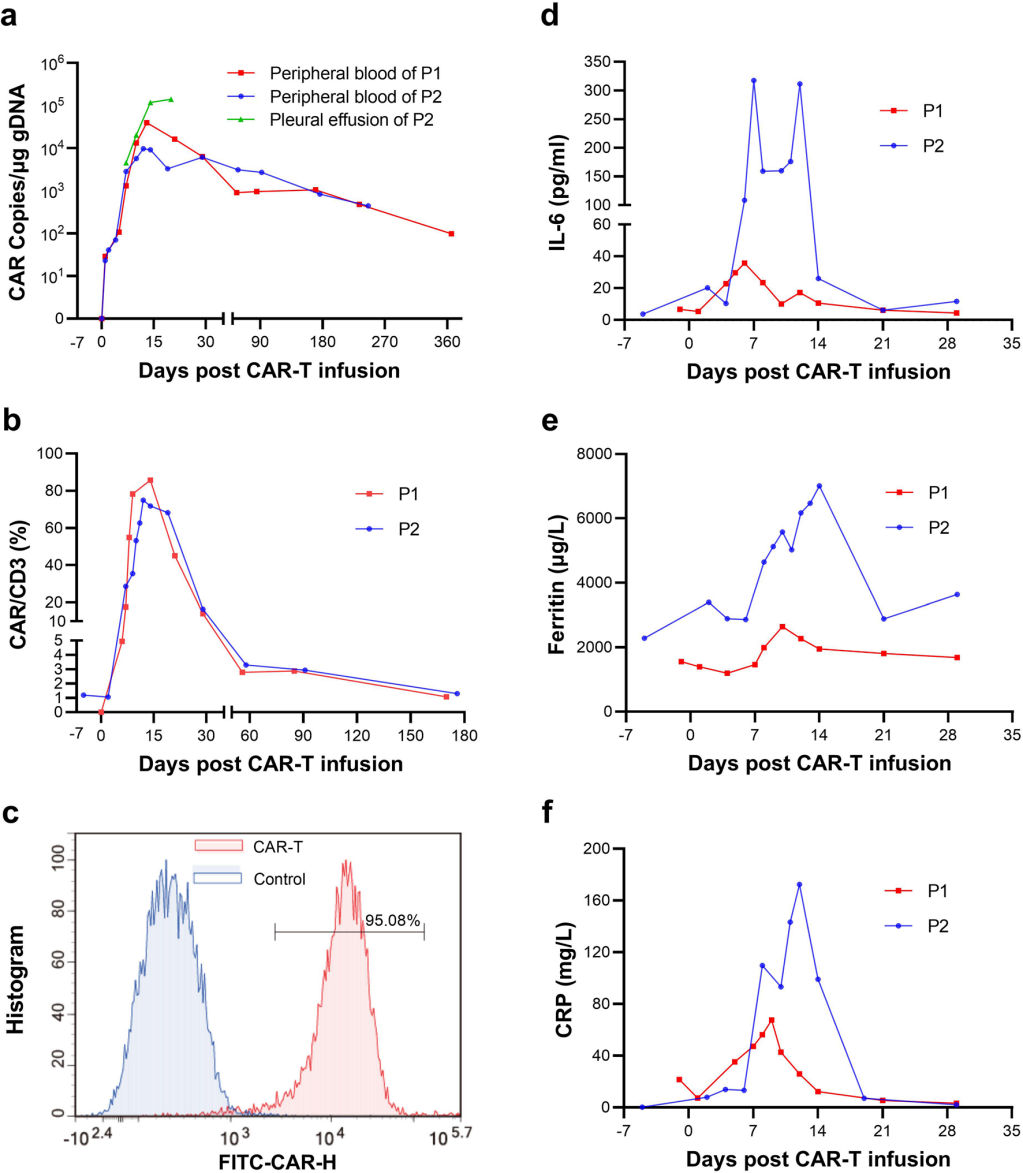
CAR-T cell expansion, and change of inflammatory indicators post infusion. **a** The CAR transgene copies of mononuclear cells from peripheral blood for patient 1 (in red), and from peripheral blood (in blue) and pleural effusion (in green) for patient 2 was detected by digital PCR at serial points post infusion. The T_max_ of CAR transgene in peripheral blood for the two patients was 13 and 12 days, respectively. The C_max_ of CAR transgene in peripheral blood was 39,670 and 9707 copies/μg gDNA, respectively. **b** The quantity of CAR^+^ and CD3^+^ T cells were verified by flow cytometry. The change of CAR^+^/CD3^+^ T cells at serial points post infusion was in accordance with CAR transgene copies. The peak of CAR^+^ cell proportion occurred at around two weeks. **c** A high proportion (95.08%) of CAR-T cell infiltration was detected by flow cytometry in the pleural effusion of patient 2. The serum levels of interluekin-6 **d**, hypersensitive C reactive protein (**e**), and ferritin (**f**) at serial points post infusion was recorded. All these three factors rose to the peak at around day 7 in patient 1 and day 14 in patient 2, and then decreased, which was also in accordance with the change of CAR transgene copies. Abbreviations: C_max_: the peak value of CAR transgene copies; hsCRP, hypersensitive C reactive protein PCR, polymerase chain reaction; T_max_, the time of CAR transgene copies to reach the peak

The cytokinetics of CAR^+^ T-cells was monitored by measuring CAR transgene copies in peripheral blood (Fig. 2a). After infusion, the time of CAR transgene copies to reach the peak (T_max_) was 13 and 12 days, respectively. The peak value of CAR transgene copies (C_max_) was 39,670 and 9707 copies/μg gDNA, respectively. The CAR transgene was still detectable at their last follow-up. We detected an increase of CAR transgene copy in the pleural effusion of patient 2, which was noticeably higher than that in peripheral blood, until there was no effusion available for evaluation. The change of proportion of CAR^+^ to CD3^+^ T cells were shown in Fig. 2b, which was consistent with the trend of CAR transgene. A high proportion (95.08%) of CAR-T cells was detected by flow cytometry in the pleural effusion of patient 2 on day 14 (Fig. 2c). The inflammation related biomarkers, including ferritin, interleukin-6, and hypersensitive C reactive protein also showed a rise in the first two-week post infusion, reached the peak, and then decreased to normal (Fig. 2d-2f), similar with CAR-T cell expansion. Compared with patient 1, patient 2 had relatively higher levels of inflammatory indicators.

### Influence of Selinexor on CAR-T and plasma cells in vitro

**Fig. 3:**
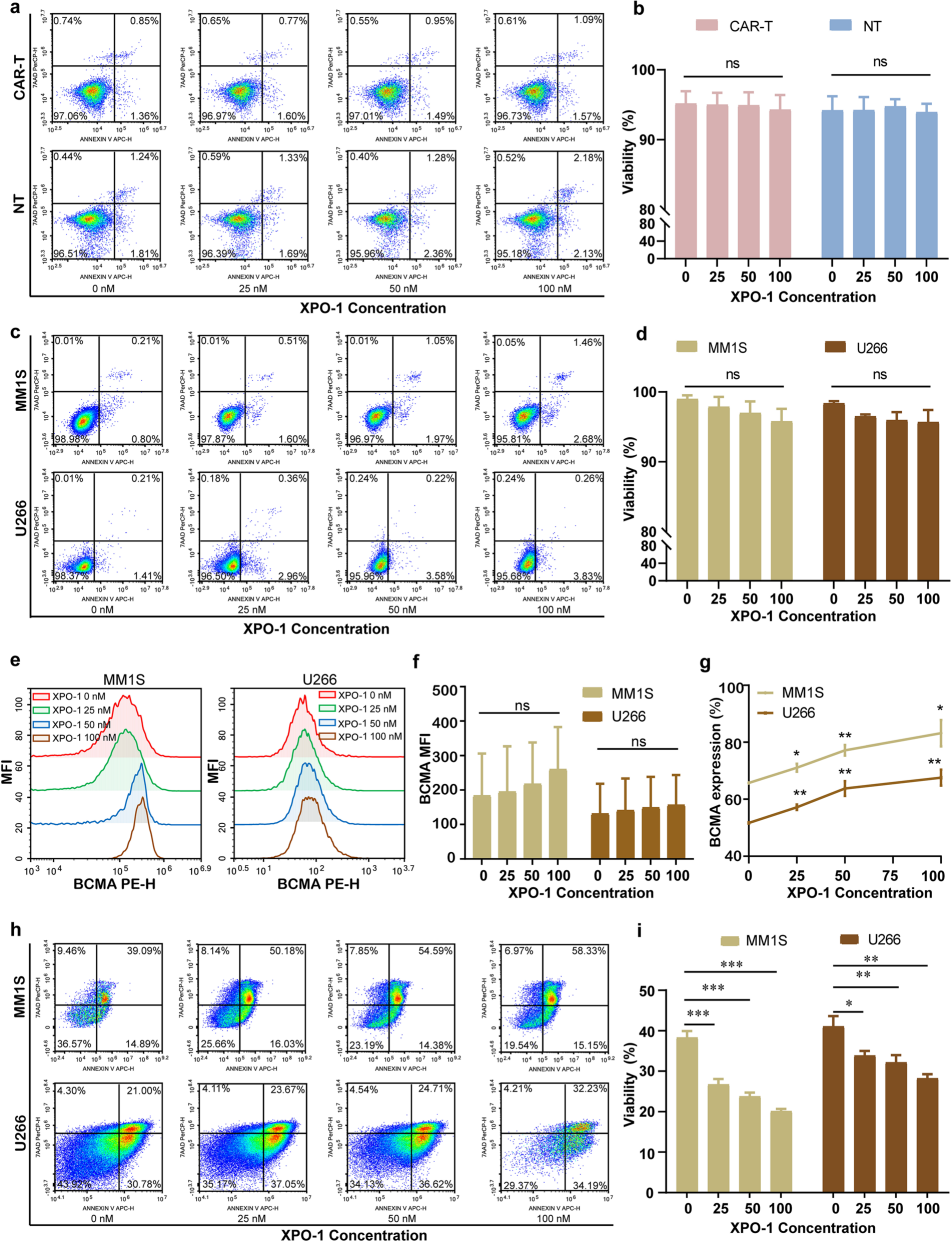
Influence of Selinexor on plasma cell and CAR T-cell in vitro. **a**, **b** Flow cytometry was used to assess the viability of CAR-T (CT103A) cells and NT (negative control) cells in different concentrations of Selinexor (0-100 nM). There was no difference observed between different dose groups. **c**, **d** Flow cytometry was used to assess the viability of MM1S and U266 cell lines in different concentrations of Selinexor (0-100 nM). No difference was observed between different dose groups. **e**–**g** Flow cytometry was used to evaluate MFI and expression rate of BCMA on MM1S and U266 cell lines pre-treated with different concentrations of Selinexor (0-100 nM). There was a slight but not statistically significant increase in MFI. Meanwhile, there was a significant increase of expression rate of surface BCMA with dose escalation. **h** Flow cytometry was used to assess the viability of different concentrations of Selinexor (0-100 nM) pre‑treated MM1S and U266 cell lines when co-cultured with CAR-T cells. **i** The statistical representation of diagram h. The cytotoxicity increased in a dose-dependent manner, which was statistically significant. Abbreviations: BCMA, B cell maturation antigen; MFI, mean fluorescence intensity; ns, no significance; XPO-1, nuclear export protein 1 inhibitor Selinexor. *Statistically significant with p < 0.05, **p < 0.01, and ***p < 0.0001

The viability of CAR-T cells was not affected by Selinexor at low concentration (≤ 100 nM) (Fig. 3a and b), and same results were observed on the MM cell lines MM1S and U266 (Fig. 3c and d). Interestingly, the surface expression rate of BCMA on tumor cells was found to be increased in a dose-dependent manner by treatment with Selinexor (Fig. 3e-g). Tumor cells pre‑treated with Selinexor were also more susceptible to cytotoxicity from CAR-T cells (Fig. 3h and i).

## Discussion

We report two cases of patients with relapsed/refractory EMM treated with the combination of Selinexor and anti-BCMA CAR-T cell therapy. Both patients achieving deep and durable response. In comparison, previous CAR-T studies have shown that responses of EMM patients were unsatisfactory, with CR rates lower than 60% and a median progression-free survival of no more than 9 months [5, 11, 12]. The limited efficacy may be related to the biological characteristics of the solid extramedullary mass, a hostile tumor microenvironment (TME), and the high heterogeneity of tumor cells.

**Fig. 4:**
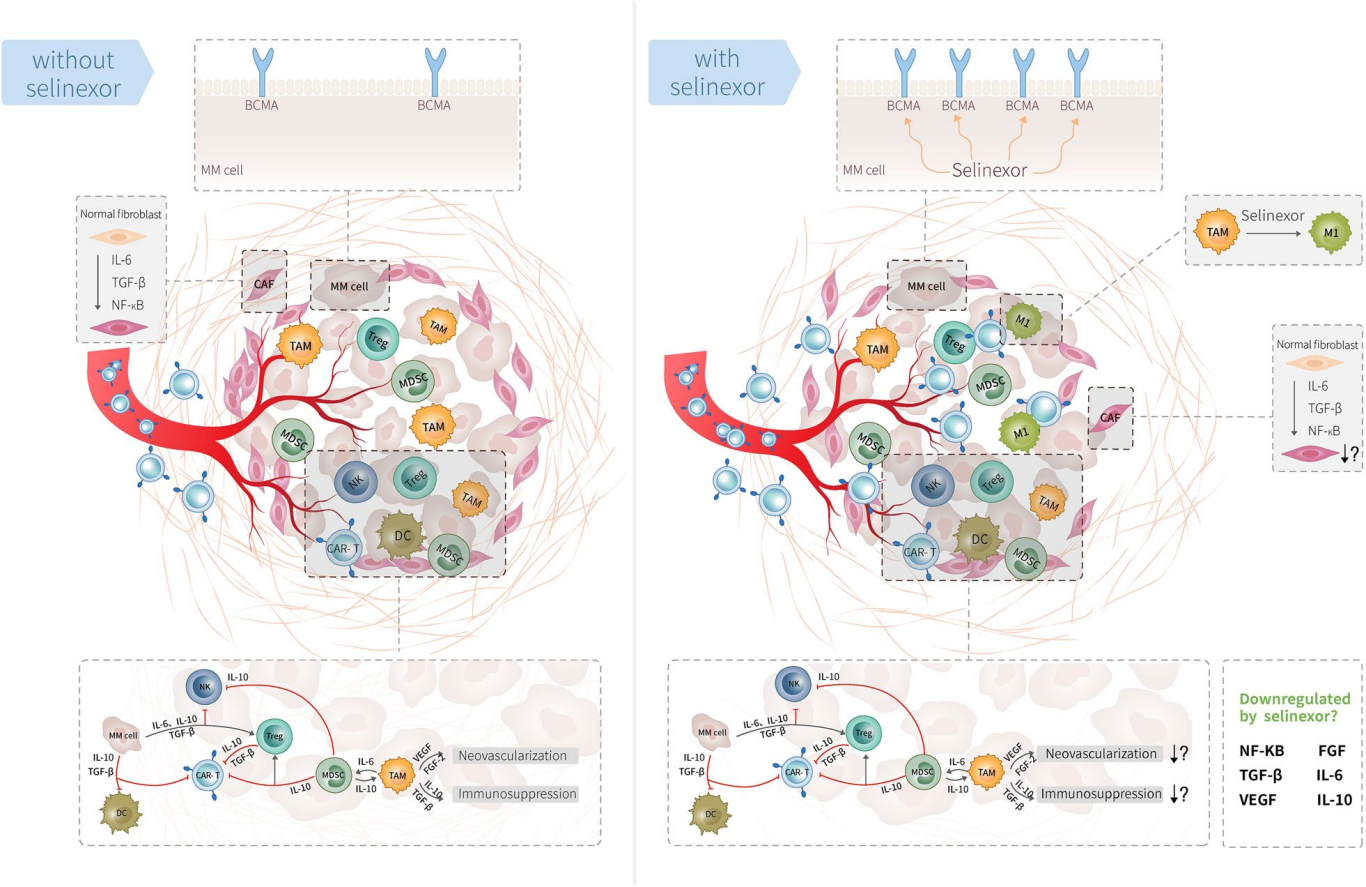
Potential mechanisms of Selinexor on tumor cells and microenvironment of EMM to promote CAR-T function. Left part: Without Selinexor, CAR-T cell function can be restricted by the insufficient BCMA expression on myeloma cells, as well as the tumor microenvironment, including physical barriers of tumor (vascular and extracellular matrix disorder), and immunosuppressive factors (e.g., TAM, MDSC, Treg, and their secreted cytokines). Right part: After the use of Selinexor, the BCMA expression on myeloma cells can be upregulated, repolarization of M2-like macrophages is promoted and the number of M1-like macrophages increases. For the immunosuppressive cells such as CAFs and Treg, and suppressive cytokines such as TGF-β and NFκB, the influence of Selinexor remains to be explored. Selinexor might have the potential to facilitate CAR-T cell infiltration and expansion if remodulation of the immunosuppressive microenvironment is possible. Abbreviations: CAF, cancer-associated fibroblast; DC, dendritic cell; FGF, fibroblast growth factor; IL, interleukin; NF, nuclear factor; MDSC, myeloid-derived suppressor cells; MM, multiple myeloma; NK, natural killer; TAM, tumor-associated macrophages; TGF, tumor growth factor; Treg, regulatory T cells; VEGF, vascular endothelial growth factor

As illustrated in the left part of Fig. 4, the tumor microenvironment can restrict the function of CAR-T cells through several aspects, including physical barriers, immunosuppressive factors, and CAR-T cell exhaustion [13]. In previous reports, CAR-T cells were shown to be capable of penetrating the blood brain barrier and blood testicular barrier [14]. Interestingly, our second patient in this study had tumor infiltration in the pleura, and we detected a ten-fold higher CAR transgene copy in her pleural effusion. This unusual high CAR transgene copy demonstrated that tissue penetration of CT103A might be enhanced. Nevertheless, in a solid tumor mass, the tumor vascular dysregulation and extracellular matrix with dense fibrogen may protect the tumor from CAR-T cells [13]. Additionally, immunosuppressive factors, such as immune checkpoints, tumor growth factor-β (TGF-β), tumor-associated macrophages, and regulatory T cells can impede the function of CAR-T cells [15]. The effect of CAR-T therapy may be enhanced by overcoming one or more of these factors. For instance, the local delivery of CAR-T cells in solid tumors increase the chance of contact; the use of engineered CAR-T cells with PD1 knockdown may reduce CAR-T cell exhaustion [16].

As XPO-1 may affect multiple oncogenic nuclear proteins, its inhibition by Selinexor may allow changes in the TME that facilitate CAR-T cell infiltration and expansion. For example, tumor infiltrating macrophages can be repolarized into M1 in mouse central nervous system lymphoma by combination of Selinexor and BTK inhibitors [17]. The expression of immunosuppressive factor TGF-β may also be affected by XPO-1, since one of its upstream regulators, cycling D1, is a cargo of XPO-1 [18].

Insufficient expression of target antigen is another main cause of CAR-T treatment failure [19]. Some approaches can upregulate the target antigen expression to enhance CAR-T efficacy, as reported for upregulation of BCMA by a γ-secretase inhibitor in myeloma [20], and GD2 upregulation upon EZH2 inhibition in Ewing Sarcoma [21]. Notably, we demonstrated that low-concentrations of Selinexor can increase the expression of BCMA antigen on plasma cells in vitro, which may help CAR-T cells to better recognize tumor cells. Eltanexor, another selective inhibitor of nuclear export (SINE) compound, demonstrated the capability of enhancing cytotoxicity, and impeding the exhaustion of CAR-T cells as well [22]. On the other hand, toxicity of Eltanexor can negatively impact CAR-T cell activity, which may limit the potential for the simultaneous use of Eltanexor with CAR-T therapy. However, for Selinexor, we demonstrated that the apoptosis of CAR-T cells was not increased at low-concentrations in vitro. In addition, Tyler et al. reported that although high frequency of Selinexor dosing can impair T cell function, once or twice weekly dosing schedules allow for normal CD8^+^ T cell functioning and development of antitumor immunity [23]. Therefore, the combination of Selinexor and CAR-T seems more feasible.

Accordingly, the administration of Selinexor was subtly designed to into two steps in this study. Firstly, Selinexor was used in the bridging therapy and was discontinued one week before lymphodepletion. And then, it was used as a single drug maintenance therapy with a lower dose of 20 or 40 mg per week after the recovery of platelet. The BCMA expression can be upregulated by the first administration, and TME might be altered through both steps, thereby might promote the infiltration, expansion and persistency of CAR-T cells. These possible mechanisms of tumor microenvironment remodulation are outlined in the right part of Fig. 4. For tumor-associated macrophages, Selinexor promotes repolarization of M2-like macrophages and increases the number of M1-like macrophages, which minimizes the tumor promotion effect of M2-Like macrophages [17]. For the immunosuppressive cells and cytokines, the influence of Selinexor was unknown but yet expectable. Thereby more research is needed to verify the potential benefits from Selinexor on the tumor microenvironment.

Although the exact mechanisms of potential synergy need further definition, the robust response observed in the two patients demonstrates that Selinexor may act to improve the effect of CAR-T cells.

## Conclusion

The combination of two-step Selinexor dosing and CT103A appears to be a promising strategy to treat EMM patients. In the ongoing study, we will further observe the treatment safety and efficacy, and attempt to elucidate more clearly the underlying synergistic mechanisms.

### Supplementary Information

**Additional file 1.** **Fig. S1.** Change of pleural effusion in patient 2 before and after treatment.

**Additional file 2.** **Fig. S2.** Change of M protein and serum free light chain in patient 2 before and after treatment.

**Additional file 3.** Trial protocol.

**Additional file 4.** Supplemental Materials.

## Data Availability

The datasets used and/or analyzed during the current study are available from the corresponding author on reasonable request.
