# Supplementary figures and images for: A novel two-step administration of XPO-1 inhibitor may enhance the effect of anti-BCMA CAR-T in relapsed/refractory extramedullary multiple myeloma
Source: J Transl Med. 2023 Nov 15;21:812. doi: 10.1186/s12967-023-04655-w (PMC10647128; doi:10.1186/s12967-023-04655-w)

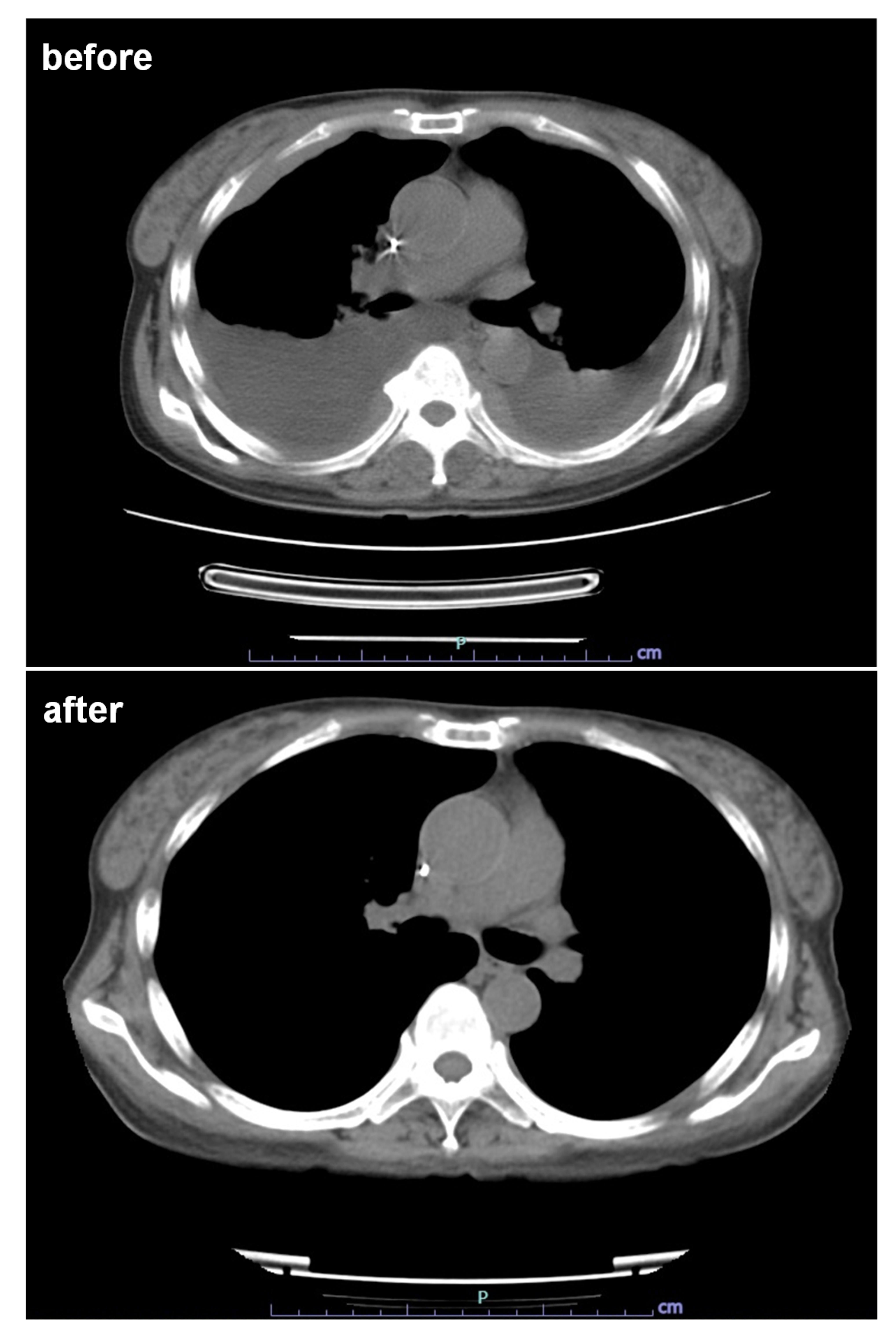

Supplement: Supplementary file 1 — Additional file 1. Fig. S1. Change of pleural effusion in patient 2 before and after treatment. [file 12967_2023_4655_MOESM1_ESM.jpg]

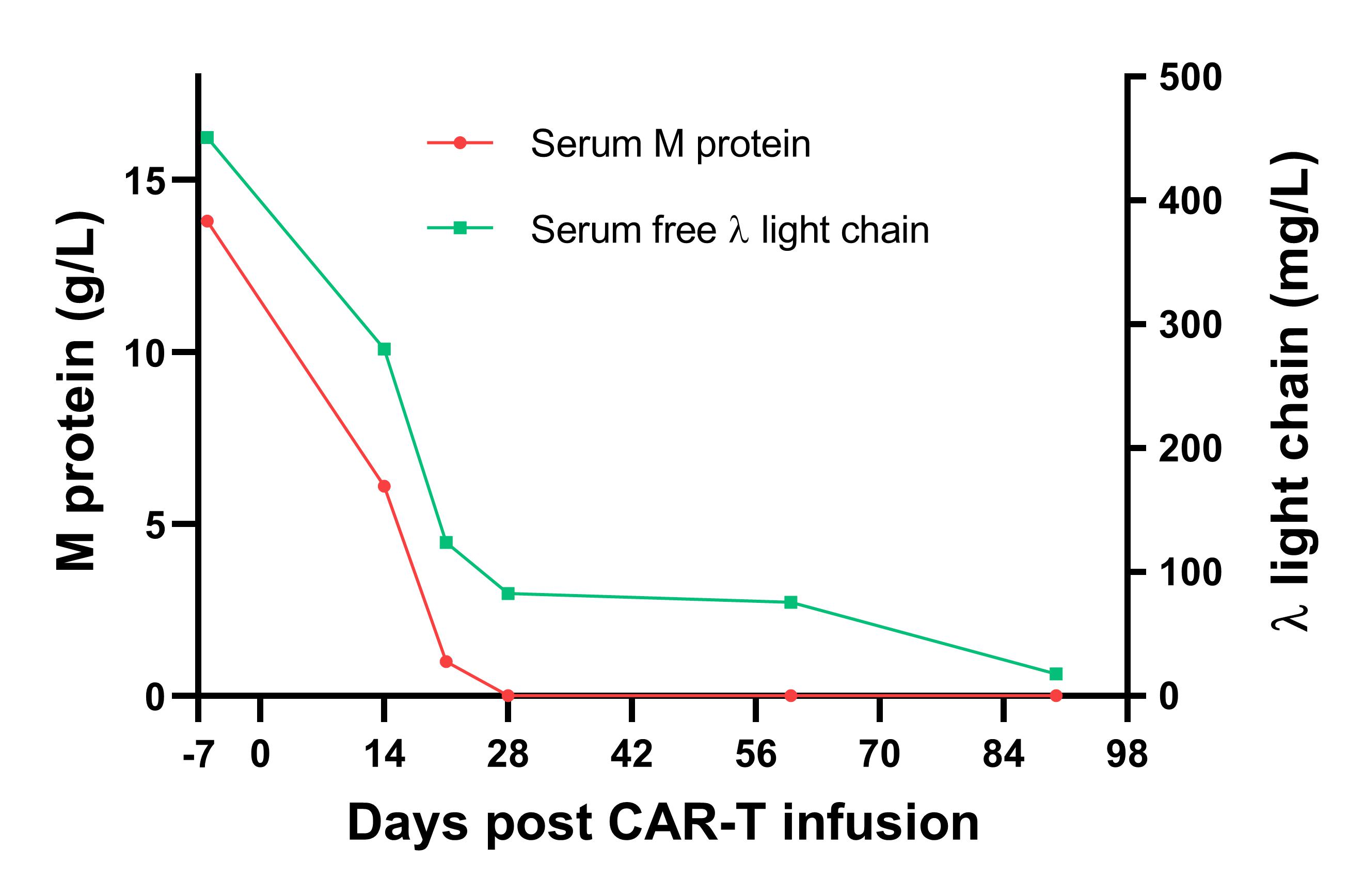

Supplement: Supplementary file 2 — Additional file 2. Fig. S2. Change of M protein and serum free light chain in patient 2 before and after treatment. [file 12967_2023_4655_MOESM2_ESM.jpg]
