# Supplementary material for: A novel two-step administration of XPO-1 inhibitor may enhance the effect of anti-BCMA CAR-T in relapsed/refractory extramedullary multiple myeloma
Source: J Transl Med. 2023 Nov 15;21:812. doi: 10.1186/s12967-023-04655-w (PMC10647128; doi:10.1186/s12967-023-04655-w)
Supplement: Supplementary file 3 — Additional file 3. Trial protocol. [file 12967_2023_4655_MOESM3_ESM.docx]

**PROTOCOL**

A Phase I Study of a Novel Fully human BCMA-Targeting CAR (CT103A) Combined with Selinexor in Patients with Relapsed/Refractory Extramedullary Multiple Myeloma

Protocol Number: XL-LCYJ-0003

Protocol Version: Version 1.0

Version Date: 2021-11-9

Principle Investigator: Dr. Chunrui Li

Study Site: Tongji Hospital of Tongji medical school, Huazhong University of Science and Technology

CAR-T Producer: Nanjing IASO Biotherapeutics Ltd.

Study Collaborator: Nanjing IASO Biotherapeutics Ltd. and Antengene Corporation Ltd.

# PROTOCOL SYNOPSIS

| **Title of Study** | A Phase I Study of a Novel Fully human BCMA-Targeting CAR (CT103A) Combined with Selinexor in Patients with relapsed/Refractory Extramedullary Multiple Myeloma |
| --- | --- |
| **Phase** | Phase I |
| **Methodology** | This is an open-able and single-center study to evaluate the efficacy and safety of CT103A in combination with different doses of Selinexor in patients with relapsed/refractory extramedullary multiple myeloma (EMM), as well as the pharmacokinetics (PK) and pharmacodynamics (PD) of Selinexor and CT103A. |
| **Study Duration** | The main follow-up period is one year after cell infusion. |
| **Study Site** | Tongji Hospital, Tongji Medical College, Huazhong University of Science & Technology |
| **Objectives** | **Primary objective**   - To evaluate the efficacy of CT103A infusion in combination with Selinexor in patients with relapsed/refractory EMM   **Secondary objectives**   - To evaluate the safety of CT103A infusion in combination with Selinexor in patients with relapsed/refractory EMM - To evaluate PK characteristics after administrations of CT103A and Selinexor - To evaluate PD characteristics after administrations of CT103A and Selinexor - To evaluate patients’ quality of life after administrations of CT103A and Selinexor - To evaluate the immune status of patients after administrations of CT103A and Selinexor   **Exploratory objective**   - To evaluate the impact of Selinexor on CT103A |
| **Investigational Drug, Dose, and Groups** | CT103A: a novel fully human anti-B cell maturation antigen (BCMA)-CAR autologous T cells injection  Selinexor: a selective inhibitor of nuclear export protein 1  There are two dose groups (20 mg/week and 40 mg/week) for Selinexor, the dose of CT103A in both groups is 1.0×10^6^ CAR^+^ cells/kg. Patients in both dose groups will receive a single CT103A cell infusion. After at least one month after CT103A infusion and the platelet count recovery to ≥50×10^9^/L, Selinexor will be started orally once a week for one year. |
| **Sample Size** | Eight to ten patients will be enrolled at each dose level, and 16 to 20 patients are expected to be enrolled. |
| **Inclusion Criteria** | Patients must meet all of the following criteria to be included in this study:   1. Patients must be ≥18 years of age, male or female. 2. Patients must have received at least three prior lines of therapy with relapsed, progressive, or refractory EMM, according to the International Myeloma Working Group (IMWG) Consensus on multiple myeloma (MM) (2016). 3. The tumor cells of patients must have positive BCMA expression, as determined by immunohistochemistry (IHC) or flow cytometry. 4. Measurable disease at Screening as defined by the following criteria:   Patients with extramedullary myeloma must have an extramedullary plasmacytoma at least 2 cm in maximum diameter; or have serous effusion in which monoclonal plasma cells are detectable.   1. Eastern Cooperative Oncology Group (ECOG) performance status of 0 to 2. 2. Have an expected survival of ≥12 weeks. 3. Patients must have adequate organ function and meet all of the following laboratory test results before enrollment:  - Complete blood count (CBC): absolute neutrophil count (ANC) ≥1 × 10^9^/L; absolute lymphocyte count (ALC) ≥0.3 × 10^9^/L; platelet count ≥50 × 10^9^/L; hemoglobin ≥60 g/L. - Liver function: alanine aminotransferase (ALT) and aspartate aminotransferase (AST) ≤2.5 × the upper limit of normal (ULN); serum total bilirubin (STB) ≤1.5 × ULN; - Renal function: creatinine clearance (CrCl) calculated using Cockcroft-Gault formula ≥ 40 mL/min (not applicable if the investigator determines that the abnormal renal function is caused by the progression of primary disease.   C$\mathrm{rC}l=\frac{\left( 140-age \right)\times Body weight\left( kg \right)}{72\times Serum creatine\left( \frac{mg}{dL} \right)}(\times0.85 F)$   - Coagulation function: fibrinogen ≥1.0 g/L; activated partial thromboplastin time (APTT) ≤1.5 × ULN, prothrombin time (PT) ≤1.5 × ULN. - Oxygen saturation >91%. - Left ventricular ejection fraction (LVEF) ≥50%.  1. Patients and their spouses agree to use reliable contraceptive methods, devices, or medicines (excluding the safe period contraception) within one year after CAR-T cell infusion. 2. Patients must sign the written informed consent form approved Ethics Committee by themselves before any screening procedures. |
| **Exclusion Criteria:** | Patients who meet any of the following criteria will be excluded:   1. Patients with known resistance to Selinexor. 2. Patients with graft-versus-host disease (GVHD) or autoimmune diseases requiring long-term immunosuppressive therapy. 3. Patients have received the following prior cancer therapies:  - Monoclonal antibody therapy for MM within 21 days before the monocyte collection, or - Cytotoxic chemotherapy or proteasome inhibitors within 14 days before the monocyte collection, or - Immunomodulatory agents within seven days before the monocyte collection, or - Other cancer therapy besides those listed above within 30 days before the monocyte collection.  1. Patients had received a therapeutic dose of corticosteroids (defined as prednisone or equivalent >20 mg/day) within seven days before screening, except for physiological replacement, topical, and inhaled steroids. 2. Patients with hypertension cannot be controlled by medication. 3. Severe heart disease: including but not limited to unstable angina, myocardial infarction (within six months before Screening), congestive heart failure (New York Heart Association [NYHA] functional classification ≥class III), serious cardiac arrhythmia. 4. Any unstable systemic disease as judged by the investigator: including but not limited to severe liver, renal, or metabolic disease requiring medication. 5. Malignant tumors other than MM within five years before the screening, excluding adequately treated carcinoma in situ of the cervix, basal cell or squamous cell skin cancer, localized prostate cancer following radical prostatectomy, and ductal carcinoma in situ after mastectomy. 6. Patients with a history of solid organ transplantation. 7. Have undergone major surgeries within two weeks before the monocyte collection or planned surgeries within two weeks after the study treatment (patients planning for local anesthesia may participate in this study). 8. Have received other interventional clinical trial medications within one month before signing the informed consent form. 9. Have any uncontrolled and active infection within seven days before the monocyte collection. 10. Have positive hepatitis B surface antigen (HBsAg), or positive hepatitis B core antibody (HBcAb) and detectable hepatitis B virus (HBV) DNA in the peripheral blood; positive hepatitis C virus (HCV) antibody and positive HCV RNA; positive human immunodeficiency virus (HIV) antibody; positive in cytomegalovirus DNA test; positive in syphilis test. 11. Women who are pregnant or breastfeeding. 12. Any mental illness, consciousness disorders, or central nervous system disease. 13. Other conditions inappropriate for enrollment in the judgment of the investigator. |
| **Study Design** | The study has been optimized and completed according to the principles and requirements of Good Clinical Practice (GCP) of China, Guidelines for the Clinical Trial and Evaluation of Cell Therapy Products (for Trial Implementation), Considerations for the Design of Clinical Trials of Chimeric Antigen Receptor T Lymphocytes for the Treatment of Malignancies of the Lymphohematopoietic System issued by CDE Biologics Clinical Department**,** and Guidelines for the Conditional Approval of Pharmaceuticals for Marketing (for Trial Implementation).  **Study Procedures:**  Study procedures include screening and enrollment, peripheral blood mononuclear cell (PBMC) collection, bridging therapy (Selinexor-based regimens), baseline assessments, lymphodepletion conditioning, rest assessment, CT103A cell infusion, administration of Selinexor, and the main follow-up period. |
| **Statistical Methods** | General Principles:  PK parameters will be calculated based on WinNonlin version 6.1 or above. All remaining statistical analyses will be performed based on SAS^®^ version 9.2 or above.  Unless otherwise specified, summary tables will be produced for the scheduled time points. Listings will be made by patient number, time points, and other variables for unscheduled time points.  Continuous variables, such as age, will be statistically described using the value of observations, mean, median, quartile, standard deviation, minimum, and maximum; categorical variables will be statistically described by the number of cases and the percentage in each category. For the PK parameters, such as the area under the curve (AUC) and maximum plasma concentration (C_max_), geometric mean and geometric coefficient of variation will be calculated. Time-event data will be estimated by the Kaplan-Meier method for the median time and 95% CIs for the population. The decimal places of the minimum and maximum values are consistent with the record on the eCRF; the number of decimal places for the mean and median values is 1 more than the original data; the number of decimal places for the standard deviation is 2 more than the original data, and the percentage is retained to 2 decimal places. The number of decimal places reserved for all statistics is generally not more than 4. Except for the patient distribution, statistical summary tables and charts will be based on the corresponding analysis set, and the relevant data of all patients will be listed.  Statistical analysis methods are detailed in the statistical analysis plan (SAP). |
| **CAR-T Producer** | Nanjing IASO Biotherapeutics Ltd. |

# TABLE OF CONTENTS

[PROTOCOL SYNOPSIS 2](#_Toc112064811)

[LIST OF ABBREVIATIONS 12](#_Toc112064813)

[1 INTRODUCTION 14](#_Toc112064814)

[1.1 Background on Disease 14](#_Toc112064815)

[1.2 Anti-BCMA CAR-T for Extramedullary Multiple Myeloma 15](#_Toc112064816)

[1.3 CT103A 15](#_Toc112064817)

[1.3.1 Clinical Evidence 16](#_Toc112064818)

[1.3.2 Adverse Events 16](#_Toc112064819)

[1.3.3 Pharmacokinetics and Pharmacodynamics in Human 17](#_Toc112064820)

[1.4 Selinexor 17](#_Toc112064821)

[1.4.1 Overview of Clinical Data of Selinexor 17](#_Toc112064822)

[1.4.2 Selinexor and T Cells Interactions 18](#_Toc112064823)

[1.4.3 Common Adverse Events of Selinexor 18](#_Toc112064824)

[1.5 CT103A in Combination With Selinexor 18](#_Toc112064825)

[2 STUDY OBJECTIVES AND ENDPOINTS 20](#_Toc112064826)

[2.1 Study Objectives 20](#_Toc112064827)

[2.1.1 Primary Objective 20](#_Toc112064828)

[2.1.2 Secondary Objectives 20](#_Toc112064829)

[2.1.3 Exploratory Objective 20](#_Toc112064830)

[2.2 Study Endpoints 20](#_Toc112064831)

[2.2.1 Primary Endpoint 20](#_Toc112064832)

[2.2.2 Secondary Endpoints 20](#_Toc112064833)

[2.2.3 Exploratory Endpoint 21](#_Toc112064834)

[3 INVESTIGATIONAL PLAN 22](#_Toc112064835)

[4 SELECTION OF STUDY POPULATION 23](#_Toc112064836)

[4.1 Inclusion Criteria 23](#_Toc112064837)

[4.2 Exclusion Criteria 24](#_Toc112064838)

[4.3 Study Termination and Withdrawal 25](#_Toc112064839)

[4.3.1 Study Termination/Suspension 25](#_Toc112064840)

[4.3.2 Withdrawal Criteria 25](#_Toc112064841)

[5 STUDY TREATMENTS 27](#_Toc112064842)

[5.1 Study Treatment Description 27](#_Toc112064843)

[5.1.1 Details of Study Treatment 27](#_Toc112064844)

[5.1.1.1 CT103A 27](#_Toc112064845)

[5.1.1.2 Selinexor 27](#_Toc112064846)

[5.1.2 Bridging Therapy 27](#_Toc112064847)

[5.1.3 Lymphodepletion 27](#_Toc112064848)

[5.1.4 Maintenance Therapy (Selinexor) 28](#_Toc112064849)

[5.2 Administration of CT103A 28](#_Toc112064850)

[5.2.1 Method of Administration 28](#_Toc112064851)

[5.2.2 Indications for Dose Adjustment and Withdrawal 29](#_Toc112064852)

[5.2.2.1 Dose Delays and Adjustments 29](#_Toc112064853)

[5.2.2.2 Criteria for Study Treatment Discontinuation 29](#_Toc112064854)

[5.2.2.3 Precautions 29](#_Toc112064855)

[5.3 Concomitant Therapies 30](#_Toc112064856)

[5.3.1 Prohibited Concomitant Therapies 31](#_Toc112064857)

[5.3.2 Permitted Concomitant Therapies 31](#_Toc112064858)

[5.4 Receipt and Return of CT103A 32](#_Toc112064859)

[6 STUDY PROCEDURES 33](#_Toc112064860)

[6.1 Screening and Enrollment Period (-8 w to-18 d) 43](#_Toc112064861)

[6.1.1 Screening Period 43](#_Toc112064862)

[6.1.2 Enrollment 46](#_Toc112064863)

[6.2 PBMC Collection (-8 w to -18 d) 46](#_Toc112064864)

[6.3 Baseline Assessments (within 7 d before lymphocyte clearance administration) 47](#_Toc112064865)

[6.4 Lymphodepleting Conditioning（-7 d to -2 d） 49](#_Toc112064866)

[6.5 Rest Assessment Before Treatment (-1 d) 49](#_Toc112064867)

[6.6 CT103A Cell Infusion (0 d) 50](#_Toc112064868)

[6.7 Main Follow-up Period (1 d to 1 year after Cell Infusion) 51](#_Toc112064869)

[6.7.1 Visits during inpatient observation and within 28 days after the cell infusion (1 d to 28 d) 51](#_Toc112064870)

[6.7.2 Selinexor Administration 52](#_Toc112064871)

[6.7.3 Subsequent Follow-up Visits 53](#_Toc112064872)

[6.8 Withdrawal from the study 55](#_Toc112064873)

[6.9 Unscheduled visits 57](#_Toc112064874)

[7 SAFETY ASSESSMENTS 58](#_Toc112064875)

[7.1 Adverse Event 58](#_Toc112064876)

[7.1.1 Definition of Adverse Events 58](#_Toc112064877)

[7.1.2 Serious Adverse Event 58](#_Toc112064878)

[7.1.3 Suspected Unexpected Serious Adverse Reactions 59](#_Toc112064879)

[7.1.4 Management, Collection and Recording of Adverse Events 60](#_Toc112064880)

[7.1.4.1 Management of Adverse Events 60](#_Toc112064881)

[7.1.4.2 Collection of Adverse Events 63](#_Toc112064882)

[7.1.4.3 Recording of Adverse Events 63](#_Toc112064883)

[7.1.5 Reporting of Serious Adverse Events 64](#_Toc112064884)

[7.1.6 Grading of Adverse Events 64](#_Toc112064885)

[7.1.7 Relationship to the study treatment 64](#_Toc112064886)

[7.1.8 Follow-up of Adverse Events/Serious Adverse Events 65](#_Toc112064887)

[7.1.9 Outcome of Adverse Events 65](#_Toc112064888)

[7.1.10 Disease Progression 66](#_Toc112064889)

[7.1.11 New Cancer 66](#_Toc112064890)

[7.2 ECOG Physical Capacity Score 66](#_Toc112064891)

[7.3 Laboratory Tests (for safety assessment) 66](#_Toc112064892)

[7.4 Vital Signs 67](#_Toc112064893)

[7.5 Physical Examination 67](#_Toc112064894)

[7.6 Electrocardiogram and Echocardiogram 67](#_Toc112064895)

[8 EFFICACY ASSESSMENTS 68](#_Toc112064896)

[8.1 Efficacy Evaluation Criteria 68](#_Toc112064897)

[8.2 Effective Assessment Indicators 68](#_Toc112064898)

[9 PHARMACOKINETICS 69](#_Toc112064899)

[9.1 Pharmacokinetics Evaluation Indicators 69](#_Toc112064900)

[9.2 Pharmacokinetic Blood Sample Test 69](#_Toc112064901)

[10 PHARMACODYNAMICS 70](#_Toc112064902)

[10.1 Pharmacodynamic Evaluation Indicators 70](#_Toc112064903)

[10.2 Pharmacodynamic blood sample Test 70](#_Toc112064904)

[11 Quality of Life Assessment 71](#_Toc112064905)

[12 Immune Status of the Patients 72](#_Toc112064906)

[13 STATISTICAL ANALYSIS 73](#_Toc112064907)

[13.1 General Methods 73](#_Toc112064908)

[13.2 Sample Size 73](#_Toc112064909)

[13.3 Analysis Populations 73](#_Toc112064910)

[13.4 Handling of Missing Values 74](#_Toc112064911)

[13.5 Statistical Methodology 74](#_Toc112064912)

[13.5.1 Patient Distribution 74](#_Toc112064913)

[13.5.2 Demographic Data and Baseline Analysis 75](#_Toc112064914)

[13.5.3 Analysis of Safety Measures 75](#_Toc112064915)

[13.5.4 Efficacy Analysis 76](#_Toc112064916)

[13.5.5 PK/PD Analysis 76](#_Toc112064917)

[13.5.6 Exploratory Analysis 76](#_Toc112064918)

[13.6 Summary and Analysis 76](#_Toc112064919)

[14 STUDY MANAGEMENT 77](#_Toc112064920)

[14.1 Data Handling and Record Keeping 77](#_Toc112064921)

[14.1.1 Data Collection 77](#_Toc112064922)

[14.1.2 Data Monitoring 77](#_Toc112064923)

[14.1.3 Data Entry and Modification 78](#_Toc112064924)

[14.1.4 Database Lock 78](#_Toc112064925)

[14.1.5 Data Transfer 78](#_Toc112064926)

[14.1.6 Data Preservation 78](#_Toc112064927)

[14.2 Ethical Considerations and Informed Consent 78](#_Toc112064928)

[14.3 Quality control 79](#_Toc112064929)

[14.3.1 Inspection 79](#_Toc112064930)

[14.3.2 Data Verification 79](#_Toc112064931)

[14.3.3 Audit and Inspection 80](#_Toc112064932)

[14.3.4 Personnel Training 80](#_Toc112064933)

[14.4 Protocol Approval and Amendments 80](#_Toc112064934)

[14.5 Duration of Clinical Study 80](#_Toc112064935)

[14.6 Confidential Data Protection 80](#_Toc112064936)

[14.7 Financing and Insurance 80](#_Toc112064937)

[15 REFERENCES 81](#_Toc112064938)

[16 APPENDICES 85](#_Toc112064939)

# LIST OF ABBREVIATIONS

| **Abbreviation** | **Definition** |
| --- | --- |
| AE | adverse event |
| APTT | activated partial thromboplastin time |
| AUC_0-28d_ | area under the curve from time zero to Day 28 |
| AUC_0-90d_ | area under the curve from time zero to Day 90 |
| BCMA | B cell maturation antigen |
| CT | computed tomography |
| CTCAE | Common Terminology Criteria for Adverse Reactions |
| C_max_ | maximum plasma concentration |
| CR | complete response |
| CRA | clinical research associate |
| CRES | cell relevant encephalopathy syndrome |
| CRS | cytokine release syndrome |
| DOR | duration of response |
| ECG | electrocardiogram |
| ECOG | Eastern Cooperative Oncology Group |
| eCRF | electronic case report form |
| EMM | extramedullary multiple myeloma |
| GCP | Good Clinical Practice |
| IEC | independent ethics committee |
| HBV | hepatitis B virus |
| HCV | hepatitis C virus |
| ICANS | immune effector cell-associated neurotoxicity syndrome |
| ICF | informed consent form |
| IL-6 | interleukin-6 |
| IMWG | International Myeloma Working Group |
| MM | multiple myeloma |
| MRD | minimal residual disease |
| MRI | magnetic resonance imaging |
| NCI | National Cancer Institute |
| ORR | objective response rate |
| OS | overall survival |
| PBMC | peripheral blood mononuclear cell |
| PD | pharmacodynamic(s) |
| PET | positron emission tomography |
| PFS | progression-free survival |
| PK | pharmacokinetic(s) |
| PR | partial response |
| PT | preferred term |
| RR/MM | relapsed/refractory multiple myeloma |
| SAE | serious adverse event |
| SAP | statistical analysis plan |
| sCR | stringent complete response |
| SOC | systematic organ classification |
| SOP | standard operating procedure |
| T_max_ | time to maximum plasma concentration |
| TRAE | treatment-related adverse event |
| ULN | upper limit of normal |
| VCN | vector copy number |
| VGPR | very good partial response |
| XPO1 | nuclear export protein 1 |

# INTRODUCTION

## Background on Disease

Multiple myeloma (MM) is the second most common hematologic malignant tumor, which frequently occurs in the elderly [1, 2]. Extramedullary multiple myeloma (EMM) is an aggressive subtype of MM that can involve multiple organs such as the central nervous system, liver, pleura, lymphatic system, and skin [3, 4]. EMM may present either at the initial diagnosis (primary EMM) or at the time of relapse (secondary EMM) in MM patients. Most of the studies suggest that EMM can be categorized as extramedullary extraosseous type (resulting from hematogenous spread) and extramedullary-bone related type (from bone to contiguous soft Tissues) [5, 6]. Extramedullary lesions are more commonly detected during the course of treatment than in the initial stage of the disease, which can be up to 34.2%[7]. In recent years, with the application of proteasome inhibitors, immunomodulators, autologous hematopoietic stem cell transplantation, and monoclonal antibodies, the overall survival (OS) of myeloma patients has been greatly prolonged. With the popularization of several highly sensitive detection methods, the detection rate of EMM further rises. Previous reports have shown that the incidence of extramedullary disease can be as high as 70% in autopsy [8], suggesting that extramedullary lesions may be a natural outcome of progression in myeloma patients.

As the survival of extramedullary tumor cells is not dependent on the bone marrow microenvironment, they are more often associated with adverse cytogenetic features than intramedullary tumor cells, resulting in a poor survival outcome for the patient. Several studies in China and abroad have suggested that extramedullary disease is a risk factor for MM patients [9-11]. To date, there are no prospective studies specifically targeting extramedullary myeloma, and no standard treatment regimen is recommended by guidelines, suggesting that treating extramedullary myeloma is extremely difficult. Results from several retrospective studies have shown that conventional chemotherapeutic agents and novel agents, alone or in combination, have limited efficacy in patients with EMM [5, 6, 12, 13]. For example, in a study of 357 patients with MM (24 with secondary EMM), the median progression-free survival (PFS) and OS of patients with secondary EMM were approximately two months and seven months, respectively [14]. A retrospective study of carfilzomib monotherapy or combination therapy for the treatment of relapsed/refractory multiple myeloma (RR/MM) showed that patients with extramedullary infiltration had a shorter duration of response than those without extramedullary infiltration (3.9 months and 9.3 months, respectively) [15]. PFS and OS in patients with EMM treated with a daratumumab-based regimen were only 69 and 198 days, respectively [16]. Another Phase I/II study of daratumumab monotherapy or combination therapy for relapsed and refractory myeloma showed that the overall response rate of patients with EMM was significantly lower than that of patients without EMM (16.7% versus 33.1%) [17]. Although autologous hematopoietic stem cell transplantation has been used in many sites to treat EMM patients, most studies have shown poor efficacy.

## Anti-BCMA CAR-T for Extramedullary Multiple Myeloma

The efficacy of anti-B cell maturation antigen (BCMA) CAR-T cell therapy is well‑established in patients with RR/MM. Still, the efficacy is unsatisfactory in patients with EMM [18-25]. The efficacy data of patients with EMM were summarized from 2 previous anti-BCMA CAR-T clinical trials, which were conducted by our center “A single-center, open-label, single-arm clinical study of the safety and efficacy of anti-BCMA CAR-T infusion (mouse-derived) in the treatment of relapsed/refractory/high-risk BCMA+ cell tumors” (Chinese Clinical Trial Registry No.: ChiCTR-OPC-16009113) and “A Phase I, single-center, open-label clinical study of the safety and efficacy of fully human anti-BCMA CAR-T (CT103A) cell infusion in the treatment of relapsed/refractory plasma cell tumors” (Chinese Clinical Trial Registry No.: ChiCTR1800018137).

Results showed that among the 38 patients who received mouse-derived anti-BCMA CAR‑T infusion, the objective response rate (ORR) and complete response rate (≥ CR) in the EMM group (n = 18) were lower than those in the non-EMM group (n = 20), although the difference was not statistically significant (ORR: 77.8% versus 90%, p = 0.395; ≥CR: 38.89% versus 45%, p = 0.752), OS in the EMM group was significantly shorter than that in the non-EMM group (248 days versus 640 days, p = 0.007); among the 25 patients who received fully human anti-BCMA CAR-T infusion, there was no significant difference in ORR between the EMM group (n = 7) and non-EMM group (n = 16) (100% versus 100%), but ≥CR in EMM group was significantly lower than that in the non-EMM group (28.57% versus 81.25%, p = 0.026), PFS in EMM group was significantly shorter than that in the non-EMM group (121 versus not reached, p = 0.005), median OS was not reached in both groups. However, the 1-year OS rates for patients in the EMM and non-EMM groups were 57.14% (4/7) and 75% (12/16), respectively (Details see the article published on Front Immunol. 2021 Oct 29). Similar results have been observed in the published literature and international conference reports on the efficacy of other anti-BCMA CAR-T products [26-32].

These results indicate that anti-BCMA CAR-T is significantly superior to existing medications in terms of response rate, depth of remission, and survival rate in EMM patients. However, the overall efficacy of EMM patients is still inferior to that of non-EMM patients. As the efficacy and safety of fully human anti-BCMA CAR-T used in our site are superior to that of the mouse-derived anti-BCMA CAR-T, fully human anti-BCMA CAR-T (CT103A) in combination with Selinexor will be used for the treatment of patients with extramedullary myeloma in this study.

## CT103A

CT103A is an individualized, BCMA-targeted, gene-modified autologous T-cell immunotherapy that can identify and eliminate malignant and normal cells expressing BCMA. CAR specifically recognizes BCMA as a single chain fragment variable (ScFv) of fully human origin with low immunogenicity, and promotes the activation, proliferation, cytokine secretion, and targeted cell killing of CAR-T through the CD3ζ domain, and enhances the amplification and persistence of CAR-T with a 4-1BB costimulatory signal. CT103A cells have a high affinity to BCMA antigen and high killing efficacy against antigen-loaded cells in vitro and in vivo.

### Clinical Evidence

The previous single-center, dose-escalation, single-arm, open-label, standard 3+3 design study, with cut-off at 30 June 2020, screened 37 patients with RR/MM. Treatment administration was completed in 23 patients (5 in the 0.5 × 10^6^ CAR-T/kg dose group, 9 in the 1.0 × 10^6^ CAR‑T/kg dose group, 6 in the 3.0 × 10^6^ CAR-T/kg dose group, and 3 in the 6.0 × 10^6^ CAR‑T/kg dose group). All 23 patients responded to treatment with a 3-month best ORR of 100% and a complete response rate of 52.2%. This portion of the study data has been published in the BLOOD journal (Blood. 2021 May 27;137(21):2890-2901. doi:10.1182/blood.2020008936).

### Adverse Events

**Cytokine Release Syndrome (CRS) and Immune effector cell-associated neurotoxicity syndrome (ICANS)**

In the above study, almost all patients (95.7%) experienced CRS of different severity after the CT103A infusion, and the severity of CRS showed a correlation with the dose of CT103A infused. No Grade ≥3 CRS was observed in the 0.5 × 10^6^ CAR-T/kg dose group. Grade 3 CRS was observed in the 1.0 × 10^6^ CAR-T/kg and 3.0 × 10^6^ CART/kg dose groups, with the incidence of 22.2% and 16.7%, respectively. A Grade 4 CRS was observed in the 6.0 × 10^6^ CAR-T/kg dose group. All CRS resolved after the treatment with tocilizumab, steroids, and symptomatic treatment. The use of tocilizumab and plasma exchange was lower in the 0.5 × 10^6^ CAR-T/kg and 1.0 × 10^6^ CAR-T/kg dose groups than in the higher dose group.

As of 30 June 2020, no study treatment-related ICANS had been reported.

**Other Common Adverse Events (AEs)**

In addition to CRS, other common treatment-related adverse events (TRAEs) typically include hematotoxicity (decreased blood cells), gastrointestinal reactions, coagulation abnormalities, decreased immunoglobulin, and electrolyte disorder. Most TRAEs resolved within three months with no apparent correlation between the incidence and the dose. The TRAEs lasting six months after CT103A infusion were mainly hematotoxicity and immunoglobulin reduction, and the incidence of TRAEs showed a certain dose correlation.

**Serious Adverse Events (SAEs)**

In this study, 15 patients experienced 24 SAEs, including Grade 1 pleurisy, Grade 3 herpes zoster, Grade 3 appendicitis, Grade 1 to 5 infectious pneumonia, Grade 3 to 5 shock, Grade 3 to 4 thrombocytopenia, Grade 4 hypoxemia, Grade 4 CRS, Grade 4 neutrophil count decreased, and Grade 5 hemorrhagic shock. Two patients in the 0.5 × 10^6^ CAR-T cells/kg dose group experienced Grade 4 thrombocytopenia, possibly related to the study treatment assessed by the investigator. One patient in the 6.0 × 10^6^ CAR-T cells/kg dose group experienced Grade 4 CRS, which was definitely related to the study treatment assessed by the investigator.

### Pharmacokinetics and Pharmacodynamics in Human

The results of vector copy number (VCN) pharmacokinetic (PK) parameter analysis showed that the PK of CT103A in patients were characterized by high variability, with individual differences being significant and the geometric mean variation of each PK parameter ranging from 92% to 4625% (except for the 6.0 × 10^6^ CAR-T cells/kg group where only 2 cases had relatively complete PK data). When comparing PK data from the three dose groups, the 1.0×10^6^ CAR-T/kg group had the longest geometric mean half-life (T1/2) of 39 days, the shortest median time to maximum plasma concentration (T_max_) of 12 days, the highest geometric mean maximum plasma concentration (C_max_) of 598 90 copies/μg DNA, and the highest geometric mean area under the curve (AUC) (area under the curve from time zero to Day 28 [AUC0-28d] 697 504.9 day*copies/μg DNA, area under the curve from time zero to Day 90 [AUC0-90d] 1 153 666.3 day*copies/μg DNA, area under the curve from time zero to infinity [AUC0-∞] 1640378.1 day*copies/μg DNA). The 1.0 × 10^6^ CAR-T cells/kg dose group had the optimal PK characteristics of the CAR-T treatment.

After the CT103A cell infusion, the serum-free BCMA (sBCMA) was significantly decreased in each dose group. The sBCMA level rapidly decreased to below normal levels (15 μg/mL) in most responding patients. Patients with increased sBCMA levels, if not higher than normal, were often accompanied by a shift in minimal residual disease (MRD) to positive and developed into disease progression after 6 to 90 days.

In conclusion, CT103A is safe and effective, with good amplification and persistence in clinical studies. The safety of the 1.0 × 10^6^ CART/kg dose group was slightly better than that of the 3.0 × 10^6^ CAR-T/kg dose group. However, the dose escalation did not lead to better clinical efficacy or increase in amplification level and duration in vivo and did not increase the clinical benefits of patients but reduced the long-term clinical safety. Therefore, 1.0 × 10^6^ CAR-T/kg was selected as the dose for this study.

## Selinexor

### Overview of Clinical Data of Selinexor

Selinexor, the oral first-in-class and only selective inhibitor of the nuclear export protein 1 (XPO1) [33], has been approved by the United States (US) Food and Drug Administration (FDA) and recommended by the National Comprehensive Cancer Network (NCCN), Chinese Society of Clinical Oncology (CSCO), and European Society for Medical Oncology (ESMO) Guidelines for the treatment of RR/MM. In China, Selinexor was approved by National Medical Products Administration (NMPA) in February 2021 through a priority review process for new drug marketing applications, and the indication is RR/MM. In April 2021, Selinexor had already issued its first prescription in mainland China at the Boao Super Hospital in Boao Lecheng Pilot Zone and had been allowed for outpatient use. In clinical studies, XPO1 inhibitor-based monotherapy or combination therapy with proteasome inhibitor, immunomodulatory agent, or daratumumab has significantly improved the prognosis of MM patients with resistance to multiple drugs [34-36]. It has been reported that 16 patients with triple-class penta-exposed refractory extramedullary myeloma were treated with Selinexor (80 mg twice a week) in combination with dexamethasone. Nine of them had plasmacytomas that completely disappeared or showed the reduced size and/or metabolic activity, indicating that Selinexor remained effective in EMM patients receiving multiline therapy and even in patients with resistant EMM.

### Selinexor and T Cells Interactions

As the nuclear export protein is necessary for the development and function of T cells, foreign studies have shown that high concentrations of XPO1 inhibitors can affect the function of CD8+ T cells. However, the effect on its function could be eliminated by reducing the dose concentration (below 100 nM) or extending the dosing interval, thereby maintaining normal immune homeostasis [36]. Data from the previous study showed that Selinexor had no significant effect on the proliferation or apoptosis of CAR-T cells within the range of 500 nM.

### Common Adverse Events of Selinexor

Patients enrolled in the STORM study were triple-class refractory after being penta-exposed to bortezomib, carfilzomib, lenalidomide, pomalidomide, and daratumumab, with no significant organ toxicity or cumulative toxicity during the study. The most common Grade 1/2 AEs included gastrointestinal and systemic AEs; the most common Grade ≥ 3 AEs were mainly hematological adverse reactions, including neutropenia, thrombocytopenia, anemia, and leukopenia. The AEs could be effectively managed by supportive care and dose adjustments.

## CT103A in Combination with Selinexor

This study will enroll patients with RR/MM with EMM who have received at least three prior lines of therapy and have relapsed, progressed, or are refractory. This patient population has a low response rate and short median survival time. Many guidelines of MM in China and abroad suggest priority to include such population to participate in clinical studies. This study will use CT103A, a fully human BCMA-targeted CAR-T cell product, in combination with Selinexor to treat these patients and kill tumor cells through various mechanisms of action to improve the clinical efficacy of RR/MM patients with EMM. Based on the results of previous studies, after one month of anti-BCMA CAR-T cell infusion and recovery of platelet count, the combination therapy with Selinexor (20 mg/week or 40 mg/week, once a week) is selected, which is less than the conventionally recommended dose of Selinexor, so as to avoid potential adverse reactions.

The product used in this study, Selinexor, has been approved by the US FDA for patients with RR/MM. As the team leader of Phase I/II clinical study of CT103A, the safety and efficacy of CT103A have been fully verified. In addition, our site has extensive experience in clinical studies of CAR-T, by weighing the benefit/risk ratio of patients participating in the clinical studies, it can be concluded that the clinical benefits of patients outweigh the potential risks, and participation in this study would be beneficial to patients.

# STUDY OBJECTIVES AND ENDPOINTS

## Study Objectives

### Primary Objective

- To evaluate the efficacy of CT103A infusion in combination with Selinexor in patients with relapsed/refractory EMM.

### Secondary Objectives

- To evaluate the safety of CT103A infusion in combination with Selinexor in patients with relapsed/refractory EMM.
- To evaluate the PK characteristics after administrations of CT103A and Selinexor.
- To evaluate the pharmacodynamic (PD) characteristics after administrations of CT103A and Selinexor.
- To evaluate the patients’ quality of life after administrations of CT103A and Selinexor.
- To evaluate the immune status of patients after administrations of CT103A and Selinexor.

### Exploratory Objective

- To explore the effect of Selinexor on CT103A.

## Study Endpoints

### Primary Endpoint

Primary efficacy endpoint (according to International Myeloma Working Group [IMWG] criteria):

- PFS: the time from the CT103A infusion to disease progression or death from any cause.
- ORR: the proportion of patients who achieved stringent complete response (sCR), complete response (CR), very good partial response (VGPR), or partial response (PR).
- Duration of response (DOR): the time from the first evaluation of PR or, better to, the initial assessment of disease progression or death from any cause.

### Secondary Endpoints

- Secondary efficacy endpoints (according to IMWG criteria):
- OS: the time from CT103A infusion to death from any cause.
- MRD response assessment: the MRD assessment includes the proportion of MRD negative and the duration of MRD negative.
- Safety endpoints: AEs, laboratory tests, vital signs, physical examinations, etc.
- PK endpoints: C_max_, T_max_, AUC_0-28d_, and AUC_0-90d_ of CT103A cells and lentiviral VCN increased in peripheral blood after administration; Selinexor concentration in peripheral blood.
- PD endpoints: concentrations of CAR-T-associated inflammatory factors, such as interleukin-6 (IL-6) and ferritin at each time point.
- Health-related quality of life assessment: using the European Organization for Research and Treatment of Cancer Quality of Life Questionnaire-C30 (EORTC-QLQ-C30).
- Quality of life assessment: using the European Organization for Research and Treatment of Cancer Quality of Life Questionnaire Multiple Myeloma Module (EORTC-QLQ-MY20).
- Assessment of patient immune status: including changes in lymphocyte subsets, immunoglobulins, as well as infectious status.

### Exploratory Endpoint

- The effect of different doses of Selinexor on the efficacy and mechanism of CT103A.

# INVESTIGATIONAL PLAN

This is an open-label and single-center study to evaluate the efficacy and safety of CT103A in combination with different doses of Selinexor in patients with relapsed/refractory EMM and the PK and PD of Selinexor and CT103A. There are two dose groups (20 mg/week and 40 mg/week) for Selinexor, the dose of CT103A in both groups is 1.0×10^6^ cells/kg. Patients in both dose groups will receive a single CT103A cell infusion. After at least one month of CT103A infusion and the platelets count recovery to ≥50 × 10^9^/L, Selinexor will be started orally once a week for one year. Eight to ten patients will be enrolled at each dose level, and 16 to 20 patients are expected to be enrolled.

An overview of the study design is shown in Figure 1.

Figure 1. Study Procedures

**
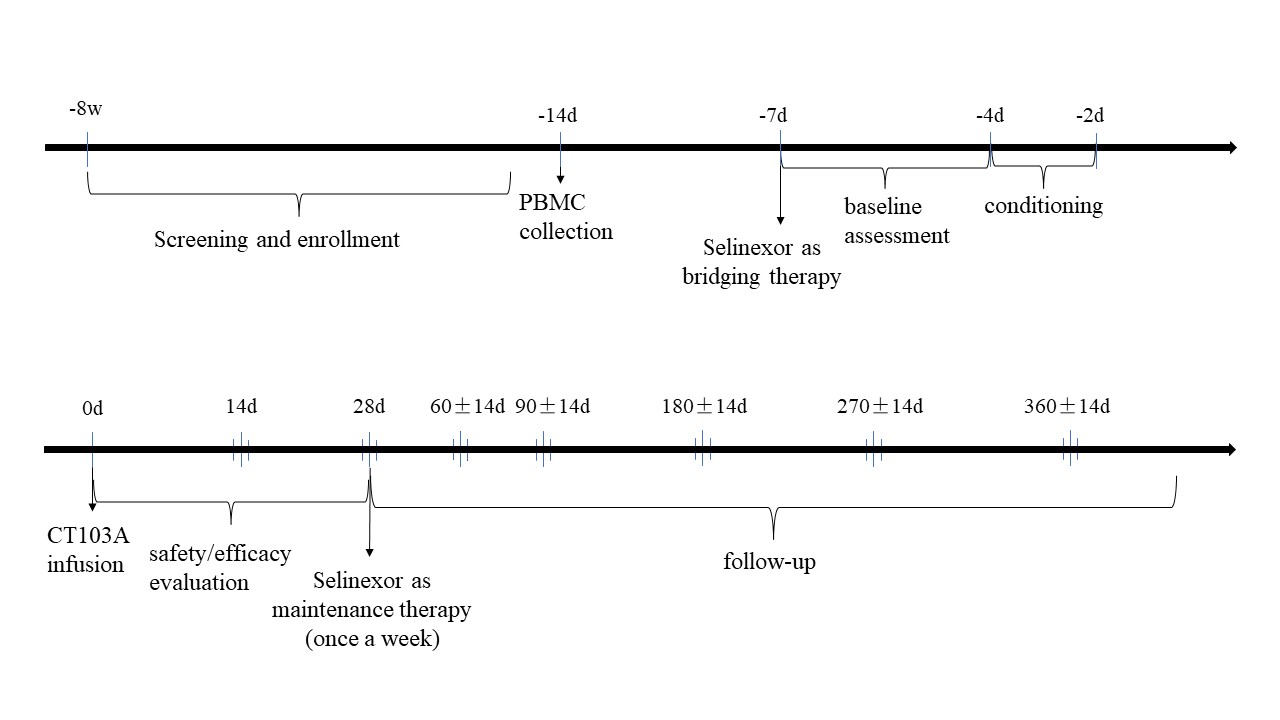
**

Notes: The study has been optimized and completed according to the principles and requirements of Good Clinical Practice (GCP) of China, Guidelines for the Clinical Trial and Evaluation of Cell Therapy Products (for Trial Implementation), Considerations for the Design of Clinical Trials of Chimeric Antigen Receptor T Lymphocytes for the Treatment of Malignancies of the Lymphohematopoietic System issued by CDE Biologics Clinical Department**,** and Guidelines for the Conditional Approval of Pharmaceuticals for Marketing (for Trial Implementation).

Study procedures include screening and enrollment, peripheral blood mononuclear cell (PBMC) collection, bridging therapy (Selinexor-based regimens）, baseline assessments, lymphodepletion conditioning, rest assessment, CT103A cell infusion, administration of Selinexor, and the main follow-up period.

# SELECTION OF STUDY POPULATION

## Inclusion Criteria

Patients must meet all of the following criteria to be included in this study:

1. Patients must be ≥18 years of age, male or female.
2. Patients must have received at least three prior lines of therapy with relapsed, progressive or refractory EMM, according to the IMWG Consensus on MM (2016).
3. The tumor cells of patients must have positive BCMA expression, as determined by immunohistochemistry (IHC) or flow cytometry.
4. Measurable disease at Screening as defined by the following criteria:

- Patients with extramedullary myeloma must have an extramedullary plasmacytoma at least ≥ 2 cm in maximum diameter or have serous effusion in which monoclonal plasma cells are detectable.

1. Eastern Cooperative Oncology Group (ECOG) performance status of 0 to 2 (see Appendix 1 for ECOG performance status score).
2. Have an expected survival of ≥12 weeks.
3. Patients must have adequate organ function and meet all of the following laboratory test results before enrollment:

- Complete blood count (CBC): absolute neutrophil count (ANC) ≥ 1 × 10^9^/L; absolute lymphocyte count (ALC) ≥0.3 × 10^9^/L; platelet count ≥50 × 10^9^/L; hemoglobin ≥ 60 g/L.
- Liver function: alanine aminotransferase (ALT) and aspartate aminotransferase (AST) ≤ 2.5 × the upper limit of normal (ULN); serum total bilirubin (STB) ≤ 1.5 × ULN.
- Renal function: creatinine clearance (CrCl) calculated using Cockcroft-Gault formula ≥40 mL/min (not applicable if the investigator determines that the abnormal renal function is caused by the progression of the primary disease.

C$\mathrm{rC}l=\frac{\left( 140-age \right)\times Body weight\left( kg \right)}{72\times Serum creatine\left( \frac{mg}{dL} \right)}(\times0.85 F)$

- Coagulation function: fibrinogen ≥ 1.0 g/L; activated partial thromboplastin time (APTT) ≤ 1.5 × ULN, prothrombin time (PT) ≤ 1.5 × ULN.
- Oxygen saturation >91%.
- Left ventricular ejection fraction (LVEF) ≥50%.

1. Patients and their spouses agree to use reliable contraceptive methods, devices, or medicines (excluding the safe period contraception) within one year after CAR-T cell infusion.
2. Patients must sign the written informed consent form approved Ethics Committee themselves before any screening procedures.

## Exclusion Criteria

Patients who meet any of the following criteria will be excluded:

1. Patients with known resistance to Selinexor.
2. Patients with graft-versus-host disease (GVHD) or autoimmune diseases requiring long-term immunosuppressive therapy.
3. Patients have received the following prior cancer therapies:

- Monoclonal antibody therapy for MM within 21 days before the monocyte collection, or
- Cytotoxic chemotherapy or proteasome inhibitors within 14 days before the monocyte collection, or
- Immunomodulatory agents within seven days before the monocyte collection, or
- Other cancer therapy other than those listed above within 30 days before the monocyte collection.

1. Patients had received a therapeutic dose of corticosteroids (defined as prednisone or equivalent > 20 mg/day) within seven days before screening, apart from physiological replacement, topical, and inhaled steroids.
2. Patients with hypertension cannot be controlled by medication.
3. Severe heart disease: including but not limited to unstable angina, myocardial infarction (within six months before screening), congestive heart failure (New York Heart Association [NYHA] functional classification ≥ class III), serious cardiac arrhythmia.
4. Any unstable systemic disease as judged by the investigator: including but not limited to severe liver, renal, or metabolic disease requiring medication.
5. Malignant tumors other than MM within five years before Screening, excluding adequately treated carcinoma in situ of the cervix, basal cell or squamous cell skin cancer, localized prostate cancer following radical prostatectomy, and ductal carcinoma in situ after mastectomy.
6. Patients with a history of solid organ transplantation.
7. Have undergone major surgeries within two weeks before the monocyte collection or planned surgeries within two weeks after the study treatment (patients planning for local anesthesia may participate in this study).
8. Have received other interventional clinical trial medications within one month before signing the informed consent form.
9. Have any uncontrolled and active infection within seven days before the monocyte collection.
10. Have positive hepatitis B surface antigen (HbsAg), or positive hepatitis B core antibody (HbcAb) and detectable hepatitis B virus (HBV) DNA in the peripheral blood; positive hepatitis C virus (HCV) antibody and positive HCV RNA; positive human immunodeficiency virus (HIV) antibody; positive in cytomegalovirus DNA test; positive in syphilis test.
11. Women who are pregnant or breastfeeding.
12. Any mental illness, consciousness disorders, or central nervous system disease.
13. Other conditions inappropriate for enrollment in the judgment of the investigator.

## Study Termination and Withdrawal

### Study Termination/Suspension

The study may be stopped or suspended if the following condition occurs:

**The investigator can stop/suspend the study.**

Before stopping/suspending a study, the investigator must inform the sponsor, ethics committee, institution, and relevant regulatory authorities and clarify the reasons. After the study termination/suspension, the re-initiation of the study must be approved by the ethics committee.

**Termination/suspension required by the Ethics Committee.**

Reasons for study termination/suspension include but not limited to the following:

- The number and severity of AEs in this study suggest that continuing the study would cause significant harm to the patients.
- Existing clinical data are of poor quality to continue this study.
- The study may be terminated due to changes in regulations and policies of national authorities.

### Withdrawal Criteria

Withdrawal means that patients who are lost to follow-up in this study due to disease progression, initiation of new cancer therapies, or other reasons.

All patients can withdraw their informed consent from the study at any time without prejudice or penalty. The investigator should collect the reasons and time for premature discontinuation, perform appropriate observations and evaluations, and complete the withdrawal visit/end-of-study visit as specified in the protocol.

Patients who withdraw from the study with treatment-related AEs should be followed up until the AE returns to the baseline or stabilizes. AEs may not be followed if further improvement is not expected in the investigator's judgment. For such cases, the investigator should maintain a record of their opinion in the source documentation for patients.

The investigator should make every effort to contact patients who have received treatment but failed to complete the study, or in the event that a patient fails to complete the follow-up visits, the contact details should be documented throughout the study (e.g., the time and date of telephone contact, and the evidence of letter delivery, etc.).

The reasons for premature discontinuation must be recorded on the source document and the electronic case report form (eCRF).

# STUDY TREATMENTS

## Study Treatment Description

### Details of Study Treatment

#### CT103A

**Treatment name:** fully human BCMA CAR-T Cell Injection (CT103A)

**Dosage:** 1.0×10^6^ CAR-T/kg

**Active ingredient:** fully human BCMA CAR-T Cell Injection

**Excipients:** compound electrolyte injection, human serum albumin, glucose injection, vitamin C injection, compound amino acid injection (18AA-II), dimethyl sulfoxide (DMSO)

**Package:** 1.0×10^6^ CAR-T/kg, 20 mL

**Appearance:** white or yellowish frozen solid, yellowish suspension liquid after thawed

**Storage and transportation conditions:** store in a gas-phase liquid nitrogen container (＜−130 ℃); transport in a liquid nitrogen transport container (＜−130 ℃)

**Manufacturer**: Nanjing IASO Biotherapeutics Ltd.

#### Selinexor

**Treatment name:** Selinexor

**Brand name:** Xpovio

**Dosage:** once a week, with different dosage groups: 20 mg/week and 40 mg/week.

**Storage:** store at or below 30 ℃.

**Manufacturer:** Karyopharm

### Bridging Therapy

Bridging therapy may be used at the discretion of the investigator following monocyte collection and be ended seven days before lymphodepleting conditioning to maintain the patients in a stable status. Selinexor-based regimens will be used as bridging therapy. Baseline assessments will be performed at least seven days after bridging therapy. Patients who achieve complete remission after the bridging therapy are not allowed to receive the lymphodepleting conditioning regimen.

### Lymphodepletion

The recommended lymphodepleting conditioning regimen is as follows:

Cyclophosphamide, 500 mg/m² intravenous infusion for more than 30 minutes, once daily for three consecutive days.

Fludarabine, 30 mg/m² intravenous infusion for more than 30 minutes, once daily for three consecutive days.

If the patient has impaired renal function or other conditions requiring dose adjustment for lymphodepleting chemotherapy, the investigator may adjust the dose after a comprehensive evaluation.

### Maintenance Therapy (Selinexor)

There are two dose groups in this study. Patients in the 20 mg dose group will receive oral Selinexor 20 mg/tablet × 1 tablet weekly. Patients in the 40 mg dose group will receive oral Selinexor 20 mg/tablet × 2 tablets weekly.

Patients enrolled in this study will be sequentially enrolled in the two dose groups. In the 40 mg/week dose group, if Selinexor-related AEs occur during the treatment, the dose could be reduced to 20 mg/week after the investigator’s assessment, and the dose could be increased to 40 mg/week after the condition returns to the baseline or stabilizes for ≥ 4 weeks.

## Administration of CT103A

### Method of Administration

Following lymphodepleting conditioning within one week before CT103A cell infusion, patients eligible for the treatment, as judged by the investigator upon assessment, will be given CT103A cell infusion.

**Premedication:** Patients are to be pre-medicated with 25 mg promethazine or 20 mg diphenhydramine hydrochloride intramuscularly 15 to 30 minutes before the infusion of CT103A cells. Acetaminophen or other nonsteroidal anti-inflammatory medications could be administered in the event of a fever (not caused by infection). Given that glucocorticoids may affect the survival of T cells, systemic glucocorticoids, such as hydrocortisone, prednisone, methylprednisolone, or dexamethasone, are not recommended except in life-threatening emergencies.

**Transportation of CT103A product:** CT103A cells are stored in gas-phase liquid nitrogen and transported below −130 ℃ to clinical sites.

**CT103A product resuscitation:** The resuscitation will be conducted according to standard operating procedure (SOP) or the manual of the study treatment.

If the package is damaged, leaky, or has signs of damage, the infusion is prohibited.

**Infusion of CT103A product:** Infusion should be performed as per SOP for infusion of the study treatment.

Emergency facilities should be provided during infusion to allow for prompt management of severe allergic reactions, severe hypotension, or other reactions. Vital signs (axillary temperature, respiration rate, heart rate, and blood pressure) will be measured at 15 (±3) minutes before the infusion, at the start of the infusion, and within 1 hour after the beginning of the infusion (every 15 [±3) minutes). If abnormal, vital signs will continue to be monitored as clinically indicated until stable.

### Indications for Dose Adjustment and Withdrawal

#### Dose Delays and Adjustments

Patients need to be evaluated for lymphodepleting conditioning before this procedure. Only for those patients who meet the criteria as judged by the investigator can lymphodepleting conditioning be performed. If the criteria for lymphodepletion are not met, the procedure may be postponed until the patient meets the criteria, which should not exceed eight weeks.

Before the infusion, patients need to rest and receive a second assessment for infusion. Those who meet the criteria for cell infusion, as judged by the investigator, will receive CT103A cell infusion. If the criteria for cell infusion are not met, a delay of up to 2 weeks is allowed. If longer than two weeks, the investigator will assess the need for subjects to re-initiate baseline measurements and lymphodepleting conditioning procedure.

No dose adjustment of CT103A is allowed, and a dose range of 30% is acceptable. To maximize the benefits to patients, a conditional infusion can be performed if the criteria for infusion are met when the number of CAR-T cells produced does not reach the administered dose of CT103A and when the maximum infusion dose is ≥ 0.5×10^6^ CAR-T/kg after the investigator fully assesses the safety of the study treatment and the patient’s condition.

#### Criteria for Study Treatment Discontinuation

In the event of a Grade 4 infusion-related or allergic reaction, the patient should immediately discontinue the infusion and receive symptomatic treatment, with the investigator evaluating the need for withdrawal from the study treatment.

#### Precautions

1. The CT103A product is an autologous cell therapy product. Before infusion, two investigators are required to check the identification information with the patients separately.
2. The CT103A product should be given in a clinical environment.
3. The water bath for CT103A product resuscitation should use clean tap water or autoclaved sterile water.
4. Before the infusion, the name and identification of the drug should be confirmed. It can only be used before the validity period, with integrity and sealed condition.
5. The appearance of the CT103A product is white or slightly yellow frozen solid, and it is a slightly yellow suspended liquid after thawing. Before intravenous use, the injection should be observed for precipitation, floccules, or crystallization. If any of the above is observed, the infusion should be suspended for the patient. At the same time, the sponsor should be informed of the abnormal situation, which should be dealt with according to the sponsor’s suggestion.
6. The CT103A product should be cryopreserved in liquid nitrogen and transported in gas-phase liquid nitrogen below −130℃ to each site. In case of any abnormal temperature during storage or transport, the drug shall be transferred to the specified temperature as soon as possible and suspended for patients; meanwhile, the sponsor should be timely informed of the abnormal temperature range and duration, which should be dealt with according to the sponsor’s suggestion.
7. The CT103A product should be infused with a disposable blood transfusion device.
8. The CT103A product should be infused immediately after resuscitation. If the infusion cannot be performed within 2 hours due to the patient or other reasons, it should be returned to the sponsor for destruction according to SOP.
9. Because the CT103A product is gene-modified autologous T cells with anti-human BCMA CAR structure on its surface, it may cause allergic reactions when administered. Anti-allergic agents should be available anytime (systemic glucocorticoids are not recommended except in life-threatening emergencies).
10. Hypotension may occur during the infusion. Therefore, it is recommended that antihypertensive agents should be discontinued 12 hours before and during the infusion.
11. The CT103A product may cause arrhythmia, so patients with a history of heart disease should be closely monitored.
12. During the study period, blood cell count and platelet count should be monitored regularly, and patients with cytopenia should be monitored closely.
13. The CT103A product cannot be used if beyond the expiry date.

## Concomitant Therapies

Concomitant medications and therapies refer to the simultaneous administration of 2 or more medications/treatments, which are defined as other medications (including chemicals, biologicals, Chinese patent medicines, etc.) or therapies to be accepted in addition to the treatment drugs in the study.

Concomitant therapies are collected from the time of signing the informed consent form (ICF) until one year after the CT103A cell infusion (if PFS ≥12 months) or at the out-of-study visit (12 months > PFS ≥ 3 months), whichever occurs first. If disease progression occurs within three months after the CT103A cell infusion, every effort will be made to collect information from the patient as much as possible up to three months after the cell infusion. All concomitant medications (including permitted and impermissible concomitant medications) and concomitant therapies should be recorded in the source document and on the eCRF. The following information should be recorded: drug/non-drug name, the reason for administration, dosage, strength, frequency, route, start date, and end date. Concomitant non-drug therapies should include name of the non-drug therapy, a description of the therapy, reason for treatment, start date, and end date for treatment. If the reason for the use of concomitant medications and therapies meets the definition of an AE, relevant information must also be recorded in the source document of this patient and on the eCRF.

### Prohibited Concomitant Therapies

- Other anti-tumor treatments: In addition to the study drugs specified in this trial, patients are not allowed to receive any other cancer therapy (except for bridging chemotherapy) simultaneously throughout the study, including cytotoxic agents, biological therapies, immunosuppressive agents, or any other investigational products.
- Live virus vaccines for immunization.
- Other therapies may affect study assessments at the discretion of the investigator.

### Permitted Concomitant Therapies

- The investigator is allowed to perform bridging therapy after apheresis for patients with high tumor burden or rapid progression during the screening period, but bridging therapy must be completed one week before the baseline measurements.
- Standard of care and palliative care for pre-existing conditions, medical and/or surgical complications, with records of all medications.
- Medications related to the prevention of infusion reactions are allowed. The investigator is allowed to use concomitant medications for AEs.
- If significant neutropenia or febrile neutropenia/infection is observed, prophylactic use of appropriate growth factors (e.g., granulocyte colony-stimulating factor [G-CSF]) may be administered by the investigator according to the American Society of Clinical Oncology (ASCO) guidelines.
- Others: antiemetics for prevention, medications for protecting gastric mucosa, routine medications for hypertension, and others that can be used as concomitant medications at the discretion of the investigator.

## Receipt and Return of CT103A

CT103A cells should be stored in liquid nitrogen, protected from shaking, and transported in gas‑phase liquid nitrogen below −130 ℃ to each site. Upon receipt of the CT103A product, the study site should verify the temperature at which the CT103A product is transported, inventory of the drug, and sign after verification is completed. The relevant records should be maintained at the study site. The drug storage temperature should be recorded daily, along with the inventory sheet. The following items should be recorded: the date of receipt, the quantity, the batch number, the issued quantity, and the remaining quantity in the inventory.

Only authorized personnel can access the CT103A product. The CT103A product should be infused immediately after the resuscitation using a water bath. If the intravenous drip cannot be performed immediately due to the patient or any other reasons for more than 2 hours, the CT103A product should be returned to the sponsor for destruction

After the infusion, the package should be destroyed as medical waste by the investigators, according to SOP, or returned to the sponsor for disposal.

# STUDY PROCEDURES

Study procedures include screening and enrollment, peripheral blood mononuclear cell (PBMC) collection, bridging therapy (Selinexor-based regimens), baseline assessments, lymphodepletion conditioning, rest assessment, CT103A cell infusion, administration of Selinexor, and the main follow-up period.

- Screening and enrollment period: -8 weeks to -18 days before CT103A infusion. Patients should complete the visits according to the schedule of assessments during the screening period. Patients who meet the enrollment criteria will be allocated a patient number after the investigators' assessment.
- PBMC collection: 1 day
- The healthy mononuclear cells of the patient will be extracted from the peripheral blood and then sent to Nanjing IASO Biotherapeutics Ltd. for CT103A cell preparation (at least 18 days from the arrival of PBMC to the company to the Certificate of Analysis issued, slightly delayed if due to holidays)
- Apheresis is performed according to the standard practice of the institution. The goal is to deliver it to the preparation site with a minimum of 1 × 10^9^ PBMCs in an apheresis sample (to ensure the success rate of cell product preparation, it is suggested to collect more than 2 × 10^9^ PBMCs), two times of apheresis may be arranged to achieve the target amount.
- If disease progression occurs after PBMC collection, the investigator will decide whether to provide bridging therapy.
- Bridging therapy: Bridging therapy may be used at the discretion of the investigator following monocyte collection and be ended seven days before lymphodepleting conditioning to maintain the patients in a stable status. Selinexor will be used as bridging therapy. Baseline assessments will be performed at least seven days after bridging therapy. Patients who achieve complete remission after the bridging therapy are not allowed to receive the lymphodepleting conditioning regimen.
- Baseline assessments:
- The baseline assessments will be completed within seven days before the lymphodepletion conditioning (vital signs and physical examination will be conducted within one day before the lymphodepletion conditioning). Lymphodepletion conditioning can be conducted when the results of each test meet the criteria of lymphodepletion treatment.
- Visit of lymphocyte clearance pretreatment: −7 days to −2 days before CT103A infusion

The recommended lymphodepleting conditioning regimen is as follows:

Cyclophosphamide, 500 mg/m² intravenous infusion for more than 30 minutes, once daily for three consecutive days.

Fludarabine, 30 mg/m² intravenous infusion for more than 30 minutes, once daily for three consecutive days.

If the patient has impaired renal function or other conditions requiring dose adjustment for lymphodepleting chemotherapy, the investigator may adjust the dose after a comprehensive evaluation.

- Rest assessment: day -1

If the status of the patient does not meet the standard of infusion after rest assessment, cell infusion cannot be performed. The investigators will provide symptomatic treatment until the patient is in a state that allows for cell infusion. The delayed infusion should not exceed two weeks.

- CT103A cell infusion: The day of CT103A cell infusion is recorded as Day 0. The dosage is 1 × 10^6^ cells/kg, with a single infusion by peripheral intravenous administration.
- Selinexor: There are two dose groups in this study. Patients in the 20 mg dose group will receive oral Selinexor 20 mg/tablet × 1 tablet weekly. Patients in the 40 mg dose group will receive oral Selinexor 20 mg/tablet × 2 tablets weekly.
- Patients enrolled in this study will be sequentially enrolled in the two dose groups. In the 40 mg/week dose group, if Selinexor-related AEs occur during the treatment, the dose could be reduced to 20 mg/week after the investigator’s assessment, and the dose could be increased to 40 mg/week after the condition returns to the baseline or stabilizes for ≥4 weeks.
- The main follow-up period: day 1 to 1 year after CT103A cell infusion.

The inpatient observation period should be at least 14 days post-infusion. The inpatient observation from 15 to 28 days should be in compliance with the local practice. The subsequent follow-up visits will be conducted once a month for the first three months and once every three months thereafter, until disease progression, withdrawal of informed consent, death, or termination of the study.

Table 1. Schedule of Assessments

| **Visits** | | **Screening and Enrollment1** | **PBMC Collection 2** | **Baseline Examination 3** | **Preconditioning of lymphocyte clearance 3** | **Rest Assessment** | **Cells Infusion 4** | **Main Follow-up Period 5** | | | | | | | | | |
| --- | --- | --- | --- | --- | --- | --- | --- | --- | --- | --- | --- | --- | --- | --- | --- | --- | --- |
|  |  |  |  |  |  |  |  | **Hospitalization Observation Period** | | | | |  | | | | |
|  |  | **Day -56~ -18** | | **Examination within 7 days prior to lymphodepletion** | **Day -7~ -2** | **Day -1** | **Day 0** | **D**  **1** | **D**  **4** | **D**  **7** | **D**  **10** | **D**  **14** | **D**  **21** | **D**  **28** | **D 60** | **D**  **90** | **Once/3 months (90 d)** |
| **Window** | | **×** |  |  |  |  |  |  |  | **±1d** | **±1d** | **±1d** | **±1d** | **±2d** | **±14d** | **±14d** | **±14d** |
| Informed Consent | | **×** |  |  |  |  |  |  |  |  |  |  |  |  |  |  |  |
| Inclusion/Exclusion Criteria6 | | **×** |  |  |  |  |  |  |  |  |  |  |  |  |  |  |  |
| Demographic Information | | **×** |  |  |  |  |  |  |  |  |  |  |  |  |  |  |  |
| Tumor Diagnosis and Treatment History | | **×** |  |  |  |  |  |  |  |  |  |  |  |  |  |  |  |
| Tumor Staging in Screening Period | | **×** |  |  |  |  |  |  |  |  |  |  |  |  |  |  |  |
| Medical History of Non-tumor 7 | | **×** |  |  |  |  |  |  |  |  |  |  |  |  |  |  |  |
| Concomitant Diseases 8 | | **×** |  |  |  |  |  |  |  |  |  |  |  |  |  |  |  |
| Previous Treatment 9 | | **×** |  | Evaluation methods should be decided by investigators based on the patients’ clinical indicators. The follow-up examination methods for the same patient should try to keep consistent. | | | | | | | | | | | | | |
| Imaging Evaluation 10 | | **×** |  | **×** | If the patient has signs or symptoms, such as heart failure, according to clinical indicators | | | | | | | | | | | | |
| Echocardiography | | **×** | **×** |  | **×** | **×** |  |  |  |  |  |  |  |  |  |  |  |
| Height/Weight/Body Surface Area 11 | | **×** | **×** | **×** |  | **×** | **×** | **×** | **×** | **×** | **×** | **×** | **×** | **×** | **×** | **×** | **×** |
| Vital Signs 12 | | **×** |  | **×** |  | **×** |  | **×** | **×** | **×** | **×** | **×** | **×** | **×** | **×** | **×** | **×** |
| Physical Examination | | **×** |  | **×** |  | **×** |  | **×** | **×** | **×** | **×** | **×** | **×** | **×** | **×** | **×** | **×** |
| ECOG Score | | **×** |  | **×** |  | **×** |  |  |  |  |  |  |  | **×** | **×** | **×** | **×** |
| 12-lead Electrocardiogram 13 | | **×** |  | **×** |  | **×** |  |  |  |  |  |  |  |  |  |  |  |
| Laboratory Tests | Blood Oxygen Saturation Test | **×** |  | Women of childbearing potential should be reviewed promptly if they experience suspected pregnancy, such as menopause, vomiting, abdominal pain, and vaginal bleeding, according to clinical indicators | | | | | | | | | | | | | |
|  | Blood Pregnancy test14 | **×** |  |  |  |  |  |  |  |  |  |  |  |  |  |  |  |
|  | Virology/Syphilis test15 | **×** | **×** | **×** |  | **×** | **×** | **×** | **×** | **×** | **×** | **×** | **×** | **×** | **×** | **×** | **×** |
|  | Hematology | **×** | **×** | **×** |  | **×** |  |  |  | **×** |  | **×** | **×** | **×** | **×** | **×** | **×** |
|  | Blood biochemical indexes | **×** | **×** | **×** |  | **×** |  |  |  | **×** |  | **×** | **×** | **×** | **×** | **×** | **×** |
|  | Coagulation | **×** | **×** | **×** |  | **×** |  |  |  | **×** |  | **×** | **×** | **×** | **×** | **×** | **×** |
|  | Urinalysis |  |  | **×** |  | **×** |  | **×** | **×** | **×** | **×** | **×** | **×** | **×** | **×** | **×** |  |
|  | Inflammatory Factors 16 | **×** |  | **×** |  |  |  |  |  |  |  | **×** |  | **×** | **×** | **×** | **×** |
| Efficacy Tests | Serum Calcium and Albumin-corrected Calcium | **×** |  | **×** |  |  |  |  |  |  |  | **×** |  | **×** | **×** | **×** | **×** |
|  | β2-microglobulin | **×** |  | **×** |  |  |  |  |  |  |  | **×** |  | **×** | **×** | **×** | **×** |
|  | Serum Immunoglobulin Quantification | **×** |  | **×** |  |  |  |  |  |  |  | **×** |  | **×** | **×** | **×** | **×** |
|  | Serum Free Light Chain Quantification | **×** |  | **×** |  |  |  |  |  |  |  | **×** |  | **×** | **×** | **×** | **×** |
|  | Serum Protein Electrophoresis | **×** |  | **×** |  |  |  |  |  |  |  | **×** |  | **×** | **×** | **×** | **×** |
|  | Serum Immunofixation Electrophoresis | **×** |  | **×** |  |  |  |  |  |  |  | **×** |  | **×** | **×** | **×** | **×** |
|  | 24 h Urine M Protein Quantification | **×** |  | **×** |  |  |  |  |  |  |  | **×** |  | **×** | **×** | **×** | **×** |
|  | 24 h Urine Light Chain Quantification | **×** |  | **×** |  |  |  |  |  |  |  | **×** |  | **×** | **×** | **×** | **×** |
|  | Urine Protein Electrophoresis | **×** |  | **×** |  |  |  |  |  |  |  | **×** |  | **×** | **×** | **×** | **×** |
|  | Urine Protein Immunofixation Electrophoresis | **×** |  | **×** |  |  |  |  |  |  |  | **×** |  | **×** | **×** | **×** | **×** |
| Peripheral Blood Smear | | **×** |  | **×** |  |  |  |  |  |  |  |  |  | **×** | **×** | **×** | **×** |
| Bone marrow biopsy18 | Cell Smear Morphology | **×** |  | **×** |  |  |  |  |  |  |  |  |  | **×** | **×** | **×** | **×** |
|  | Bone Marrow Biopsy Pathology + Immunohistochemistry | **×** |  |  |  |  |  |  |  |  |  |  |  |  |  |  |  |
|  | Cytogenetics (FISH) | **×** |  | **×** |  |  |  |  |  |  |  |  |  | **×** | **×** | **×** | **×** |
|  | Bone Marrow Flow Immunophenotyping Test (Including MRD Test) |  |  | **×** |  |  |  | **×** | **×** | **×** | **×** | **×** | **×** | **×** | **×** | **×** | **×** |
| Peripheral Blood Collection for CT103A Exploring Study 19 | |  |  | **×** |  |  |  | **×** | **×** | **×** | **×** | **×** | **×** | **×** | **×** | **×** | **×** |
| PK blood collection20 | CT103A VCN test |  |  | **×** |  |  |  | **×** | **×** | **×** | **×** | **×** | **×** | **×** | **×** | **×** | **×** |
|  | Peripheral Blood CT103A Cell Test |  |  |  |  |  |  |  |  |  |  |  |  | **×** | **×** | **×** | **×** |
|  | Peripheral Blood Selinexor Concentration |  | **×** |  |  |  |  |  |  |  |  |  |  |  |  |  |  |
| PBMC Collection | |  |  |  | **×** |  |  |  |  |  |  |  |  |  |  |  |  |
| Administration for Lymphodepletion conditioning | |  |  |  |  |  | **×** |  |  |  |  |  |  |  |  |  |  |
| Drugs to Prevent Infusion Reactions 21 | |  |  |  |  |  | **×** |  |  |  |  |  |  |  |  |  |  |
| CT103A Cells Infusion | |  |  |  |  |  |  |  |  |  |  |  |  | **×** | **×** | **×** | **×** |
| Administration of Selinexor 22 | |  |  | **×** |  |  |  |  |  |  |  | **×** |  | **×** | **×** | **×** | **×** |
| Assessment for Life Quality 23 | |  |  | **×** |  |  |  |  |  |  |  |  |  | **×** | **×** | **×** | **×** |
| Assessment for IMWG Treatment Efficacy | | **×** | **×** | **×** | **×** | **×** | **×** | **×** | **×** | **×** | **×** | **×** | **×** | **×** | **×** | **×** | **×** |
| AE 24 | | **×** | **×** | **×** | **×** | **×** | **×** | **×** | **×** | **×** | **×** | **×** | **×** | **×** | **×** | **×** | **×** |
| SAE 25 | | **×** | **×** | **×** | **×** | **×** | **×** | **×** | **×** | **×** | **×** | **×** | **×** | **×** | **×** | **×** | **×** |
| Concomitant Treatment | |  | **×** |  |  |  |  |  |  |  |  |  |  |  |  |  |  |
| Bridging Therapy | |  |  |  |  |  |  |  |  |  |  |  |  |  |  |  |  |

Abbreviations: AE, adverse event; BSA, body surface area; d, day; CR, complete response; ECOG, Eastern Cooperative Oncology Group; HBV, hepatitis B virus; HBcAb, hepatitis B core antibody; HBeAb, hepatitis B e antibody; HBeAg, hepatitis Be antigen; HBsAb, hepatitis B surface antibody; HBsAg, hepatitis B surface antigen; HCV, hepatitis C virus; ICF, informed consent form; IMWG, International Myeloma Working Group; MRD, minimal residual disease; PBMC, peripheral blood mononuclear cell; PET-CT, positron emission tomography-computed tomography; PFS, progression-free survival; PK, pharmacokinetic; SAE, serious adverse event; sCR, stringent complete response; SOP, standard operating procedure; VCN, vector copy number; w, week.

1. Patients should complete the visits according to the schedule of assessments during the screening period. Patients who meet the enrollment criteria will be allocated a patient number after the investigators' assessment. It will not be regarded as a protocol violation if the time from the patient's signing of the informed consent to the infusion exceeds eight weeks as specified in the protocol because of delays in the lymphodepletion conditioning and/or cell infusion during the screening period and the cell preparation period (-8 w to -8 d). It will not be regarded as a protocol violation if the time from PBMC collection to cell infusion exceeds 18 days because of reasons such as the making and preparation of CAR-T cells.
2. The PBMC collection visit should be evaluated within one day of cell collection, with blood biochemical indexes, coagulation, and urinalysis tests performed within seven days. Patients who meet the criteria of mononuclear cell collection will enter into the PMBC collection process according to the SOP for collecting PBMCs.
3. The baseline assessments will be completed within seven days before the lymphodepletion conditioning (vital signs and physical examination will be conducted within one day before the lymphodepletion conditioning). Lymphodepletion conditioning can be conducted when the results of each test meet the criteria of lymphodepletion treatment. The results of the baseline assessments within 14 days before the lymphodepletion conditioning are acceptable if lymphodepletion conditioning delays. For bone marrow aspiration/biopsy-related tests at baseline, the results are acceptable within four weeks for non-secretory patients, and the investigator will assess whether a re-test is required for secretory patients. Before lymphodepletion conditioning, related-examination results should be confirmed again to ensure that patients meet the criteria of lymphodepletion conditioning.
4. Patients must be re-assessed by the investigator before the cell infusion. If the patient does not meet the cell infusion criteria, a delay of no more than two weeks is acceptable. If the delay exceeds two weeks, the investigator will evaluate if a repeat of the baseline assessments and lymphodepletion conditioning is needed. The inpatient observation should be at least 14 days from the date of cell transfusion. The inpatient observation from 15 to 28 days should be in compliance with the local practice. Prolongation of hospitalization due to medical norms will not be recorded as an SAE.
5. The main follow-up period is one day after the cell infusion to 1 year after the cell infusion. After three months of cell infusion, follow-up visits will be conducted every three months until disease progression or until the withdrawal of informed consent, death, loss of follow-up, or the termination of the study, whichever occurs first.
6. During the screening period, patients must meet all inclusion criteria and none of the exclusion criteria to be enrolled.
7. Prior medical history is recorded if the patients’ previous surgery history or non-tumor chronic diseases has recovered or returned to the state of no clinical significance during the screening period.
8. Any clinically significant abnormal symptoms, signs, or laboratory findings of a non-neoplastic chronic disease that is still clinically significant during the Screening Period or that are not associated with the disease under study after the signing of the ICF and before the collection of PBMC should be recorded as a concomitant disease. Abnormal results caused by the study procedure during the screening period should be recorded as AE.
9. After signing the ICF, all medications and non-medication treatments should be recorded, including nutritional supplements, such as vitamins, solvents with therapeutic effects, or solvents.
10. Imaging assessments include the use of PET-CT during the screening period, and PET-CT should also be used when the investigator judges that CR/sCR may be achieved. In addition to the conditions mentioned above, the imaging evaluation method at other time points should be decided by the investigators based on the patients’ clinical indicators. The imaging methods for the same patient should keep consistent during follow-up. Imaging assessment results within four weeks are acceptable during the screening period.
11. The height and weight will be measured during the screening period, and the body weight measurement is required for rest assessment within 1 day before the mononuclear cell collection. The height and weight should be re-measured before the lymphodepletion conditioning to calculate the BSA. Calculation formula of the BSA: BSA (m²) = SQRT (height cm × weight kg/3600).
12. Vital signs should be closely monitored on the day of CT103A cell infusion (D0). If vital signs are unstable, monitoring should be continued until all measurements are stable. Emergency facilities should be available during the infusion to allow for prompt management of severe allergic reactions, severe hypotension, or other reactions.
13. An electrocardiogram or echocardiography can be performed at the discretion of the investigator based on the patient’ symptoms, signs, or abnormal heart function during CT103A cell infusion and follow-up observation.
14. For women of childbearing potential only, a blood pregnancy test should be performed at the out-of-study visit if the out-of-study visit is completed within one year after CAR-T infusion.

Infertile women are defined as: 1, postmenopausal women (with at least one year of menopause), 2, women with bilateral ovaries or hysterectomy.

1. Including HCV antibody, five items of HBV (HBsAg, HBsAb, HBeAg, HBeAb and HBcAb), HIV antibody, EBV DNA (the results are not used as a criterion to enroll the study), cytomegalovirus DNA, and syphilis. Patients with a positive HCV antibody should be tested for HCV RNA, and the patients can be enrolled if HCV RNA is negative. Both HBV surface antigen and HBV core antibody should be negative. A peripheral HBV DNA test is required if either of the tests mentioned above is positive. When HBV DNA is below the normal limit, the patient can be enrolled. The results mentioned above within four weeks before the screening period are acceptable during the screening period.
2. Inflammatory factors should include IL-6 and ferritin, at least.
3. If non-secretory multiple myeloma is confirmed during the screening and baseline period, the test may not be performed at subsequent follow-up visits (except for serum calcium and albumin-corrected calcium, β2-microglobulin)
4. The results of bone marrow within two weeks are acceptable during the screening or baseline period (provided that the test items meet the requirements of the study and a re-test will be performed in case of any missed item). The cytogenetic test is only required during the screening period and may be repeated during the baseline period if it fails or is not performed during the screening period (if FISH tests fail at both the screening period and the baseline period, the previous test results are acceptable). The results of bone marrow immunohistochemistry or bone marrow flow cytometry are acceptable for the BCMA expression test at the screening period. During the follow-up period after infusion, bone marrow aspiration/biopsy is needed if the investigator determines that CR/sCR may be achieved or disease progression occurs, and at other follow-up visits, the investigator may assess the need for bone marrow aspiration/biopsy test. However, tests for non-secretory patients are required at each visit. Bone marrow aspiration/biopsy-related tests are not required for patients without bone marrow infiltration.
5. An additional 3 mL of the blood sample will be collected for each PK blood sample collection for exploring studies.
6. PK blood sample collection will be used for CT103A and VCN testing until the out-of-study visit or the termination of the study, whichever occurs first. Complete blood count (CBC) should be performed simultaneously with PK blood collection. Results of CBC within 24 hours from PK blood collection are acceptable if CBC is not performed during the day.
7. Medications that prevent infusion reactions, such as promethazine and/or diphenhydramine hydrochloride, promethazine, and calcium gluconate, will be administered before the infusion of CAR-T cells.
8. At least a month after CT103A infusion and platelets recovery to ≥ 50×10^9^/L, Selinexor 20 mg or 40 mg will be taken orally once a week for 1 year or until the disease progression or withdrawal of informed consent, death, loss of follow-up, and the termination of the study, whichever occurs first.
9. Life quality assessment is conducted at the baseline, 28d, and follow-up visits. The quality of Life Questionnaire (EORTC-QLQ-C30) of the European Organisation for Research and Treatment of Cancer will be used to evaluate patients’ health-related life quality. The European Quality of Life Multiple Myeloma Module (EORTC-QLQ-MY20) will be used to evaluate myeloma-related life quality until the withdrawal of informed consent, death, loss of follow-up, and the termination of the study, whichever occurs first.
10. AEs should be recorded from the start of lymphodepletion conditioning. AEs should be collected until one year after CT103A cell infusion (if PFS ≥12 months) or at the out-of-study visit (12 months > PFS ≥ 3 months), and if disease progression occurs within three months after CT103A cell infusion, AEs are collected for up to 3 months after cell infusion, under the patients’ cooperation.
11. SAEs should be reported from the signing of the ICF.

Medication and non-medication treatment should be recorded as concomitant treatments from signing the ICF. SAE should be collected until 1 year after CT103A cells infusion (if PFS≥12 months) or at the out-of-study visit (12 months > PFS ≥ 3 months). SAE should be collected for up to 3 months after cells infusion under the patient’s cooperation if the disease progression occurs within three months after CT103A cell infusion

## Screening and Enrollment Period (day -56 to day -18)

### Screening Period

The following information/assessment results will be collected during the screening:

- - - 1. Sign the informed consent.
      2. Review the inclusion/exclusion criteria.
      3. Collect the demographic information (including date of birth, gender, race, etc.); record the prior medical history, concomitant diseases, and prior treatment: past medical history includes a review of the medical history of cardiovascular, pulmonary, gastrointestinal, and liver disease, including the related surgy; a detailed history of MM to be collected in the history section of tumor diagnosis and treatment, including but not limited to:
- Diagnosis, classification and staging, and hazard level classification at initial diagnosis and screening.
- Previous anti-tumor medication (including treatment plan, medication name, start and end dates of each medication, reasons for treatment, reasons for discontinuation of each drug, and the best efficacy of each regimen).
- Tumor-related radiotherapy (including body parts, total dose, units, start and end dates, the reason for treatment, optimal efficacy obtained, and the date of progression in the radiation region at or after the treatment).
- Symptoms and signs before medication, including the progression of the medical history.
  - - 1. Body height/weight/body surface area.
      2. Physical examination.
      3. Vital signs: include heart rate, blood pressure, and respiratory rate, which should be measured after the patient has rested for at least 5 minutes, axillary temperature, which is measured at least for more than 5 minutes; ECOG score. Subsequent visits should be conducted according to this process.
      4. Echocardiogram examination.
      5. 12-lead electrocardiogram (ECG) (Heart rate, PR interval, RR interval, duration of QRS, and QT interval corrected by heart rate using Fridericia formula [QTcF] should be measured after at least 5 minutes of rest. Then determine the clinical significance of abnormal values. Subsequent visits should be conducted according to this process).
      6. Oxygen saturation test.
      7. Blood pregnancy test in women of childbearing potential.
      8. Virology/syphilis testing: including HCV antibody, five items of HBV (hepatitis B surface antigen [HBsAg], hepatitis B surface antibody [HBsAb], hepatitis Be antigen [HBeAg], hepatitis B e antibody [HBeAb], and hepatitis B core antibody [HBcAb]), HIV antibody, EBV DNA (the results are not used as a criterion to enroll the study), cytomegalovirus DNA, and syphilis. Patients with a positive HCV antibody should be tested for HCV RNA, and the patient can be enrolled if HCV RNA is negative. Both the HBV surface antigen and HBV core antibody should be negative. A peripheral HBV DNA test is required if either of the abovementioned tests is positive. When HBV DNA is below the normal limit, the patient can be enrolled. The results mentioned above within four weeks before the screening period are acceptable during the screening period.
      9. Complete blood count.
      10. Blood biochemical indexes.
      11. Urinalysis.
      12. Coagulation.
      13. Peripheral blood smear test.
      14. Serum calcium and albumin test.
      15. β2-microglobulin test.
      16. Quantification of serum immunoglobulin.
      17. Quantification of serum free light chain.
      18. Serum protein electrophoresis.
      19. Serum immunofixation electrophoresis.
      20. Quantification of 24 h urine M protein.
      21. Quantification of 24 h urine light chain.
      22. Urine protein electrophoresis.
      23. Urine protein immunofixation electrophoresis.
      24. Imaging evaluation: low-dose whole-body computed tomography (CT) or positron emission tomography (PET)-CT can be used for skeletal examination during the screening period. If there is no low-dose whole-body CT or PET-CT, local CT/magnetic resonance imaging (MRI) can be used instead. Besides the screening period, additional imaging evaluation test is determined by the investigator according to the clinical indications (e.g., suspicion of CR or disease progression). The examination methods of the same patient should be consistent during the follow-up period. PET-CT can be added to determine the activity of the tumor in patients who still have a mass detected by CT or MRI examination and those who are not compatible with the condition as judged by the investigator. The results of imaging evaluation within four weeks are accepted before the screening, and subsequent visits should be conducted according to this process.
      25. Bone marrow aspiration includes cell smear morphology, bone marrow biopsy pathology + immunohistochemistry, cytogenetics (FISH), and bone marrow immunophenotyping tests by flow cytometry, including MRD. Recommendations for Bone marrow immunohistochemistry include, but are not limited to, BCMA, CD19, CD20, CD38, CD56, CD138, κ light chain, and λ light chain. Bone marrow flow cytometry suggests more than eight colors for antibody labeling, including but not limited to BCMA, CD19, CD38, CD45, CD56, CD20, CD138, κ light chain of cytoplasm, λ light chain of cytoplasm. The examination of bone marrow immunohistochemistry or bone marrow by flow cytometry could be accepted during the screening period. FISH method is used for cytogenetic examination (CD138^+^ magnetic beads are suggested for sorting myeloma cells or concurrent cytoplasmic immunoglobulin staining to differentiate plasma cells). Detection sites recommended include but are not limited to IgH translocation, 17p- (p53 deletion), 13q14 deletion, and 1q21 amplification. If FISH detection shows that IgH translocation is positive, it is recommended to further detect t (4; 14), t (11; 14), t (14; 16), t (14; 20). The cytogenetic examination is only required during the screening period. If the screening test fails or is not performed, it can be tested again at the baseline assessments. The results of the bone marrow examination within two weeks are acceptable in the screening period (but the examination items have to meet the requirements of this study, if there is a missing item, the specimen needs to be collected again).
      26. Record AEs and SAEs: AEs and SAEs will be collected from signing the informed consent. Results from the screening period will not be recorded as AEs unless the study procedure causes them. AEs should be collected until one year after CT103A cell infusion (if PFS ≥ 12 months) or at the out-of-study visit (12 months > PFS ≥ 3 months). If disease progression occurs within three months after the CT103A cell infusion, AEs are collected for up to three months after the cell infusion, under the patients' cooperation. All events that meet the reporting criteria of SAE should be reported. If a second malignant tumor occurs, it should be reported as an SAE within one year after cell infusion.
      27. Record concomitant treatment: After signing the ICF, all medications and non-medication treatments should be recorded, including nutritional supplements, such as vitamins, solvents with therapeutic effects, or solvents. It should be collected until one year after CT103A cell infusion (if PFS ≥12 months) or at the out-of-study visit (12 months > PFS ≥ 3 months). If disease progression occurs within three months after CT103A cell infusion, they are collected for up to three months after the cell infusion, under the patient' s cooperation.

Notes:

- If the patient is confirmed as a non-secretory MM during the screening and baseline period, the tests (19) to (26) will not be performed in subsequent follow-up visits.
- A Repeat test during the screening period is permitted in this study, including but not limited to laboratory abnormalities or incorrect test values. The last test results obtained after supportive treatment and re-test will be used to determine whether the patients are eligible to participate in this study. The test results within seven days before the enrollment are acceptable before the re-test, such as CBC, blood biochemical indexes, coagulation, and urinalysis. However, other tests will be required to meet the window period listed by the protocol.
- The patient who fails in screening will be allowed for a second screening, which will require re-signing the informed consent form, and the patient will be assigned a new patient number.
- One patient may receive only one repeat test or re-screening.

### Enrollment

Patients should complete the visits according to the schedule of assessments during the screening period. Patients who meet the enrollment criteria will be allocated a patient number after the investigators' assessment.

## PBMC Collection (day -56 to day -18)

Apheresis is performed according to the standard practice of the institution, the goal is to deliver it to the preparation site with a minimum of 1 × 10^9^ PBMCs in an apheresis sample (to ensure success rate of cell product preparation, it is suggested to collect more than 2 × 10^9^ PBMCs), and two times of apheresis may be arranged to achieve this target amount.

If disease progression occurs after PBMC collection, the investigator may decide whether to administer bridging therapy.

The assessments during the visit of the PBMC collection are as follows:

- - - 1. Body height/weight/body surface area.
      2. Vital signs: include heart rate, blood pressure, respiratory rate, and axillary temperature.
      3. CBC.
      4. Laboratory tests (blood biochemical indexes [fasting], coagulation, and urinalysis).
      5. Review of apheresis standard.
      6. PBMC collection (additional 3 mL is collected for exploratory studies).
      7. Record AEs and SAEs.
      8. Record concomitant treatment and bridging therapy.

**Secondary Apheresis**

Secondary apheresis is allowed before the baseline assessment. To ensure a minimum of 1×10^9^ PBMC in the apheresis specimen, two times of apheresis will be arranged. The standard of apheresis should be reviewed again before the secondary apheresis to assess whether it is suitable for secondary apheresis. Secondary apheresis cannot be performed under the following situation:

- The quality problems of mononuclear cells of the patients themselves lead to unsuccessful CAR-T cell preparation.
- Unsuccessful CAR-T cell preparation due to non-compliance with the standard of apheresis.
- The investigator’s comprehensive assessment is not appropriate for secondary apheresis.

## Baseline Assessments (within 7 d before Lymphodepleting Conditioning)

The baseline assessments will be completed within seven days before the lymphodepletion conditioning (vital signs and physical examination will be conducted within one day before the lymphodepletion preconditioning). Lymphodepletion conditioning can be conducted when the results of each test meet the criteria of lymphodepletion treatment. The results of the baseline assessments within 14 days before the lymphodepletion conditioning are acceptable if lymphodepletion conditioning delays. For bone marrow aspiration/biopsy-related tests at baseline, the results are acceptable within four weeks for non-secretory patients, and the investigator will assess whether a re-test is required for secretory patients. Before lymphodepletion conditioning, related-examination results should be confirmed again to ensure that patients meet the criteria of lymphodepletion conditioning.

The assessments during the Baseline are as follows:

- - - 1. Physical examination.
      2. Vital signs: include heart rate, blood pressure, axillary temperature, and respiratory rate; ECOG score.
      3. 12-lead ECG examination.
      4. Echocardiography examination.
      5. Oxygen saturation test.
      6. Laboratory tests (CBC [collected with PK blood at the same time, with a maximum time difference of 24 hours], blood biochemical indexes [fasting], coagulation, and urinalysis).
      7. Inflammatory factors.
      8. Peripheral blood smear test.
      9. Serum calcium and albumin corrected calcium.
      10. β2-microglobulin test.
      11. Quantification of serum immunoglobulin.
      12. Quantification of serum free light chain.
      13. Serum protein electrophoresis.
      14. Serum immunofixation electrophoresis.
      15. Quantification of 24 h urine M protein.
      16. Quantification of 24 h urine light chain.
      17. Urine protein electrophoresis.
      18. Urine protein immunofixation electrophoresis.
      19. Bone marrow aspiration includes cell smear morphology, bone marrow biopsy pathology + immunohistochemistry (decided by the investigator), and bone marrow flow immunophenotyping tests, including MRD; the results within two weeks before the baseline assessments are acceptable. A cytogenetic examination is only required during the screening period. If the screening test fails or is not performed, it can be tested again at the baseline assessments.
      20. Imaging evaluation: the assessment method is determined by the investigator according to the clinical indications of the patient.
      21. Blood pregnancy test (only for women of childbearing potential): based on clinical indications, women of childbearing potential should have a repeat test promptly if they are suspected of pregnancy, such as menopause, vomiting, abdominal pain, or vaginal bleeding.
      22. PK blood collection (an additional 3 mL is collected for exploratory studies).
      23. VCN test:
      24. Peripheral blood CT103A cell test.
      25. Record concomitant treatment/AE/SAE.
      26. IMWG efficacy evaluation.
      27. Quality of life assessment.

## Lymphodepleting Conditioning（day -7 to day -2）

The visit for lymphodepleting conditioning should be performed within one week before the cell infusion, CT103A should be frozen after preparation, and the investigator will perform lymphodepleting conditioning according to the patient s condition. Height, weight, and body surface area will be measured before the lymphodepleting conditioning.

The assessments during the lymphodepleting conditioning visit are as follows:

- - - 1. Review the lymphodepleting conditioning criteria.
      2. Height, weight, body surface area: measure the height and weight again, and calculate the body surface area.
      3. Administration of lymphocyte clearance pretreatment.
      4. Record concomitant treatment, AEs, and SAEs.

## Rest Assessment Before Treatment (day -1)

If the patient's state does not meet the criteria of infusion during the rest assessment, the investigator can provide symptomatic treatment until his/her state can be treated with cell infusion. The delayed infusion should not exceed two weeks.

The rest assessments are as follows:

- - - 1. Weight.
      2. Physical examination.
      3. Vital signs: include heart rate, blood pressure, axillary temperature, and respiratory rate; ECOG score.
      4. 12-lead ECG: if there is suspected abnormal cardiac function, an additional examination will be performed according to the symptoms and signs of the patient.
      5. Echocardiogram examination: according to the clinical indications, the investigator will perform an echocardiogram examination appropriately if the patient has signs and symptoms, such as heart failure.
      6. Oxygen saturation test.
      7. Blood pregnancy test (only for women of childbearing potential): based on clinical indications, women of childbearing potential should have a repeat test promptly if they are suspected of pregnancy, such as menopause, vomiting, abdominal pain, or vaginal bleeding.
      8. Laboratory tests (CBC, blood biochemical indexes [fasting], coagulation, and urinalysis).
      9. Inflammatory factors: The test of inflammatory factors should include IL-6 and ferritin, at least.
      10. Record concomitant treatment, AEs, and SAE.

## CT103A Cell Infusion (day 0)

Cell infusion visit should be completed under inpatient conditions.

The assessments in this visit are as follows:

- - - 1. Review of infusion criteria before infusion.
      2. Vital signs: include heart rate, blood pressure, axillary temperature, and respiratory rate.

For vital signs: Vital signs (axillary temperature, blood pressure, respiratory rate, and heart rate) should be measured before the cell infusion, at the start of the cell infusion, and within 1 hour (every 15 minutes ± 3 minutes) after the cell infusion until all the measures are stable and in safety state (Table 2).

Table 2. Monitoring Requirement of Vital Signs

| Time | Before the start of the cell infusion | At the start of the cell infusion | After the start of cell infusion | | | |
| --- | --- | --- | --- | --- | --- | --- |
|  | -15 min ± 3 min | 0 min | 15 min ± 3 min | 30 min ± 3 min | 45 min ± 3 min | 60 min ± 3 min |
| Axillary  temperature | √ | √ | √ | √ | √ | √ |
| Blood pressure | √ | √ | √ | √ | √ | √ |
| Respiratory | √ | √ | √ | √ | √ | √ |
| Heart rate | √ | √ | √ | √ | √ | √ |

Note: Emergency facilities should be available during the cell infusion to allow for prompt management of severe allergic reactions, severe hypotension, and other reactions.

- - - 1. CBC: perform as needed according to clinical indications.
      2. Echocardiogram examination: According to the clinical indications, the investigator will perform the echocardiogram examination appropriately if the patient has signs and symptoms, such as heart failure.
      3. Blood pregnancy test (only for women of childbearing potential): based on clinical indications, women of childbearing potential should have a repeat test promptly if they are suspected of pregnancy, such as menopause, vomiting, abdominal pain, or vaginal bleeding.
      4. Medications for preventing infusion reactions.
      5. CT103A cell infusion.
      6. Record concomitant treatment, AEs, and SAEs.

## Main Follow-up Period (day 1 to 1 year after Cell Infusion)

### Visits during inpatient observation and within 28 days after the cell infusion (day 1 to day 28)

The inpatient observation period should be at least 14 days, and hospitalization from 15 to 28 days should be conducted according to local practice. The window period is ± 12 h for 7 d, 10 d, and 14 d visits and ± 2 d for 21 d and 28 d visits.

The assessments during the main follow-up period are as follows:

- - - 1. Physical examination, vital signs, ECOG score: day 1, 4, 7, 10, 14, 21, 28.
      2. 12-lead ECG: based on clinical indications.
      3. Echocardiogram examination: according to the clinical indications, the investigator will perform an echocardiogram examination appropriately if the patient has signs and symptoms, such as heart failure.
      4. Oxygen saturation test: Oxygen saturation will be measured at least twice daily during CRS (the interval between the two measurements should be at least 6 hours).
      5. Blood pregnancy test (only for women of childbearing potential): based on clinical indications, women of childbearing potential should have a repeat test promptly if they are suspected of pregnancy, such as menopause, vomiting, abdominal pain, or vaginal bleeding.
      6. CBC: day 1, 4, 7, 10, 14, 21, 28. PK samples will be collected at the same time points, with a maximum time difference of 24 hours.
      7. Blood biochemical indexes (fasting): day 7, 14, 21, 28.
      8. Urinalysis: day 7, 14, 21, 28.
      9. Coagulation: day 7, 14, 21, 28.
      10. Serum calcium and albumin-corrected calcium: day 14 and day 28.
      11. Inflammatory factors: day 1, 4, 7, 10, 14, 21, 28.
      12. Peripheral blood smear test: day 14 and day 28.
      13. β2-microglobulin test: day 14 and day 28.
      14. Quantification of serum immunoglobulin: day 14 and day 28.
      15. Quantification of serum free light chain: day 14 and day 28.
      16. Serum protein electrophoresis: day 14 and day 28.
      17. Serum immunofixation electrophoresis: day 14 and day 28.
      18. Quantification of 24 h urine M protein: day 14 and day 28.
      19. Quantification of 24 h urine light chain: day 14 and day 28.
      20. Urine protein electrophoresis: day 14 and day 28.
      21. Urine protein immunofixation electrophoresis: day 14 and day 28.
      22. Bone marrow aspiration/biopsy (required if the investigator determines CR/sCR is achieved or disease progression occurs, the investigator could determine in other follow-up visits whether bone marrow aspiration/biopsy is required; however, for non-secretory patients, tests are required at each when efficacy is assessed): 28 d (including cell smear morphology, bone marrow biopsy pathology + immunohistochemistry, bone marrow immunophenotyping test by flow cytometry including MRD).
      23. Imaging evaluation: the assessment method is determined by the investigator according to the clinical indications. The examination methods of the same patient should be consistent during the follow-up period.
      24. Peripheral blood CT103A cell test: day 1, 4, 7, 10, 14, 21, 28.
      25. PK blood collection (an additional 3 mL is collected for exploratory studies): day 1 (time window ±1 hour), day 4 (time window ±3 hours), day 7 (time window ±12 hours), day 10 (time window ±12 hours), day 14 (time window ±12 hours), day 21 (time window ± 2 days), day 28 (time window ± 2 days).
      26. VCN test: day 1, 4, 7, 10, 14, 21, 28. dPCR test method will be used.
      27. IMWG efficacy evaluation: day 28. IMWG efficacy criteria and MRD efficacy criteria will be used.
      28. Record concomitant treatment, AEs, and SAEs: day 1, 4, 7, 10, 14, 21, 28.
      29. Quality of life assessment: day 14 and day 28.

### Selinexor Administration

One month after anti-BCMA CAR-T cells infusion and platelet recovery, Selinexor will be administered once a week (20 mg/week or 40 mg/week).

### Subsequent Follow-up Visits

It will be performed at two months (day 60), three months (day 90) after the cell infusion, and once every three months thereafter, until disease progression or until the withdrawal of informed consent, withdrawal from the study, death, or one year after the cell infusion, whichever occurs first. The visit window period is ±14 days for two months (day 60), ±14 days at three months (day 90), and ±14 days for every three months (90 d) thereafter.

The assessments during the subsequent follow-up visits are as follows:

- - - 1. Physical examination/vital sign/ECOG score: it will be performed at two months (day 60), three months (day 90), and once every three months (90 days) thereafter, until out-of-study visit or one year after the cell infusion (whichever occurs first).
      2. Echocardiogram examination: According to the clinical indications, the investigator will perform an echocardiogram examination appropriately if the patient has signs and symptoms, such as heart failure.
      3. 12-lead ECG: according to clinical indications, it will be performed at two months (day 60), three months (day 90), and once every three months (90 d) thereafter, until out-of-study visit or one year after the cell infusion, whichever occurs first.
      4. Blood pregnancy test (only for women of childbearing potential): based on clinical indications, women of childbearing potential should have repeat tests promptly if they are suspected of pregnancy, such as menopause, vomiting, abdominal pain, or vaginal bleeding;
      5. CBC: it will be measured at two months (day 60), three months (day 90), and once every three months (90 d) thereafter, until one year after the CT103A cell infusion. The subsequent tests will be performed as needed by the investigators according to clinical indications. PK samples will be collected at the same time points, with a maximum time difference of 24 hours.
      6. Blood biochemical indexes (fasting): it will be measured at two months (day 60), three months (day 90), and once every three months (90 d) thereafter, until one year after the CT103A cell infusion. The subsequent tests will be performed as needed by the investigator according to clinical indications.
      7. Urinalysis: it will be measured at two months (day 60), three months (day 90), and once every three months (90 d) thereafter, until one year after the CT103A cell infusion. The subsequent tests will be performed as needed by the investigator according to clinical indications.
      8. Coagulation: it will be measured at two months (day 60), three months (day 90), and once every three months (90 d) thereafter, until one year after the CT103A cell infusion. The subsequent tests will be performed as needed by the investigator according to clinical indications.
      9. Serum calcium and albumin corrected calcium: it will be measured at two months (day 60), three months (day 90), and once every three months (90 d) thereafter until out-of-study visit.
      10. Inflammatory factors: two months (day 60), three months (day 90);
      11. Peripheral blood smear test: it will be performed at two months (day 60), three months (day 90), and once every three months (90 d) thereafter until out of study visit; 24 months (720 d).
      12. β2-microglobulin test: it will be performed at two months (day 60), three months (day 90), and once every three months (90 d) thereafter until out-of-study visit.
      13. Quantification of serum immunoglobulin: it will be tested at two months (day 60), three months (day 90), and once every three months (90 d) thereafter until out-of-study visit.
      14. Quantification of serum free light chain: it will be tested at two months (day 60), three months (day 90), and once every three months (90 d) thereafter until out-of-study visit.
      15. Serum protein electrophoresis: it will be performed at two months (day 60), three months (day 90), and once every three months (90 d) thereafter until out-of-study visit.
      16. Serum immunofixation electrophoresis: it will be performed at two months (day 60), three months (day 90), and once every three months (90 d) thereafter until out-of-study visit.
      17. Quantification of 24 h urine M protein: it will be performed at two months (day 60), three months (day 90), and once every three months (90 d) thereafter until out-of-study visit.
      18. Quantification of 24 h urine light chain: it will be performed at two months (day 60), three months (day 90), and once every three months (90 d) thereafter until out-of-study visit.
      19. Urine protein electrophoresis: it will be performed at two months (day 60), three months (day 90), and once every three months (90 d) thereafter until out-of-study visit.
      20. Urine protein immunofixation electrophoresis: it will be performed at two months (day 60), three months (day 90), and once every three months (90 d) thereafter until out-of-study visit.
      21. Bone marrow aspiration (required if the investigator determines CR/sCR is achieved or disease progression occurs, other follow-up visits could be determined by the investigator whether bone marrow aspiration/biopsy is required; however, for non-secretory patients, tests are required at each when efficacy is assessed): 3 months (day 90), a re-examination of once every three months (90 days) thereafter until out of study visit (including cell smear morphology, bone marrow biopsy pathology + immunohistochemistry, bone marrow immunophenotyping test by flow cytometry including MRD);
      22. Imaging evaluation: the assessment method is determined by the investigator according to the clinical indications of the patient. The examination methods of the same patient should be consistent during the follow-up period.
      23. PK blood collection (an additional 3 mL is collected for exploratory studies); venous blood will be collected according to the laboratory manual of the study center for VCN assay and peripheral blood CT103A cell test. PK blood collection will be performed at day 60 (time window is ±14 days), day 90 (time window is ±14 days), and once every three months (time window is ±14 days for every 90 days) thereafter until out-of-study or termination of the study, which occurs first.
      24. IMWG efficacy evaluation: it will be performed at two months (day 60), three months (day 90), and once every three months (90 days) thereafter until out-of-study visit; IMWG efficacy criteria and MRD efficacy criteria will be used for assessment.
      25. Record concomitant treatment: it will be performed at two months (day 60), three months (90 d), and once every three months (90 d) thereafter until one year after the CT103A cell infusion (if PFS ≥ 12 months) or out of study visit (12 months > PFS ≥ 3 months). If disease progression occurs within three months after the CT103A cell infusion, AEs will be collected for up to 3 months after the cell infusion, under the patient' s cooperation.
      26. AEs: AEs should be collected at two months (day 60), three months (day 90), and once every three months (90 d) thereafter until one year after the CT103A cell infusion (if PFS ≥ 12 months) or out of study visit (12 months > PFS ≥ 3 months). If disease progression occurs within three months after the CT103A cell infusion, AEs will be collected for up to 3 months after the cell infusion, under the patient’ s cooperation.
      27. SAEs: SAEs should be collected at two months (day 60), three months (day 90), and once every three months (90 d) thereafter until one year after the CT103A cell infusion (if PFS ≥ 12 months) or out of study visit (12 months > PFS ≥ 3 months). If disease progression occurs within three months after the CT103A cell infusion, SAEs will be collected for up to 3 months after cell infusion, under the patient' s cooperation.
      28. Quality of life evaluation: it will be performed at two months (day 60), three months (day 90), and once every three months (90 d) thereafter until the withdrawal of informed consent, death, loss to follow-up, or termination of the study, whichever occurs first.

## Withdrawal from the study

Out of study refers to the patients who are no longer receiving subsequent follow-up due to the disease progression, initiation of new anti-tumor therapy, or other reasons.

If a new anti-tumor therapy has been initiated before the completion of the out-of-study visit unless the patient withdraws informed consent, all assessments of the out-of-study visit should be completed as much as possible within the time window. Meanwhile, diagnostic evidence in other hospitals and the details of anti-tumor therapy should be recorded according to chronological order.

If the patient does not conduct an out-of-study visit at the scheduled time point, it will be considered an unscheduled visit.

The assessments to be completed in the out-of-study visit are as follows:

- - - 1. Physical examination/vital sign/ECOG score (if the visit is within one year after the CT103A cell infusion).
      2. CBC CBC (if the visit is within one year after the CT103A cell infusion).
      3. Blood biochemical indexes (fasting) (if the visit is within one year after the CT103A cell infusion).
      4. Urinalysis (if the visit is within one year after the CT103A cell infusion).
      5. Coagulation (if the visit is within one year after the CT103A cell infusion).
      6. Virology/syphilis test.
      7. Echocardiogram examination (if the visit is within one year after the CT103A cell infusion).
      8. 12-lead ECG (if the visit is within one year after the CT103A cell infusion).
      9. Peripheral blood smear test.
      10. Serum calcium and albumin corrected calcium.
      11. β2-microglobulin test.
      12. Inflammatory factor (if the visit is within one year after CT103A cell infusion).
      13. Quantification of serum immunoglobulin.
      14. Quantification of serum free light chain.
      15. Serum protein electrophoresis.
      16. Serum immunofixation electrophoresis.
      17. Quantification of 24 h urine M protein.
      18. Quantification of 24 h urine light chain.
      19. Urine protein electrophoresis.
      20. Urine protein immunofixation electrophoresis.
      21. Bone marrow aspiration includes cell smear morphology, bone marrow biopsy pathology + immunohistochemistry, cytogenetics (FISH), and bone marrow immunophenotyping tests by flow cytometry, including MRD.
      22. It is determined by the investigator according to the clinical indications. The examination methods of the same patient should be consistent during the follow-up period.
      23. Peripheral blood CT103A cell test.
      24. PK blood collection (an additional 3 mL is collected for exploratory studies).
      25. VCN Test:
      26. IMWG efficacy evaluation.
      27. Blood pregnancy test (if the visit is within one year after the CT103A cell infusion).
      28. Record concomitant treatment.
      29. AEs.
      30. SAEs.
      31. Subsequent tumor treatment.

## Unscheduled visits

Unscheduled visits that occur during the study and after the out-of-study should be recorded in the original document and eCRF. During the unscheduled visits, vital signs, physical examinations, AEs, concomitant medications, treatment, and changes should be recorded. If the patient withdraws from the unscheduled visit, the above details should be recorded in the original document and the withdrawal section of the eCRF.

# SAFETY ASSESSMENTS

Safety assessments include AE assessments, ECOG performance status scores, laboratory tests, vital signs, physical examination, ECG, echocardiogram, etc.

## Adverse Event

### Definition of Adverse Events

An AE is any untoward medical occurrence in a patient which does not necessarily have a causal relationship with the study drug. It can be any symptom, sign, disease, condition, or laboratory test abnormality that occurs after administration of the study drug.

AEs include any event that develops or worsens in severity or frequency from baseline conditions, including laboratory test abnormalities.

AEs do not include:

- Medical or surgical procedures (e.g., surgery, endoscopy, tooth extraction, transfusion) for which the condition is causing these procedures should be reported as an AE.
- Existing diseases or conditions at Screening but not worsened afterward, including laboratory test abnormalities.
- Anticipated progression of the disease and/or anticipated progression of the symptoms and signs of the disease unless greater than expected.

### Serious Adverse Event

An SAE is defined as any AE that results in any of the following in a patient that occurs after infusion of the study drug (at any dose):

- Results in death.
- Life-threatening: an AE is life-threatening if the patient is at immediate risk of death as it occurred. It does not include the condition that might cause death if it progresses to a more serious form).
- Requires or prolongs inpatient hospitalization

Hospitalization is defined as an overnight stay at the hospital or emergency ward for observation and/or treatment that would not have been appropriate in the outpatient setting.

Complications occurring during hospitalization are AEs, and will be defined as SAEs if they cause prolongation of the current hospitalization.

When it is uncertain whether a subject needs to be "hospitalized" for an AE, the AE should also be considered an SAE.

Hospitalization for elective treatment or a preexisting non-worsening condition is not considered an AE.

Note: The following hospitalizations should not be reported as SAEs:

- Hospitalization for temporary care, e.g., when the illness does not meet the criteria for hospitalization and the hospitalization is necessitated by the need for temporary care.
- Hospitalization for social reasons, e.g., for convenience of care.
- Planned hospitalizations for protocol procedures, e.g., for the use of the study treatment or laboratory tests required by the protocol.
- Planned hospitalizations for treatment, elective surgical procedures, or examination for a pre-existing condition before signing the informed consent (in which case the condition requiring hospitalization does not worsen or develop into a new disease after the administration of the study drug, with the original documentation)
- Hospitalization for routine maintenance (e.g., battery replacement) of devices implanted before participation in the study
- results in persistent or significant disability/incapacity:

The term “disability” means a substantial disruption of a person’s ability to conduct normal life functions. This definition does not include minor clinical significance events, such as uncomplicated headache, nausea, vomiting, diarrhea, influenza, or accidental trauma (e.g., sprained ankle), which may affect daily functions but do not result in a substantial disruption.

- Results in a congenital anomaly/congenital disability.
- Other important medical events

Medical and scientific judgment is required to decide if prompt notification is required in situations other than those definition for SAEs above. This may include any event that the investigator regards as serious but may not be immediately life-threatening, result in death, or require hospitalization and medical intervention to prevent one of the above outcomes.

### Suspected Unexpected Serious Adverse Reactions

Suspected unexpected serious adverse reactions (SUSARs) are those whose nature and severity of clinical manifestations go beyond the existing information, such as the investigator’s brochure, the package insert, or the summary of product characteristics for the marketed drug.

### Management, Collection, and Recording of Adverse Events

#### Management of Adverse Events

**CRS Management**

This protocol will follow the recommendations and management for CRS, as defined by Lee et al. (2014) [37].

For patients highly suspected of CRS, early and timely intervention and monitoring evaluation should be carried out. CRS grading will be performed twice daily, and the evaluation frequency should be increased if the disease condition changes.

Mild CRS is only manifested as transient fever, fatigue, muscular pain, nausea, etc., and can often be relieved automatically. Symptomatic supportive treatment should be given. Moderate to severe CRS can be manifested as respiratory difficulty, progressive hypotension, capillary leak syndrome, acute renal failure, and other multiple organ failure and neuropsychiatric abnormalities (including convulsions and epilepsy). The critical points of treatment for moderate to severe CRS are early identification and timely treatment. The early manifestations of moderate to severe CRS are sensitive to tocilizumab and/or glucocorticoids, while the late manifestations are tolerant to tocilizumab and/or glucocorticoids. Therefore, the essence of CRS is immunosuppressive therapy, and it is essential to control the progression of CRS in time and effectively. The recommendations in Appendix 2 will help to manage CRS.

If the above treatments fail to relieve the symptoms, or IL-6 is >500 to 1000 pg/mL within three days, or IL-6 rises sharply within one day, or high fever is not reduced, or symptoms continue to worsen, tocilizumab and glucocorticoids should be applied together regardless of the CRS classification, and glucocorticoids can be used until the symptoms are relieved. If ferritin is >20 000 μg/L, high fever persists, and symptoms and signs continue to worsen in a patient, the patient should be treated with both tocilizumab and glucocorticoids, regardless of CRS grading, and glucocorticoids can be used until the symptoms are relieved. For severe capillary leak syndrome and cardiac insufficiency, continuous renal replacement treatment (CRRT) and/or plasma exchange are effective treatment measures. If the patient’s IL-6 decreases and rises sharply again during treatment, severe infections such as septicemia should be suspected. Vasopressor, mechanical ventilation, and plasma exchange are effective measures to treat severe CRS.

1) Fever: Non-steroidal anti-inflammatory drugs (such as acetaminophen and ibuprofen) and physical cooling measures are generally used for control. Infection is determined through laboratory tests and imaging examinations. If a patient develops a high fever after CT103A infusion, broad-spectrum and powerful antibiotics will be used for empirical anti-infection treatment. Meanwhile, every effort will be made to search for pathogens. The possibility of atypical pathogenic infection or reactivation should be considered when the pathogens are unclear.

2) Hypotension: Before infusion of CT103A, blood pressure, ECG, and echocardiogram tests should be conducted to determine the baseline level of blood pressure and assess cardiac function. After infusion, the investigators may make an additional assessment based on the clinical conditions. CRS-related hypotension after CT103A infusion should be treated at an early stage. The possibility of vascular leakage and pulmonary edema should be considered during volume resuscitation, and corresponding contingency plans should be made. Patients whose blood pressure cannot be maintained by active fluid infusion need vasopressor therapy. If a patient’s condition requires transfer to the intensive care unit (ICU), it is suggested that the investigator in charge and the ICU doctor collaborate to make a diagnosis and treatment.

3) Cytopenia: One or more lineages of cytopenia caused by CT103A infusion require component blood transfusion or supplement of corresponding blood cell growth factors. However, it is not suggested that cell growth factors (especially granulocyte-macrophage colony-stimulating factor [GM-CSF]) be infused in the first three weeks after the CAR-T infusion or be infused before CRS is resolved. If neutropenia occurs after CT103A infusion, granulocyte stimulating factors can be used, and patients are advised to be transferred to laminar flow wards to avoid infection. If hemoglobin is lower than 80 g/L or platelets are lower than 20 × 10^9^/L, a component blood transfusion will be required. The number of platelets should be closely monitored and maintained at 20 × 10^9^/L, or a higher level if there is active bleeding.

4) Coagulation dysfunction: It is mainly manifested as interspersed petechiae, thrombosis, and abnormal laboratory indexes, such as thrombocytopenia, an increase of D-dimer, decrease of fibrinogen, increase of fibrin degradation products, the extension of activated partial thromboplastin time, etc. Regular monitoring of CBC and coagulation indexes is helpful for the timely detection of coagulation abnormalities. Early and correct treatment can prevent the occurrence of disseminated intravascular coagulation. The main treatment measures include supplementation of platelets and coagulation factors, appropriate application of anticoagulant or antifibrinolytic drugs, etc. The fresh frozen plasma may be infused if the APTT is prolonged for more than 1.5 times the normal value or if the patient has a bleeding tendency. When fibrinogen is <1.0 g/L, blood products such as cryoprecipitate and/or fibrinogen should be infused to make fibrinogen >1.0 g/L. If there is active hemorrhage, it needs to be infused to a higher level.

Symptoms and signs of CRS in patients should be closely monitored. The diagnosis of CRS requires the exclusion of other systemic inflammatory reactions, including concurrent infection.

In general, the principles of handling CRS are as follows:

1. Severity grading for CRS

2. Different treatment measures are adopted for CRS of different grades

3. For high-risk patients with severe CRS, tocilizumab and glucocorticoids should be administered simultaneously regardless of classification, and glucocorticoids can be used until the symptoms are relieved

4. For severe capillary leak syndrome and cardiac insufficiency, CRRT and/or plasma exchange are used as effective treatment measures

5. For CRS, attention should be paid to the possibility of concurrent severe infection

**Immune Effector cell-associated neurotoxicity syndrome (ICANS) Management**

1) Carry out nervous system evaluation twice daily, and increase the evaluation frequency when the condition changes. Use the CARTOX-10 grading system for the patients

2) If necessary, the cell number and cytokine level of CT103A in cerebrospinal fluid can be monitored, and cranial MRI/CT can exclude other factors that may cause neurological abnormalities to identify whether neurotoxicity is caused by CRS that needs appropriate treatment

3) For ICANS of Grade 1, mainly supportive treatment is given. The head of the bed should be raised by at least 30 degrees to reduce the risk of aspiration and increase the cerebral venous blood supply. Anti-IL-6 therapy is recommended for patients with ICANS of Grade 1 or above complicated by CRS. For patients with ICANS of Grade 2 and above not complicated by CRS, corticosteroid therapy is preferred, and the dose can be gradually reduced when ICANS improves to Grade 1. During the dose reduction of corticosteroids, the recurrence of neurotoxicity symptoms in patients should be closely monitored. Patients with ICANS of Grade 3 accompanied by increased intracranial pressure should be treated with corticosteroids in time. The neurology department should be requested to assist in the treatment in time. Patients with ICANS of Grade 4 complicated by encephaledema should be treated with high doses of corticosteroids, oxygen inhalation, and dehydration.

For the severity grading and clinical intervention measures for neurotoxicity that occurs in this study, refer to the consensus grading for immune effector cell-related CRS and neurotoxicity issued by 2019 ASTCT and the guidelines for evaluation and management of cytotoxic therapy issued by the University of Texas MD Anderson Cancer Center in 2017.

#### Collection of Adverse Events

**Starting point for collection and recording of AE/SAEs:** All AEs/SAEs will be collected and recorded from the time of signing the ICF. Results from the Screening period will not be considered AEs unless they are caused by a study procedure.

**End points for collection and recording of AEs/SAEs:**

- All AEs/SAEs will be collected and recorded within one year after the patient's out-of-study visit or cell infusion, whichever occurs first. If early withdrawal occurs within three months after the CT103A cell infusion, AEs should be collected up to three months after the cell infusion, with the cooperation of the patients.
- AEs/SAEs occurring one year after the patient completes the end-of-study or cell infusion should also be collected if the investigator considers it related to the study treatment.

#### Recording of Adverse Events

During the study, patients are advised to report any AEs. And the trained staff are required to inquire about any AE in a non-inductive manner regularly. The investigator should report all AEs directly observed or reported by patients in concise language.

It is the responsibility of the investigator to review all laboratory results for all patients and to confirm whether they meet the criteria for AEs. Medical and scientific judgment is required in deciding whether an abnormal laboratory result should be recorded as an AE.

If any abnormal laboratory findings (e.g., blood biochemical indexes or CBC) or other abnormal assessments (e.g., ECG or vital signs) that is known to be unrelated to the indication of the study are determined by the investigator to be clinically significant, they must be recorded as AEs if they meet the definition of AE, or be recorded as SAEs if they meet the definition of SAE.

All AEs occurring during the study should be recorded in the source documentation, including a description of the AE, date of the AE onset and resolution, severity, and investigator’s judgment on the relationship between the medications of lymphodepleting conditioning/study treatment and the event, any action taken and the outcome of the AE, and their relationship with the study treatment. All AEs should be evaluated based on the comprehensive consideration of complications and concomitant medications. CRS and ICANS will be separately recorded as AEs. Abnormalities in inflammatory markers associated with CRS will not be recorded as AEs.

In addition, the following information will be collected for SAEs: Date of AEs upgrading to SAEs, date of the investigator acknowledging of AEs upgrading to SAEs, the reason for AEs upgrading to SAEs, dates of hospitalization and discharge, possible cause of death, date of death, autopsy findings, assessment of the causal relationship of SAEs to study procedures, event descriptions, etc.

### Reporting of Serious Adverse Events

Any SAE during the study, whether related to the study treatment or not, needs appropriate measures to ensure the safety of the patient. The investigator should report SAEs to the sponsor within 24 hours of being informed.

Upon receipt of the safety information from the sponsor, the investigator should read it promptly, consider any corresponding adjustment to the patient’s treatment, and communicate with the patient as soon as possible.

The investigator should follow up with SAEs as per the protocol and provide a detailed and written follow-up report within 24 hours of receiving the follow-up information in the same manner as described above.

The sponsor or its designee should evaluate and report SAEs (including SUSARs) following the latest applicable regulatory requirements for clinical studies.

### Grading of Adverse Events

Apart from CRS and neurotoxicity (ICANS), AEs should be recorded in accordance with the National Cancer Institute (NCI)-Common Terminology Criteria for Adverse Reactions (CTCAE) version 5.0. Grading of CRS and neurotoxicity should be recorded according to ASTCT Consensus Grading for Cytokine Release Syndrome and Neurologic Toxicity Associated with Immune Effector Cells in 2019.

### Relationship to the study treatment

The investigator should assess any possible associations between the AE and the study drug according to the criteria below:

| Definitely unrelated | No medication is used, or there is no correlation between the medication and the time of onset of the adverse event, or there is another clear cause of the adverse event. |
| --- | --- |
| Possibly unrelated | The adverse event is not closely related to the time of medication, the clinical manifestations are not consistent with the known adverse reactions of the drug, or the progression of the underlying disease may have similar clinical manifestations. |
| Possibly related | The medication is closely related to the time of onset of an adverse event and is supported by relevant literature; however, there is more than one drug leading to an adverse event, or the progression of the underlying disease cannot be ruled out. |
| Probably related | No history of repeated medication. The rest are the same as “definitely related,” or although there are concomitant medications, the possibility of adverse reactions caused by the concomitant medications could basically be ruled out. |
| Definitely related | There is a reasonable temporal relationship between the medication and the onset of adverse events; the event resolves or rapidly improves after the drug discontinuation (ie, positive de-challenge); adverse events re-occur due to the repeated medication (ie, positive re-challenge) and may be significantly aggravated; it is also supported by the investigator’s brochure or literature; and other confounding factors, such as underlying diseases, have been excluded. |

If the investigator cannot assess the relationship between the AE and the study treatment, the AE should also be considered a drug-induced adverse reaction.

### Follow-up of Adverse Events/Serious Adverse Events

The investigator should follow up with each AE. All SAEs and related AEs that are ongoing at the time of the AE/SAE collection and recording end point should be followed up until:

- The event resolves or returns to the baseline status or stabilizes
- Further improvement of the event is not expected as determined by the investigator
- The patient receives a new treatment regimen
- When additional information is not applicable (death or the patient refuses to provide further information, or there is evidence that the patient is lost to follow-up after every effort has been made).

During the study, the resolution time of AEs (date) should be recorded in the source document to facilitate source data verification.

For SAEs, AEs of special interest, and pregnancy events, the sponsor or designee may obtain additional patient information by telephone, fax, email, and/or monitoring for a complete medical evaluation of these reported cases.

### Outcome of Adverse Events

The investigator should determine the outcome of each AE. AE outcomes are as follows will:

Recovery: The patient recovers completely from an AE without any remaining effects or injuries.

Return to baseline: The patient recovered from an AE to the baseline values in this study.

Improving: Signs and symptoms associated with the event have been alleviated but have not completely disappeared.

Recovered with sequelae: The patient recovered but with residual effects or injuries. These residual effects may be temporary but still present at the time of reporting. If the sequelae are not considered permanent, additional information will be required at the time of follow-up when the event changes.

Ongoing: The signs and symptoms associated with the event don’t alleviate, and the patient’s condition remains unchanged; the AE is considered continuous if the patient dies due to other AEs other than this AE.

Worsening: The signs and symptoms associated with the event don’t alleviate, and the patient’s condition worsens.

Death: Only SAEs that lead to death could have “death” as the outcome. All other AEs/SAEs present at the time of death should be reported.

Unknown: When the patient is lost to follow-up and the investigator cannot assess the outcome.

### Disease Progression

Disease progression is defined as the worsening of the patient’s condition caused by the disease treated by the study drug. Disease progression may be an increase in the severity of the disease and/or an aggravate in the symptoms. The disease progression under the study will be assessed according to IMWG criteria. Events due to disease progression during the study should not be reported as AEs or SAEs.

In studies that death is the endpoint due to disease progression, the date and cause of death should be recorded in the eCRF and should not be reported as an SAE.

### New Cancer

Newly emerging cancers should be considered SAEs. New primary cancers are those cancers that are not the aim of the study treatment and are identified after enrollment in the study.

## ECOG Physical Capacity Score

See Appendix 1 for ECOG performance status score.

The time points of ECOG performance status are: Screening and enrollment period (-8 w to -18 d), baseline examination (7 days before the lymphodepleting conditioning), -1 d, 1 d, 4 d, 7 d, 10 d, 14 d, 21 d, 28 d, 2 m, 3 m, and once every three months thereafter until the out of study visit or one year after cell infusion (whichever occurs first).

## Laboratory Tests (for safety assessment)

Laboratory tests include the following:

**CBC**

Parameters include red blood cell count, hemoglobin content, hematocrit, white blood cell count, platelet count, and white blood cell classification (including ratio and absolute count of neutrophils/eosinophils/basophils, lymphocytes/monocytes).

**Blood biochemical indexes**

Parameters include alanine aminotransaminase (ALT), aspartate aminotransferase (AST), gamma-glutamyl transpeptidase (γ-GT), total bilirubin (TBIL), direct bilirubin (DBIL), alkaline phosphatase (AKP or ALP), glucose (Glu), total protein (TP), albumin (ALB), globulin (G), albumin/globulin ratio (A/G) lactate dehydrogenase (LDH), urea/urea nitrogen (Urea/BUN), creatinine (CRE), uric acid (URIC), creatine kinase (CK), creatine kinase isoenzymes (CK-MB), troponin, sodium (Na), potassium (K), magnesium (Mg), chloride (Cl), and phosphorus (P).

**Urinalysis:**

Parameters include pH, urine leucocytes, urine protein, urine erythrocytes, glucose in the urine, ketone bodies, bilirubin, urobilinogen, nitrite, and leukocyte esterase; microscopy should be performed if urine dipstick is abnormal, including urine sediment, red blood cell count, white blood cell count, epithelial cells, crystals, cell cast, and bacteria, only when urine protein is ≥+ and not related to disease, the test is suggested.

**Coagulation**

Parameters include plasma prothrombin time (PT), APTT, thrombin time (TT), fibrinogen (FIB), D-dimer, and international normalized ratio (INR).

## Vital Signs

Vital sign measurements include axillary temperature, heart rate, respiratory rate, and systolic and diastolic blood pressure at rest.

## Physical Examination

Physical examination includes at least skin, mucous membranes, lymph nodes, head, neck, chest, abdomen, spine/extremities, and nervous system.

## Electrocardiogram and Echocardiogram

During CT103A cell therapy and follow-up observation period, ECG or echocardiogram can be performed at the discretion of the investigator according to the patient's symptoms and signs of suspected abnormal cardiac function.

# EFFICACY ASSESSMENTS

## Efficacy Evaluation Criteria

MM refers to the efficacy criteria for myeloma (Appendix 3) and MRD efficacy criteria (Appendix 4) of the IMWG in 2016.

## Effective Assessment Indicators

- PFS: the time from the CT103A infusion to disease progression or death from any cause.
- Best ORR: best ORR at one month, three months, and six months after cell infusion, including sCR, CR, VGPR, PR.
- OS: the time from the CT103A infusion to death for any reason.
- DOR: the time from the first evaluation of PR or better, to the initial assessment of disease progression or death from any cause.
- MRD response assessment: the MRD assessment includes the proportion of MRD negative and the duration of MRD negative.

PFS, best ORR, and DOR are evaluated based on the response assessed by the investigator.

# PHARMACOKINETICS

## Pharmacokinetics Evaluation Indicators

The number of CAR-T cells and VCN amplified by CT103A in peripheral blood after receiving cell infusion. PK parameters are as follows:

- C_max_: maximum concentration of CT103A amplified in peripheral blood after administration.
- T_max_: time to maximum plasma concentration.
- AUC_0-28d_ and AUC_0-90d_: area under the curve of PK test time points at 28 and 90 days after infusion.
- The plasma concentration of Selinexor in peripheral blood.

## Pharmacokinetic Blood Sample Test

After all specimens are collected and sent to the laboratory, the laboratory will perform PK testing using the validated methods.

# PHARMACODYNAMICS

## Pharmacodynamic Evaluation Indicators

The concentration of CAR-T-related inflammatory factors (e.g., IL-6, ferritin) at each time point.

## Pharmacodynamic blood sample Test

After all specimens are collected and sent to the laboratory, the laboratory will perform PD testing using the validated methods.

# QUALITY OF LIFE ASSESSMENT

The quality of life of patients will be assessed using European Organisation for Research and Treatment of Cancer Quality of Life Questionnaire-C30 (EORTC-QLQ-C30) and the European Organization for Research and Treatment of Cancer Quality of Life Questionnaire Multiple Myeloma Module (EORTC-QLQ-MY20).

# IMMUNE STATUS OF THE PATIENTS

The changes in lymphocyte subsets, immunoglobulin, and infectious status after receiving the treatment will be collected.

# STATISTICAL ANALYSIS

Detailed statistical analysis methods will be described in the statistical analysis plan (SAP). If there is any inconsistency between the SAP and the protocol, except that the primary study endpoint and changes in the primary analysis method must be reflected in the protocol simultaneously, the rest will only be listed, described, and clarified in the SAP.

## General Methods

PK parameters will be calculated based on WinNonlin version 6.1 or above. All remaining statistical analyses will be performed based on SAS^®^ version 9.2 or above.

Unless otherwise specified, summary tables will be produced for the scheduled time points. Listings will be produced by patient number, time points, and other variables for unscheduled time points.

Continuous variables, such as age, will be statistically described using the value of observations, mean, median, quartile, standard deviation, minimum, and maximum; categorical variables will be statistically described by the number of cases and the percentage in each category. For the PK parameters, such as AUC and C_max_, geometric mean and geometric coefficient of variation will be calculated. Time-event data will be estimated by the Kaplan-Meier method for the median time and 95% CIs for the population. The decimal places of the minimum and maximum values are consistent with the record on the eCRF; the number of decimal places for the mean and median values is 1 more than the original data; the number of decimal places for the standard deviation is 2 more than the original data, and the percentage is retained to 2 decimal places. The number of decimal places reserved for all statistics is generally not more than 4. Except for the patient distribution, statistical summary tables and charts will be based on the corresponding analysis set, the relevant data of all patients will be listed.

## Sample Size

The study plans to enroll 16 to 20 patients.

## Analysis Populations

The main statistical analysis sets are as follows:

Screening Analysis Set: Patients who signed the informed consent will be included in the screening analysis set.

Enrollment Analysis Set (EAS): Patients in the screening analysis set who meet the inclusion criteria but did not meet the exclusion criteria and who are assigned an enrollment number will be enrolled in EAS.

Mononuclear cell collection Analysis set: Patients who undergo mononuclear cell collection in the enrollment analysis set will be included in this analysis set.

Lymphodepleting conditioning Analysis set: Patients who undergo lymphodepleting conditioning in the enrollment analysis set will be included in the lymphodepleting conditioning analysis set.

Full Analysis Set (FAS): All patients who have used CT103A for infusion will be included in this analysis set. FAS is the primary analysis set for efficacy endpoints.

Per-protocol Analysis Set (PPS): A subset of FAS, including all patients with no missing primary efficacy measures and no significant protocol deviation which will affect the efficacy.

Safety Set (SS): All the patients who receive cell infusion and have at least one safety evaluation after the infusion are included in the SS. The safety analysis set is used to analyze safety measures.

Pharmacokinetic Analysis Set (PKS): All patients who have used the study treatment and have at least one post-infusion PK evaluation data (concentration/parameter) will be included in the PKS.

Pharmacodynamics Analysis Set (PDS): All patients who have used the study treatment and have at least one post-infusion PD evaluation data will be included in the PDS.

## Handling of Missing Values

Efficacy measures: All the missing values of the primary efficacy measures caused by early withdrawal are regarded as “unevaluable” in the analysis. The censoring rules for time-event variables are detailed in the SAP.

Unless otherwise specified, there will be an imputation for the missing value for the baseline, safety data, and PK data.

The extreme value of laboratory data caused by improper handling of specimens, blood samples, etc., will be analyzed using the corresponding data from unscheduled visits during the analysis.

## Statistical Methodology

The baseline value of demographic and other baseline characteristic values and safety measures are the conditions when the patients are enrolled in the study. The baseline value of efficacy and relevant measures are the value during the baseline period before the lymphodepleting conditioning.

### Patient Distribution

The number of cases (percentage) is used to describe the enrollment and completion of the patients. The distribution of subjects in each dataset and the reasons for the withdrawal of dropout and patient exclusion will be listed.

### Demographic Data and Baseline Analysis

Descriptive statistical demographic data and other baseline characteristic values will be presented. Continuous variables are used to calculate the number of cases, mean, standard deviation, median, minimum, and maximum of non-missing observation results; counting and ranked data are used to calculate frequency and composition ratio.

### Analysis of Safety Measures

**AE analysis**

According to the time of AE, the AE analysis includes three stages: PBMC collection to lymphodepleting preconditioning, lymphodepleting conditioning to infusion, and after infusion; and the AE that occurred after cell infusion will be mainly analyzed.

The Systematic Organ Classification (SOC) and Preferred Term (PT) classifications of AEs will be coded using the latest version of the Medical Dictionary of Regulated Activities (MedDRA). The number and percentage of AE, SAE, AESI after cell infusion will be summarized by group, AE, SOC, and PT, with a listing of related AEs. In addition, the severity of AEs after cell infusion (NCI-CTCAE version 5.0) will be summarized by group, SOC, and PT.

CRS and ICANS will be summarized and analyzed according to the severity level (2019 ASTCT grading criteria).

**Laboratory analysis**

Laboratory test data will be graded according to CTCAE version 5.0. If the CTCAE classification cannot be determined, the results will be classified as Low, Normal, and High according to the normal range of the laboratory. Laboratory test results, such as CBC, blood biochemical indexes, coagulation, urinalysis, inflammation factors, blood oxygen saturation test, and its changes before and after the baseline value, will be summarized by different doses of each visit. The abnormal changes in CBC, blood biochemical indexes, coagulation, urinalysis, and other parameters before and after the baseline will be summarized, and the percentage of the greatest changes in the post-baseline visit will be summarized.

All abnormal laboratory changes will be listed.

**Analysis of vital signs, physical examination, ECG, ECOG performance score, echocardiogram, etc**

Descriptive statistics of the data at each visit and the changes from baseline will be summarized by different dose groups, and the abnormal values will be listed. Statistical analysis methods are detailed in the SAP.

### Efficacy Analysis

PFS, DOR, and OS: The median of PFS, DOR, and OS will be calculated, and the two-sided 95% CIs will be estimated by the Brookmeyer and Crowley method. Kaplan-Meier survival curves will be plotted. Survival rates and 95% CIs at 6, 12, 18, and 24 months will be calculated.

Best ORR: Point estimates of the best ORR (sCR>CR>VGPR>PR) at 1, 3, and 6 months will be calculated, and the two-sided 95% CIs will be estimated using the Clopper-Pearson exact method.

MRD efficacy assessment: The proportion of patients with MRD negative and the duration of MRD negative will be calculated.

### PK/PD Analysis

PK/PD analyses will be performed using the PK and PD analysis sets, respectively.

Descriptive statistics will be used to summarize the drug concentration data at each time point, with individual blood drug concentration-time curves and arithmetic mean concentration-time plots presented on the linear and semi-logarithmic scales for the treatment groups. According to the actual blood collection time, WinNonlin software will be used to calculate PK parameters through the non-compartment model.

Main PK parameters: C_max_ of CT103A cells and VCN amplified in peripheral blood after CT103A infusion, T_max_, AUC_0-28d_, AUC_0-90d_, etc. Descriptive statistics of PK parameters (CT103A cell concentration and lentiviral VCN, mainly lentiviral copy number) will be summarized and analyzed. The serum concentration of Selinexor in peripheral blood after infusion will be analyzed.

PD analysis: cytokines

Descriptive statistics will be used to analyze CAR-T-related inflammatory factors (IL-6 and ferritin concentration levels) at each time point. The changes from baseline and line charts will be used to show the trends. The exploratory analysis of the correlation between inflammatory factors (IL-6, ferritin) and safety and efficacy will be based on the CRS grade and the results of the IMWG efficacy evaluation. The concentration of inflammatory factors at the corresponding visit will be analyzed.

### Exploratory Analysis

The effect of different doses of Selinexor on the CT103A.

## Summary and Analysis

The summarized analysis will be performed one year after the completion of cell infusion of the last patient in this study, with descriptive analyses for endpoints, including efficacy, safety, and PK parameters.

# STUDY MANAGEMENT

## Data Handling and Record Keeping

The data management for this study will be conducted according to China's applicable laws and regulations.

### Data Collection

Study data will be collected using eCRFs (excel format). Study data (except for PK data) will be entered into the eCRF by the investigator or authorized site staff. Before the site initiation or data entry, the investigators and authorized site staff will be appropriately trained, and appropriate information security measures will be implemented.

All data entered are in simplified Chinese. The eCRF should be completed as soon as possible during or after the visits, and updated at any time to ensure it reflects the latest conditions for the patients participating in the study. To avoid differences in result evaluations between investigators, effort should be made to ensure that the same person completes the baseline evaluation and all subsequent efficacy and safety evaluations for the same patient. The investigator must review the data to ensure the accuracy and correctness of all data entered into the eCRF. If certain assessments are not performed during the study, or certain information is unavailable, not applicable, or unknown, the investigator should record it in the eCRF.

Unless otherwise specified, the eCRF will only be used as a form for collecting data and will not be used as source data. Source documents refer to any record used by the investigator or study site that are relevant to the patient and can demonstrate the patient’s presence, enrollment criteria, and participation in this study, including laboratory records, ECG results, patient folders, etc.

The investigator is responsible for maintaining all source documents and will ensure the documents are monitored by the clinical research associate (CRA) at each visit. In addition, the investigator must submit a complete eCRF for each patient participating in the study, regardless of the duration of their participation. All supporting documents (such as laboratory or research center records) submitted with the eCRF should be carefully verified for study numbers and patient numbers, and all personal privacy information (including patient names) should be deleted or rendered illegible to protect the patient’s privacy.

### Data Monitoring

The CRA will review the original documents and eCRF and assess the completeness and consistency. Then CRA will compare the eCRF with the original documents and other relevant documents to ensure the integrity and consistency of the critical data.

### Data Entry and Modification

All data entry, correction, and modification will be the responsibility of the investigator or the authorized personnel, and the CRA does not have this authority. The roles and authority of the center staff responsible for data entry will be determined in advance. If there is any question about the data, the CRA or the data manager will issue the query, and the center staff will answer the query.

### Database Lock

The principal investigator, the sponsor, the head of statistical analysis, and the head of data management will hold the data review meeting and jointly determine the data set population. Then the data manager will lock the database.

### Data Transfer

After the database is locked, it will be submitted to the statistical analyst for analysis according to the requirements of the statistical analysis plan. After completing the statistical analysis, the analyst will develop the report, and the principal investigator is responsible for the clinical study report.

### Data Preservation

During the study and upon the study completion, the study center/sponsor will archive the necessary documents as per the requirements of the Good Clinical Practice (GCP) in China.

In addition, these documents will be kept until:

- At least five years after study completion or early termination, or
- At least five years after the final approval of the application in China and until no pending or pending marketing applications in China, or
- At least five years after the formal termination of the clinical development project for the investigational drug.

Whichever is longer.

The sponsor shall promptly notify the investigator of any documents that are not required to be retained further. These documents should not be destroyed/moved without informing and obtaining consent from the sponsor.

## Ethical Considerations and Informed Consent

Before initiating the screening procedure, patients will be informed orally and in writing by the investigator of the nature, purpose, and risks of the screening procedure, as well as the study objectives, methods, limitations, obligations, benefits, risks, rewards, insurance coverage, and possible adverse drug reactions. Written informed consent will be provided, and the process of signing the informed consent will be performed for each patient before enrollment in the study. The notice to the Subject and the informed consent must be signed and dated. The informed consent must be duplicated, with one copy given to the patient and one original copy retained by the investigator as part of the clinical study record. No study-specific procedures will be performed until obtaining written consent. The time to obtain the informed consent must be recorded in the EDC system. The informed consent form must be maintained in the trial investigator folder.

If protocol amendment is required, the patient note and informed consent may be revised to reflect the changes in the protocol. Revisions to the patient notice and informed consent must be submitted to the independent ethics committee (IEC) for approval, after which all patients enrolled in the study and those currently on the study must sign the revised patient note and informed consent approved by the IEC.

The preservation of study results should be consistent with the information protection laws of China. Patients should be informed that auditors, inspectors, ethics committees, and drug regulatory inspectors can access the patients' original medical records to verify the process and data of the clinical trials without violating confidentiality and related laws and regulations.

The investigator will maintain a patient identification code sheet (patient number and corresponding patient name) to identify the appropriate records.

## Quality control

### Inspection

This study will be monitored according to the GCP of China. Inspections will be conducted in accordance with all relevant legal and regulatory requirements. The capacity, facilities, and technical equipment needed in the study will be assessed. The study center will undergo periodic monitoring visits when the patient is enrolled. At the same time, the data entered into the eCRF will be compared with the original data, and the investigator will be informed of any errors or omissions. Compliance with the study protocol will also be ensured at the study center, the supply of study drugs will be arranged, and the study drugs must be preserved in appropriate conditions.

### Data Verification

This study requires that the inspectors directly access the original data for verification by comparing data on patients' eCRF with their sources. This data verification process is essential to ensure the study's quality when inspectors can point out the errors and omissions during data input.

### Audit and Inspection

The sponsor's representative, regulatory authorities, IEC, etc., can go to the study center for inspection, including original data verification.

### Personnel Training

The principal investigator will keep a copy of all study participants' assignments and training records. The investigators will ensure that all personnel has been appropriately trained and authorized with the study and that any information relevant to the conduct of the study has been passed on to the relevant personnel.

## Protocol Approval and Amendments

Before the initiation of the study, the protocol and other relevant documents are submitted to the IEC/competent authority for approval as per local legal requirements. Sponsors and investigators must ensure that all ethical and legal requirements are met before enrolling the first patient.

The study protocol must be strictly followed during the study. Changes to the protocol must be made in writing, issued by the responsible person, and approved by the IEC/competent authority (if applicable) before implementation.

## Duration of Clinical Study

The main follow-up period of this study is one year after cell infusion.

## Confidential Data Protection

The identity of the patients participating in the study must be confidential. The patient shall be represented only by his/her number, initials, or date of birth on the eCRF and other documents, and his/her name shall not appear. The investigator must keep documentation identifying the patient (eg, signed patient note and informed consent forms) confidential.

## Financing and Insurance

Coverage is set in accordance with the GCP and local insurance laws. Participants will be paid in the event of death or any impairment to the patient's health or state of life due to study participation.

The insurance policy will be saved in the trial master file.

# REFERENCES

1. van de Donk NWCJ, Pawlyn C, Yong KL. Multiple myeloma. *Lancet*. 2021;397(10272):410-427. doi:10.1016/S0140-6736(21)00135-5
2. Terpos E, Zamagni E, Lentzsch S, et al. Treatment of multiple myeloma-related bone disease: recommendations from the Bone Working Group of the International Myeloma Working Group. *Lancet Oncol.* 2021;22(3):e119-e130. doi:10.1016/S1470-2045(20)30559-3
3. Bladé J, Fernández de Larrea C, Rosiñol L, Cibeira MT, Jiménez R, Powles R. Soft-tissue plasmacytomas in multiple myeloma: incidence, mechanisms of extramedullary spread, and treatment approach. *J Clin Oncol.* 2011;29(28):3805-3812. doi:10.1200/JCO.2011.34.9290
4. Vande Broek I, Vanderkerken K, Van Camp B, Van Riet I. Extravasation and homing mechanisms in multiple myeloma. *Clin Exp Metastasis.* 2008;25(4):325-334. doi:10.1007/s10585-007-9108-4
5. Bhutani M, Foureau DM, Atrash S, Voorhees PM, Usmani SZ. Extramedullary multiple myeloma. *Leukemia*. 2020;34(1):1-20. doi:10.1038/s41375-019-0660-0
6. Sevcikova S, Minarik J, Stork M, Jelinek T, Pour L, Hajek R. Extramedullary disease in multiple myeloma - controversies and future directions. *Blood Rev*. 2019;36:32-39. doi:10.1016/j.blre.2019.04.002
7. Varga C, Xie W, Laubach J, et al. Development of extramedullary myeloma in the era of novel agents: no evidence of increased risk with lenalidomide-bortezomib combinations. *Br J Haematol.* 2015;169(6):843-850. doi:10.1111/bjh.13382
8. Pasmantier M W, Azar H A. Pasmantier MW, Azar HA. Extraskeletal spread in multiple plasma cell myeloma. A review of 57 autopsied cases. *Cancer*. 1969;23(1):167-174. doi:10.1002/1097-0142(196901)23:1<167::aid-cncr2820230122>3.0.co;2-0.
9. Varettoni M, Corso A, Pica G, Mangiacavalli S, Pascutto C, Lazzarino M. Incidence, presenting features and outcome of extramedullary disease in multiple myeloma: a longitudinal study on 1003 consecutive patients. *Ann Oncol*. 2010;21(2):325-330. doi:10.1093/annonc/mdp329
10. Mangiacavalli S, Pompa A, Ferretti V, et al. The possible role of burden of therapy on the risk of myeloma extramedullary spread. *Ann Hematol.* 2017;96(1):73-80. doi:10.1007/s00277-016-2847-z
11. Beksac M, Seval G C, Kanellias N, et al. A real world multicenter retrospective study on extramedullary disease from Balkan Myeloma Study Group and Barcelona University: analysis of parameters that improve outcome. *Haematologica.* 2020;105(1):201-208. doi:10.3324/haematol.2019.219295
12. Weinstock M, Ghobrial IM. Extramedullary multiple myeloma. *Leuk Lymphoma.* 2013;54(6):1135-1141. doi:10.3109/10428194.2012.740562
13. Deng S, Xu Y, An G, et al. Features of extramedullary disease of multiple myeloma: high frequency of p53 deletion and poor survival: a retrospective single-center study of 834 cases. *Clin Lymphoma Myeloma Leuk.* 2015;15(5):286-291. doi:10.1016/j.clml.2014.12.013
14. Rasche L, Bernard C, Topp MS, et al. Features of extramedullary myeloma relapse: high proliferation, minimal marrow involvement, adverse cytogenetics: a retrospective single-center study of 24 cases. *Ann Hematol*. 2012;91(7):1031-1037. doi:10.1007/s00277-012-1414-5
15. Muchtar E, Gatt ME, Rouvio O, et al. Efficacy and safety of salvage therapy using Carfilzomib for relapsed or refractory multiple myeloma patients: a multicentre retrospective observational study. *Br J Haematol*. 2016;172(1):89-96. doi:10.1111/bjh.13799
16. Pick M, Vainstein V, Goldschmidt N, et al. Daratumumab resistance is frequent in advanced-stage multiple myeloma patients irrespective of CD38 expression and is related to dismal prognosis. *Eur J Haematol.* 2018;100(5):494-501. doi:10.1111/ejh.13046
17. Pérez-Simón JA, Sureda A, Fernández-Aviles F, et al. Reduced-intensity conditioning allogeneic transplantation is associated with a high incidence of extramedullary relapses in multiple myeloma patients. *Leukemia.* 2006;20(3):542-545. doi:10.1038/sj.leu.2404085
18. Nachmias B, Schimmer AD. Targeting nuclear import and export in hematological malignancies. *Leukemia*. 2020;34(11):2875-2886. doi:10.1038/s41375-020-0958-y
19. Tai YT, Landesman Y, Acharya C, et al. CRM1 inhibition induces tumor cell cytotoxicity and impairs osteoclastogenesis in multiple myeloma: molecular mechanisms and therapeutic implications. *Leukemia.* 2014;28(1):155-165. doi:10.1038/leu.2013.115
20. Sun Q, Chen X, Zhou Q, Burstein E, Yang S, Jia D. Inhibiting cancer cell hallmark features through nuclear export inhibition. *Signal Transduct Target Ther*. 2016;1:16010. doi:10.1038/sigtrans.2016.10
21. Schmidt J, Braggio E, Kortuem KM, et al. Genome-wide studies in multiple myeloma identify XPO1/CRM1 as a critical target validated using the selective nuclear export inhibitor KPT-276. *Leukemia.* 2013;27(12):2357-2365. doi:10.1038/leu.2013.172
22. Gandhi UH, Senapedis W, Baloglu E, et al. Clinical Implications of Targeting XPO1-mediated Nuclear Export in Multiple Myeloma. *Clin Lymphoma Myeloma Leuk.* 2018;18(5):335-345. doi:10.1016/j.clml.2018.03.003
23. Malandrakis P, Ntanasis-Stathopoulos I, Gavriatopoulou M, Terpos E. Clinical utility of selinexor/dexamethasone in patients with relapsed or refractory multiple myeloma: a review of current evidence and patient selection. *Onco Targets Ther*. 2020;13:6405-6416. doi:10.2147/OTT.S227166
24. Nachmias B, Schimmer AD. Targeting nuclear import and export in hematological malignancies. *Leukemia.* 2020;34(11):2875-2886. doi:10.1038/s41375-020-0958-y
25. Jiménez I, Carabia J, Bobillo S, et al. Repolarization of tumor infiltrating macrophages and increased survival in mouse primary CNS lymphomas after XPO1 and BTK inhibition*. J Neurooncol.* 2020;149(1):13-25. doi:10.1007/s11060-020-03580-y
26. Brudno JN, Maric I, Hartman SD, et al. T cells genetically modified to express an anti-B-cell maturation antigen chimeric antigen receptor cause remissions of poor-prognosis relapsed multiple myeloma. *J Clin Oncol*. 2018;36(22):2267-2280. doi:10.1200/JCO.2018.77.8084
27. Zhao WH, Liu J, Wang BY, et al. A phase 1, open-label study of LCAR-B38M, a chimeric antigen receptor T cell therapy directed against B cell maturation antigen, in patients with relapsed or refractory multiple myeloma. *J Hematol Oncol*. 2018;11(1):141. doi:10.1186/s13045-018-0681-6
28. Raje N, Berdeja J, Lin Y, et al. Anti-BCMA CAR T-cell therapy bb2121 in relapsed or refractory multiple myeloma. *N Engl J Med*. 2019;380(18):1726-1737. doi:10.1056/NEJMoa1817226
29. Xu J, Chen LJ, Yang SS, et al. Exploratory trial of a biepitopic CAR T-targeting B cell maturation antigen in relapsed/refractory multiple myeloma. *Proc Natl Acad Sci U S A*. 2019;116(19):9543-9551. doi:10.1073/pnas.1819745116
30. Cohen AD, Garfall AL, Stadtmauer EA, et al. B cell maturation antigen-specific CAR T cells are clinically active in multiple myeloma. *J Clin Invest*. 2019;129(6):2210-2221. doi:10.1172/JCI126397
31. Wang D, Wang J, Hu G, et al. A phase 1 study of a novel fully human BCMA‑targeting CAR (CT103A) in patients with relapsed/refractory multiple myeloma. *Blood*. 2021;137(21):2890-2901. doi:10.1182/blood.2020008936
32. Li C, Cao W, Que Y, et al. A phase I study of anti-BCMA CAR T cell therapy in relapsed/refractory multiple myeloma and plasma cell leukemia. *Clin Transl Med*. 2021;11(3):e346. doi:10.1002/ctm2.346
33. Bahlis NJ, Sutherland H, White D, et al. Selinexor plus low-dose bortezomib and dexamethasone for patients with relapsed or refractory multiple myeloma. *Blood.* 2018;132(24):2546-2554. doi:10.1182/blood-2018-06-858852
34. Richter J, Madduri D, Richard S, Chari A. Selinexor in relapsed/refractory multiple myeloma. *Ther Adv Hematol*. 2020;11:2040620720930629. doi:10.1177/2040620720930629
35. Grosicki S, Simonova M, Spicka I, et al. Once-per-week selinexor, bortezomib, and dexamethasone versus twice-per-week bortezomib and dexamethasone in patients with multiple myeloma (BOSTON): a randomised, open-label, phase 3 trial. *Lancet*. 2020;396(10262):1563-1573. doi:10.1016/S0140-6736(20)32292-3
36. Tyler PM, Servos MM, de Vries RC, et al. Clinical dosing regimen of selinexor maintains normal immune homeostasis and T-cell effector function in mice: implications for combination with immunotherapy. *Mol Cancer Ther*. 2017;16(3):428-439. doi:10.1158/1535-7163.MCT-16-0496
37. Lee JT, Chiu AG. Topical anti-infective sinonasal irrigations: update and literature review. *Am J Rhinol Allergy*. 2014;28(1):29-38. doi:10.2500/ajra.2014.28.3988

# APPENDICES

**Appendix 1. Eastern Cooperative Oncology Group Performance Status Scale**

| Grade | ECOG Performance Status |
| --- | --- |
| 0 | Fully active, able to carry on all pre-disease performance without restriction |
| 1 | Restricted in physically strenuous activity but ambulatory and able to carry out work of a light or sedentary nature, e.g., light housework, office work |
| 2 | Ambulatory and capable of all self-care but unable to carry out any work activities; up and about more than 50% of waking hours |
| 3 | Capable of only limited self-care; confined to bed or chair more than 50% of waking hours |
| 4 | Completely disabled; cannot carry on any self-care; totally confined to bed or chair |
| 5 | Dead |

Oken MM, Creech RH, Tormey DC, et al. Toxicity and response criteria of the Eastern Cooperative Oncology Group. Am J Clin Oncol. 1982;5:649-655

**Appendix 2. Recommendations of CRS Management**

| **CRS Classification** | **Anti-IL-6 Therapy** | **Steroid Hormone^1,2^** | **Other Supportive Treatments** |
| --- | --- | --- | --- |
| Level 1 | In patients with significant symptoms and/or comorbidities, if CRS duration is prolonged (>3 days), the use of tocilizumab for Grade 2 toxicity is considered. | Not applicable | - Empirical use of broad-spectrum antibiotics; in case of neutropenia^3^, granulocyte colony-stimulating factor may be considered; - Maintain intravenous fluids for hydration; - Symptomatic treatment for organ toxicity. |
| Level 2 | Tocilizumab 8mg/kg intravenously for 1 hour (dose not exceeding 800 mg)^1^. If the symptoms do not improve, tocilizumab can be administered again after 8 hours; no more than 3 doses within 24 hours, with a maximum of 4 doses. | Anti-IL-6 treatment for persistent refractory hypotension after 1-2 doses: dexamethasone 10 mg, intravenously, q6h (or equivalent)^4^. | - Rapid intravenous pumping liquid as needed; - Refractory hypotension that persists after 2 infusion pump pushups and anti-IL-6 treatments: initiation of vasopressors, consideration for transfer to ICU, consideration for echocardiography, and initiation of other hemodynamic monitoring methods; - Management of Grade 3 toxicity if there is no improvement within 24 hours of initiation of anti-IL-6 treatment; - Symptomatic treatment for organ toxicity. |
| Level 3 | If the maximum dose of anti-IL-6 drug treatment is not reached within 24 hours, the anti-IL-6 treatment can be performed according to Grade 2 toxicity^1^. | Dexamethasone 10 mg, intravenously, q6h (or equivalent)^4^. If refractory, it will be treated as Grade 4 toxicity. | - Transfer to ICU, undergo echocardiography, and undergo hemodynamic monitoring; - Oxygen supply; - Rapid intravenous pumping and vasopressors as needed; - Symptomatic treatment for organ toxicity. |
| Level 4 | If the maximum dose of anti-IL-6 drug treatment is not reached within 24 hours, the anti-IL-6 treatment can be performed according to Grade 2 toxicity^1^. | Dexamethasone 10 mg, intravenously, q6h (or equivalent)^4^. If refractory, methylprednisolone^5^, intravenous, 1000 mg/d is considered. | - ICU monitoring and hemodynamic monitoring; - Mechanical ventilation as needed; - Rapid intravenous pumping and vasopressors as needed; - Symptomatic treatment for organ toxicity. |

Abbreviations: CRS, cytokine release syndrome; GM-CSF, granulocyte-macrophage colony‑stimulating factor; HLH, hemophagocytic lymphohistiocytosis; ICU, intensive care unit; IL‑6, interleukin-6; MAS, macrophage activation syndrome; q6h, once every 6 hours; q12h, once every 12 hours.

Note: HLH/MAS occurring during CRS should be treated as CRS with the addition of steroids. If symptoms do not improve within 48 hours, etoposide with intrathecal cytarabine may be considered for the treatment of neurotoxicity.

1. Assess the need for subsequent dosing after each dose.
2. Antifungal prophylaxis should be strongly considered in patients receiving steroid therapy for CRS and/or central neurotoxicity.
3. GM-CSF is not recommended for use with CAR-T therapy.
4. Other steroids with equivalent doses may be considered alternatively.
5. Methylprednisolone, intravenously, 1000 mg/ day × 3 days, then tapering to 250 mg q12h × 2 days to 125 mg q12h × 2 days to 60 mg q12h × 2 days.

**Appendix 3. International Myeloma Working Group Consensus Criteria for Response Assessment in Multiple Myeloma**

| Response Classification | Response Criteria^a^ |
| --- | --- |
| Stringent Complete Response (sCR) | CR, as defined below, plus the following:  Normal FLC ratio^b^ and absence of clonal cells in bone marrow biopsy by immunohistochemistry(κ/λ ratio ≤ 4:1 or ≥ 1:2 for κ and λ patients, respectively, after counting ≥100 plasma cells)^c^ |
| Complete Response (CR) | Negative immunofixation on the serum and urine and disappearance of any soft tissue plasmacytomas and < 5% plasma cells in bone marrow aspirates |
| Very Good Partial Response (VGPR) | Serum and urine M-protein detectable by immunofixation but not on electrophoresis or ≥ 90% reduction in serum M-protein plus urine M-protein level < 100 mg per 24 h |
| Partial Response (PR) | ≥ 50% reduction of serum M-protein and reduction in 24-hour urinary M-protein by ≥ 90% or to <200 mg per 24 hour.  If serum and urine M-protein is unmeasurable, a ≥ 50% decrease in the difference between involved and uninvolved FLC levels is required in place of the M-protein criteria.  In addition to the above criteria, if present at baseline, ≥ 50% reduction in the size (SPD) ^d^ of soft tissue plasmacytomas is also required |
| Minimal Response (MR) | 25-49% reduction of serum M-protein and reduction in 24-hour urine M-protein by 50-89%, which still exceeds 200 mg per 24 hours. In addition to the above-listed criteria, if present at baseline, 25-49% reduction in the size of soft tissue plasmacytomas is also required. No increase in the size or number of lytic bone lesions (development of compression fracture does not exclude response). |
| Stable Disease (SD) | Not meeting criteria for CR, VGPR, PR, MR, or progression |
| Progressive Disease^e,f^ | Any of the following:  -Increase of 25% from lowest response value in any one or more of the following:  1) Serum M-component (absolute increase must be ≥ 0.5 g/dL).  2) Serum M-protein increase ≥1 g/dL, if the lowest M component was ≥5 g/dL.  3) Urine M-component (absolute increase must be ≥ 200 mg per 24 h).  -In patients without measurable serum and urine M-protein levels, the difference between involved and uninvolved FLC levels (absolute increase must be >10 mg/dL).  -In patients without measurable serum and urine M-protein levels and without measurable involved FLC levels, bone marrow plasma-cell percentage irrespective of baseline status (absolute increase must be ≥10%).  -Appearance of a new lesion(s), ≥50% increase from nadir in SPD of >1 lesion, or ≥50% increase in the longest diameter of a previous lesion >1 cm in short axis.  ≥50% increase in circulating plasma cells (minimum of 200 cells per μL) if this is the only measure of disease. |
| Clinical Relapse | Clinical relapse requires one or more of the following criteria:  -Direct indicators of increasing disease and/or end organ dysfunction (CRAB features) related to the underlying clonal plasma-cell proliferative disorder. It is not used in calculation of time to progression or progression-free survival but is listed as something that can be reported optionally or for use in clinical practice.  -Development of new soft tissue plasmacytomas or bone lesions (osteoporotic fractures do not constitute progression).  -Definite increase in the size of existing plasmacytomas or bone lesions. A definite increase is defined as a 50% (and ≥1 cm) increase as measured serially by the SPD of the measurable lesion.  -Hypercalcaemia (>11 mg/dL).  -Decrease in haemoglobin of ≥2 g/dL not related to therapy or other non-myeloma-related conditions.  -Rise in serum creatinine by 2 mg/dL or more from the start of the therapy and attributable to myeloma.  -Hyperviscosity related to serum paraprotein |
| Relapse From Complete Response (to be Used Only if the End Point is Disease-Free Survival) | Any one or more of the following criteria:  -Reappearance of serum or urine M-protein by immunofixation or electrophoresis.  -Development of ≥5% plasma cells in the bone marrow.  -Appearance of any other sign of progression (i.e., new plasmacytoma, lytic bone lesion, or hypercalcemia see above) |

a. All response categories require two consecutive assessments made any time before starting any new therapy; for MRD there is no need for two consecutive assessments, but information on MRD after each treatment stage is recommended (e.g., after induction, high-dose therapy/ASCT, consolidation, maintenance). MRD tests should be initiated only at the time of suspected complete response.

All categories of response and MRD require no known evidence of progressive or new bone lesions if radiographic studies were performed. However, radiographic studies are not required to satisfy these response requirements except for the requirement of FDG PET if imaging MRD-negative status is reported.

Derived from international uniform response criteria for multiple myeloma. Minor response definitions and clarifications derived from Rajkumar and colleagues. When the only method to measure disease is by serum FLC levels: complete response can be defined as a normal FLC ratio of 0.26 to 1.65 in addition to the complete response criteria listed previously. Very good partial response in such patients requires a ≥ 90% decrease in the difference between involved and uninvolved FLC levels. All response categories require two consecutive assessments made at any time before the institution of any new therapy; all categories also require no known evidence of progressive or new bone lesions or extramedullary plasmacytomas if radiographic studies were performed. Radiographic studies are not required to satisfy these response requirements. Bone marrow assessments do not need to be confirmed. Each category, except for stable disease, will be considered unconfirmed until the confirmatory test is performed. The date of the initial test is considered as the date of response for evaluation of time dependent outcomes such as duration of response.

b. All recommendations regarding clinical uses relating to serum FLC levels or FLC ratio are based on results obtained with the validated Freelite test (Binding Site, Birmingham, UK).

c. Presence or absence of clonal cells is based upon the κ/λ ratio. An abnormal κ/λ ratio by immunohistochemistry and/or immunofluorescence requires a minimum of 100 plasma cells for analysis. An abnormal ratio reflecting presence of an abnormal clone is κ/λ of >4:1 or <1:2.

d. Plasmacytoma measurements should be taken from the CT portion of the PET/CT, or MRI scans, or dedicated CT scans where applicable. For patients with only skin involvement, skin lesions should be measured with a ruler. Measurement of tumour size will be determined by the SPD.

e. Positive immunofixation alone in a patient previously classified as achieving a complete response will not be considered progression. For purposes of calculating time to progression and progression-free survival, patients who have achieved a complete response and are MRD-negative should be evaluated using criteria listed for progressive disease. Criteria for relapse from a complete response or relapse from MRD should be used only when calculating disease-free survival.

f. In the case where a value is felt to be a spurious result per physician discretion (e.g., a possible laboratory error).

**Appendix 4. Response Evaluation of Multiple Myeloma – MRD Response Criteria**

| **Type of Response** | **Response Criteria** |
| --- | --- |
| Sustained MRD-negative | MRD negativity in the marrow (NGF or NGS) and by imaging as defined below, confirmed by two negative tests collected at least 1 year apart. Subsequent evaluations can be used to further specify the duration of negativity (e.g., MRD-negative at 5 years) |
| Flow MRD-negative | Absence of phenotypically aberrant clonal plasma cells by NGF on bone marrow aspirates using the EuroFlow standard operation procedure for MRD detection in multiple myeloma (or validated equivalent method) with a minimum sensitivity of 1 in 10⁵ nucleated cells or higher |
| Sequencing MRD-negative | Absence of clonal plasma cells by NGS on bone marrow aspirate in which presence of a clone is defined as less than two identical sequencing reads obtained after DNA sequencing of bone marrow aspirates using the LymphoSIGHT platform (or validated equivalent method) with a minimum sensitivity of 1 in 10⁵ nucleated cells or higher |
| Imaging-positive MRD-negative | MRD negativity as defined by NGF or NGS plus disappearance of every area of increased tracer uptake found at baseline or a preceding PET/CT or decrease to less mediastinal blood pool SUV or decrease to less than that of surrounding normal tissue |
| Relapse from MRD negative | Any one or more of the following criteria:  -Loss of MRD negative state (evidence of clonal plasma cells on NGF or NGS, or positive imaging study for recurrence of myeloma);  -Reappearance of serum or urine M-protein by immunofixation or electrophoresis;  -Development of ≥5% clonal plasma cells in the bone marrow;  -Appearance of any other sign of progression (ie, new plasmacytoma, lytic bone lesion, or hypercalcaemia) |

**Appendix 5. Definition of Lines of Therapy**

Line of Therapy (from International Myeloma Working Group (Rajkumar, 2011)) is defined as one or more cycles of a planned treatment program. This may consist of one or more planned cycles of single-agent therapy or combination therapy, as well as a sequence of treatments administered in a planned manner. For example, a planned treatment approach of induction therapy followed by autologous stem cell transplantation, followed by maintenance is considered one line of therapy. Each subsequent line of therapy starts when a planned course of therapy is modified to include other treatment agents (alone or in combination) as a result of disease progression, relapse, or toxicity. A new line of therapy also starts when a planned period of observation of therapy is interrupted by a need for additional treatment for the disease.
