# Supplementary material for: A novel two-step administration of XPO-1 inhibitor may enhance the effect of anti-BCMA CAR-T in relapsed/refractory extramedullary multiple myeloma
Source: J Transl Med. 2023 Nov 15;21:812. doi: 10.1186/s12967-023-04655-w (PMC10647128; doi:10.1186/s12967-023-04655-w)
Supplement: Supplementary file 4 — Additional file 4. Supplemental Materials. [file 12967_2023_4655_MOESM4_ESM.docx]

**Supplemental Materials**

This Supplemental Materials is provided by the authors for additional information about their work.

Supplements to Di Wang*, et al.* A novel two-step administration of XPO-1 inhibitor may enhance the effect of anti-BCMA CAR-T in relapsed/refractory extramedullary multiple myeloma

**CONTENTS:**

**Supplemental Results**

Detailed description of the two cases before infusion

**Figure legend of additional files**

Additional file 1: Fig. S1. Change of pleural effusion in patient 2 before and after treatment

Additional file 2: Fig. S2. Change of M protein and serum free light chain in patient 2 before and after treatment

**Supplemental Results**

***Detailed description of the two cases at enrollment***

Case 1

Patient 1 was a 55-year-old male who was treated in our center in September 2020, having been diagnosed with multiple myeloma (IgG-κ type) in his local hospital. The patient had multiple bone lesions at diagnosis, including lesions in his left tibia and several intracranial masses (largest about 20 × 15mm). The induction regimen was PAD (bortezomib, doxorubicin, dexamethasone) for two courses, but the size of extramedullary masses was not reduced. The VRD (bortezomib, lenalidomide, dexamethasone) regimen was then employed for re-induction. After four courses the patient achieved complete remission (CR), followed by stem cell mobilization. Unfortunately, the patient experienced an extramedullary relapse before they could receive autologous stem cell transplantation. Five courses of regimen DPD (daratumumab, pomalidomide, dexamethasone) + liposomal daunorubicin were given with best response being minimal response (MR), but the extramedullary lesion progressed after the last course. The patient came to our center for further medical intervention. He met the inclusion criteria and signed the informed consent to participate in this trial. At baseline, the mass on his head and left lower limb progressed to 76 × 49mm and 118 × 66mm (Fig. 1A), respectively, which incapacitated him due to severe pain. A cervical mass was also detected by PET/CT. The IgG-κ M protein level was 2.1g/L, but no plasma cells were detected in bone marrow or peripheral blood. The patient had decreased hemoglobin of 82g/L, normal renal and cardiac functions, and had no high-risk cytogenetic features.

Case 2

Patient 2 was a 55-year-old female who presented to her local hospital in July 2021 with lower back pain for a month. An intraspinal mass spanning T12 to L2 was found and surgically resected. The pathology confirmed that the mass was a plasmacytoma. Over 60% plasma cells were seen in her bone marrow and peripheral blood, secreting the IgD-λ type M protein.

This patient with EMM + PCL received several different regimens including PAD, VAD (vincristine, doxorubicin, dexamethasone) + lenalidomide, and DID (daratumumab, ixazomib, dexamethasone). The best response was MR, and her disease progressed quickly. She presented to our hospital and agreed to participate in this trial. The baseline assessment showed over 80% blasts in her bone marrow, but no plasma cells in her peripheral blood. A large bilateral pleural effusion (maximum depth: 9.9cm and 13.4cm on the left and right by ultrasonography, respectively) was detected, and 73% plasma blasts were found in the effusion. The IgD-λ type M protein level was 38.8g/L. The patient had decreased hemoglobin of 84g/L, increased serum creatine of 144umol/L and pro-BNP of 370pg/mL, and had no high-risk cytogenetic features.

***Bridging therapy***

Patient 1 received SD (Selinexor 60mg, dexamethasone 60mg and Daratuzumab 1000mg per week) regimen for three weeks as bridging therapy.

Patient 2 received SPD (Selinexor 60mg and dexamethasone 40mg per week; Pomalidomide 4mg per day) regimen for three weeks as bridging therapy.

**Supplemental Figures**

**Additional file 1: Fig. S1. Change of pleural effusion in patient 2 before and after treatment**

The upper part: CT scan showed bilateral massive pleural effusion of patient 1 at the layer near the bifurcatio tracheae before lymphodepletion; The lower part: CT scan showed no pleural effusion at 3-month post infusion at the similar layer with same photo-scale.

**Additional file 2: Figure S2. Change of M protein and serum free light chain in patient 2 before and after treatment.**

The serum M protein (red line) and serum free λ chain (green line) was measured at serial point post infusion. Both of them decreased significantly during the first 28 days post infusion, and remained at a low level thereafter.
